# Hippocampal Transcriptomic and Proteomic Alterations in the BTBR Mouse Model of Autism Spectrum Disorder

**DOI:** 10.3389/fphys.2015.00324

**Published:** 2015-11-24

**Authors:** Caitlin M. Daimon, Joan M. Jasien, William H. Wood, Yongqing Zhang, Kevin G. Becker, Jill L. Silverman, Jacqueline N. Crawley, Bronwen Martin, Stuart Maudsley

**Affiliations:** ^1^Metabolism Unit, National Institute on Aging, National Institutes of HealthBaltimore, MD, USA; ^2^Gene Expression and Genomics Unit, National Institutes of HealthBaltimore, MD, USA; ^3^Laboratory of Behavioral Neuroscience, Intramural Research Program, National Institute of Mental HealthBethesda, MD, USA; ^4^MIND Institute, University of California Davis School of MedicineSacramento, CA, USA; ^5^Receptor Pharmacology Unit, National Institute on Aging, National Institutes of HealthBaltimore, MD, USA; ^6^Translational Neurobiology Group, VIB Department of Molecular Genetics, University of AntwerpAntwerp, Belgium; ^7^Laboratory of Neurogenetics, Institute Born-Bunge, University of AntwerpAntwerpen, Belgium

**Keywords:** autism spectrum disorder phenotype, BTBR mouse model, bioinformatics, etiology, behavior

## Abstract

Autism spectrum disorders (ASD) are complex heterogeneous neurodevelopmental disorders of an unclear etiology, and no cure currently exists. Prior studies have demonstrated that the black and tan, brachyury (BTBR) T+ Itpr3tf/J mouse strain displays a behavioral phenotype with ASD-like features. BTBR T+ Itpr3tf/J mice (referred to simply as BTBR) display deficits in social functioning, lack of communication ability, and engagement in stereotyped behavior. Despite extensive behavioral phenotypic characterization, little is known about the genes and proteins responsible for the presentation of the ASD-like phenotype in the BTBR mouse model. In this study, we employed bioinformatics techniques to gain a wide-scale understanding of the transcriptomic and proteomic changes associated with the ASD-like phenotype in BTBR mice. We found a number of genes and proteins to be significantly altered in BTBR mice compared to C57BL/6J (B6) control mice controls such as BDNF, Shank3, and ERK1, which are highly relevant to prior investigations of ASD. Furthermore, we identified distinct functional pathways altered in BTBR mice compared to B6 controls that have been previously shown to be altered in both mouse models of ASD, some human clinical populations, and have been suggested as a possible etiological mechanism of ASD, including “*axon guidance”* and “*regulation of actin cytoskeleton.”* In addition, our wide-scale bioinformatics approach also discovered several previously unidentified genes and proteins associated with the ASD phenotype in BTBR mice, such as Caskin1, suggesting that bioinformatics could be an avenue by which novel therapeutic targets for ASD are uncovered. As a result, we believe that informed use of synergistic bioinformatics applications represents an invaluable tool for elucidating the etiology of complex disorders like ASD.

## Introduction

Autism spectrum disorders (ASDs) are complex neurodevelopmental disorders characterized by altered functionality across two symptom domains: (1) social and communication deficits; and (2) stereotyped repetitive behaviors with restricted interests (American Psychiatric Association, 2000). Currently, more than 1 in 100 children in the United States are diagnosed with an ASD (Nazeer and Ghaziuddin, 2012); therefore, understanding the origins of these disorders is a pressing health concern. Gene mutations have been found to play a large role in the onset of ASD (Abrahams and Geschwind, 2008). Yet while the heritability of ASD is high (nearly 90% by some estimates; Santangelo and Tsatsanis, 2005), genetic mutations known to result in the appearance of the ASD phenotype have been identified in only 30% of all ASD cases (Sakai et al., 2011). While several potential causes of ASD have been suggested (Chugani, 2004; Geschwind and Levitt, 2007; Pizzarelli and Cherubini, 2011; Zou et al., 2011; Choudhury et al., 2012), the exact etiology of ASD has yet to be elucidated (Persico and Bourgeron, 2006; Abrahams and Geschwind, 2008).

Given the heterogeneous nature of ASD, mouse models have proven to be particularly useful and reliable in elucidating the etiology of these disorders (Ey et al., 2011; Spooren et al., 2012; Tsai et al., 2012; Silverman and Crawley, 2014). One strain in particular, called the black and tan, brachyury, has been shown to be an especially relevant animal model of ASD (Bolivar et al., 2007; Moy et al., 2007). BTBR T+ Itpr3tf/J is an inbred strain of the black and tan, brachyury mice. This inbred strain has not only been useful in ASD research, but has been used as a model for type 2 diabetes (Clee et al., 2005), due to its natural insulin resistance. Extensive behavioral characterization of the BTBR mouse model has revealed low sociability compared to C57BL/6J (B6) mice (Bolivar et al., 2007; McFarlane et al., 2008). B6 is a standard inbred strain that shows normal social behaviors and low repetitive behaviors and is, therefore, frequently used as a good control comparison to BTBR (Bolivar et al., 2007; Moy et al., 2007, 2008; McFarlane et al., 2008; Pobbe et al., 2010; Defensor et al., 2011; Pearson et al., 2011; Scattoni et al., 2011, 2013; Wöhr et al., 2011; Silverman et al., 2012, 2015). Many other hallmark symptoms of ASD have also been observed in the BTBR mouse; moreover, behavioral phenotypes that are representative of both ASD symptomatic domains, including low sociability compared to B6 strains (Bolivar et al., 2007; Moy et al., 2007), resistance to change (Moy et al., 2007, 2008), increased display of repetitive self-grooming behavior (Pobbe et al., 2010), display of other repetitive behaviors (Pearson et al., 2011), and reduced display of territorial scent marking (Wöhr et al., 2011). Furthermore, unusual vocalizations have also been extensively characterized in BTBR mice (Scattoni et al., 2008, 2011), as well as instances of social avoidance and gaze aversion (Defensor et al., 2011). While the autistic-like behavioral phenotype of the BTBR mouse has been studied intensively, and preliminary genetic investigations of the differences between BTBR and B6 have been reported (McFarlane et al., 2008; Jones-Davis et al., 2013; Jasien et al., 2014), the precise transcriptomic and proteomic brain alterations underlying some of these ASD behavioral phenotypes remain unclear.

The specific genomic and transcriptomic mediators of the autistic phenotype are only now being revealed (Jones-Davis et al., 2013; Jasien et al., 2014). To enhance our understanding of the BTBR mouse strain and to gain further insight into the underlying mechanisms of ASD, we conducted transcriptomic and quantitative proteomic analyses on cortical and hippocampal tissues collected from BTBR mice, since these two brain regions have been strongly associated with ASD (Mundy, 2003; Nadler et al., 2006).

## Materials and methods

### Animal care and tissue collection

All experimental animal procedures were approved by the Animal Care and Use Committee of the National Institute on Aging. All mice used were either male BTBR T+Itprtf/J mice (4 months of age) or male control B6 C57BL6J mice (4 months of age) which were housed in the National Institute of Mental Health animal facility on a 12-h light and dark cycle from 6 a.m. to 6 p.m. Animals received food and water *ad libitum* throughout the duration of the study. Animals were euthanized using isoflurane anesthesia at 4 months of age and whole hippocampal and cortical tissues were collected by microdissection on a pre-chilled (4°C) metal plate performed by a trained researcher. Body weight data was collected immediately prior to euthanization for each animal. To prepare tissues for further analyses (protein or microarray) hemi hippocampi or cortices were further sliced on the pre-chilled plate using a pre-chilled sterile razor blade to generate a crude tissue homogenate. These tissues were subsequently snap frozen on dry ice and stored at −80°C until used for further analyses. Trunk blood was collected from each animal, blood was centrifuged at 3000 rpm for 30 min at 4°C and plasma was subsequently collected. Animal care and experimental procedures followed NIH guidelines and were approved by the National Institute on Aging Animal Care and Use Committee (protocol numbers 432-LCI-2015, and 433-LCI-2015).

### Identification and quantification of significantly altered genes

RNA was isolated from microdissected hippocampus and cortex from three individual animals in each experimental group (B6 control and BTBR, hippocampus and cortex) using a Qiagen RNeasy mini kit according the manufacturer's instructions (Qiagen, Inc., Valencia, CA). Therefore, three individual arrays for both cortex and hippocampus were performed for each genotype, BTBR and B6 control. Total RNA was used to generate biotin-labeled cRNA by using the Illumina TotalPrep RNA Amplification Kit (Ambion; Austin, TX, cat #IL1791). A total of 0.75 μg of biotin-labeled cRNA was hybridized at 58°C for 16 h to Illumina's Sentrix MouseRef-8 Expression Bead-Chips (Illumina, San Diego, CA). The arrays were washed and blocked, and the labeled cRNA was detected by staining with streptavidin-Cy3. The arrays were scanned with an Illumina BeadStation 500 × Genetic Analysis Systems scanner and the image data were extracted using the Illumina BeadStudio software, Version 3.0. Microarray data were analyzed using DIANE 6.0, a spreadsheet-based microarray analysis program based on the SAS JMP7.0 system. Raw microarray data were subjected to filtering and z normalization and tested for significant changes as described previously (Jin et al., 2012). In brief, average values of the replicate spots of each transcript on the microarray were normalized by global normalization. The correction factor was calculated by dividing the sum of intensities of each sample by the average of all the samples. The normalized values were calculated by multiplying average intensities of each mRNA with the correction factor. Raw intensity data for each experiment were transformed to log_10_, and used for the calculation of Z-scores. Significant changes in mRNA expression were calculated in the form of Z-ratios and/or *Z*-test values, by using Z-score values in all calculations. Z-ratios constitute a measure of the change in transcript expression of a given gene from control group value, expressed in units of standard deviation from the average change of all genes for that comparison. The Z-ratio is a measure of fold change between comparisons, and the *p*-values test for reproducibility of the intensity of a gene among biological replicate arrays: Z-ratio (between condition A and B) = z(A) - z(B)/SD deviation). Remaining genes were analyzed by Two-way ANOVA to establish the statistical significance of differential levels of expression between ages and genotypes (*p* < 0.05). Comparisons between Z-ratios test for equivalence of significant changes between the BTBR groups and the control B6 groups. All transcript expression changes were assessed through comparison with control samples. A Z-ratio value of ± 1.50 and/or a *Z*-test value *p* < 0.05 were the significance thresholds used in this study. Significantly-regulated transcripts were then refined by calculating the false discovery rate, which controls for the expected proportion of falsely rejected hypotheses, and including only those genes with false discovery rate < 0.05. Hierarchical clustering and principal component analysis was performed with the software package DIANE 6.0, a spreadsheet-based microarray analysis program based on the SAS JMP7.0 system.

### Signaling pathway bioinformatics analysis

Functional signaling pathway analyses were used to analyze significantly regulated transcript and protein data sets from the control (B6) and BTBR mice. KEGG pathway analysis was performed using WebGestalt (http://bioinfo.vanderbilt.edu/webgestalt/) software, as previously described (Zhang et al., 2005; Wang et al., 2013). Canonical signaling pathway analysis was performed using Ingenuity Pathway Analysis (IPA). Inclusion criteria were set as follows: pathway groups needed to meet a minimum population of two transcripts/proteins from the input experimental set, and also needed to possess a probability significance of enrichment compared to a control background dataset of less than 0.05 (hypergeometric test of significance). For Kyoto Encyclopedia of Genes and Genomes (KEGG) pathway analysis, the degree of enrichment R was calculated and expressed as a hybrid score as follows: *R* = O/E where O is the observed gene number and E is the expected gene number in the KEGG pathway. *P*-values were assigned to pathways with *R* > 1 to indicate the significance of enrichment. For IPA canonical pathway analysis, the enrichment probability (expressed as a negative log_10_ of the probability) and enrichment ratio are indicated in the specific data tables and represent the direct primary output of the full signaling mode (Metabolic and Cellular Signaling) of pathway analysis. In addition, Venn diagrams were also constructed to identify common and uniquely altered genes between hippocampus and cortex, using VennPlex (Cai et al., 2013). Word frequency analysis was performed with the online WriteWords application (www.writewords.org.uk/word_count.asp).

### Latent semantic indexing analysis

Latent semantic indexing (LSI)-based GeneIndexer analysis was performed as previously described (Chadwick et al., 2010). Hence we used GeneIndexer software (www.computablegenomix.com, Memphis, TN) to rank genes/proteins based on relevancy to the input keyword queries using functional information in Medline (Homayouni et al., 2005). GeneIndexer contains over 2 million Medline abstracts corresponding to over 21,000 mammalian genes. GeneIndexer extracts both explicit and implicit gene/protein-to-keyword relationships from the literature using an information retrieval model called LSI (Homayouni et al., 2005; Roy et al., 2011; Chen et al., 2013a). This model ranks genes according to the strength of the association with the keyword query, whereby a score > 0.2 typically indicates an explicit association (e.g., the word actually appears in the gene abstracts) and a score between 0.1 and 0.2 typically indicates an implicit functional relationship (Homayouni et al., 2005). In brief, experimentally-derived gene symbol lists (from either hippocampus or cortex) were uploaded into GeneIndexer. Using LSI, GeneIndexer then correlates the strength of association between specific factors (genes/proteins) in these datasets with user-defined interrogation terms (“*autism,” “autistic disorder,” “autistic spectrum disorder,” “ADHD,” “ASD,” “obsessive”)*. Genes/proteins with a significant correlation (value > 0.1) were extracted and labeled (according to their experimentally-derived identified expression pattern, i.e., BTBR vs. B6 control) as either elevated (red) or decreased (green). *Textrous!-*based natural language processing analysis was performed, as previously described (Chen et al., 2013b). Essentially, *Textrous!* can perform the inverse function of GeneIndexer by deriving significantly correlated natural language nouns semantically-linked in multiple biomedical databases (PubMed Central Abstracts (http://www.ncbi.nlm.nih.gov/pubmed/) including Online Mendelian Inheritance in Man (http://www.omim.org/) and Jackson Laboratories Mouse Genomatics Mammalian Phenotypes Database (http://www.informatics.jax.org/searches/MP_form.shtml). *Textrous!* possesses two modes of dataset analysis: (1) “collective,” in which words correlating to all of the input data are derived; and (2) “individual,” in which the strongest individual input data-noun correlations are reported.

### Real-time polymerase chain reaction analysis

Real-time polymerase chain reaction (PCR) analysis was performed, as previously described (Shin et al., 2008). Briefly, total RNA was extracted from the microdissected (from three mice on each genotype) mouse hippocampus and cortex using TRIzol reagent and reverse-transcribed into cDNA using SuperScript™ First-Strand Synthesis System (Invitrogen, Grand Island, NY). Next, PCR was carried out using gene-specific primer pairs and SYBR Green PCR master mix (Applied Biosystems, Foster City, CA) in an ABI Prism 7000 sequence detection system (Applied Biosystems). The amplification conditions were 50°C (2 min), 95°C (10 min), and then 40 cycles at 95°C (15 s) and 60°C (1 min). The data were normalized to glyceraldehyde-3-phosphate dehydrogenase (Gapdh) mRNA. All real-time PCR analyses are represented as the mean ± S.E. from at least three independent animal experiments, each performed in triplicate. Primers used were: 5′- TCATACTTCGGTTGCATGAAGG-3′ and 5′- AGACCTCTCGAACCTGCCC-3′ for BDNF; 5′- GAAACACCAGCACTATGATTGGA-3′ and 5′- ATTCCCGTAAACTCCCCTGTG-3′ for Pak1; 5′- ATTTGTCCCAATGTCTGCGAA -3′ and 5′- TGGCTATCTTGGCTATAAAGGGG -3′ for Serpina3n; 5′-TCTGACTTTCCTTGCCTGGT-3′ and 5′-ATTCAGGTCTCGTTGGCATC-3′ for Cort; 5′-GAAGTTCGCCTGCTTTGAAC-3′ and 5′-CTGCCACAAATGTCACAACC-3′ for Slc25a3.

### Western blotting protein expression analysis

Microdissected hippocampal and cortical tissues (*n* = 4 B6 controls, *n* = 4 BTBR) were fractionated using the Qproteome™ Cell Compartment Kit according to the manufacturer's instructions (Qiagen, Valencia, CA). Protein extracts were quantified using BCA reagent (ThermoScientific, Rockford, IL) before one-dimensional mass-based resolution. Specifically protein extracts (containing 15 μg of total protein) were resolved on 4–12% Bis-Tris polyacrylamide gels (Invitrogen, Carlsbad, CA) before electrotransfer to a polyvinylenedifluoride (PVDF) membrane (Perkin Elmer, Waltham, MA). PVDF membranes were blocked for 1 h at room temperature in 4% non-fat milk (Santa Cruz Biotechnology, Santa Cruz, CA) before immunoblotting. Specific primary antisera were obtained from the following sources: Actin (Sigma Aldrich, St. Louis, MO), Stxbp1 (Sigma Aldrich, St. Louis, MO), Rock2 (Abcam, Cambridge, MA), Tom1I2 (Sigma Aldrich, St. Louis, MO), Agk (Novus Biologicals, Littleton, CO), and Gap43 (Abcam, Cambridge, MA), Arl1 (Proteintech Group, Inc., Chicago, IL) Detection of primary immune complexes were performed with subsequent application of a 1:10,000 dilution of an alkaline phosphatase-conjugated, species-specific secondary antibody (Sigma Aldrich, St. Louis, MO) followed by enzyme-linked chemifluorescence (ECF) exposure (GE Healthcare, Pittsburgh, PA) and digital quantification using a GE Amersham Molecular DynamicsTyphoon 9410 Phosphorimager with ImageQuant 5.2 L software (GE Healthcare, Pittsburgh, PA). ECF band intensity was measured as fluorescent units minus background per square pixel ((FU-B)/px^2^).

### Isobaric tags for relative and absolute quantification mass spectrometry

Isobaric tags for relative and absolute quantification (iTRAQ) mass spectrometric quantification was performed, based on a modified protocol to that described previously (Hu et al., 2006). Briefly, iTRAQ isobaric mass tags and labeling reagents were obtained from Applied Biosystems (Carlsbad, CA). BTBR and B6 tissue samples (*n* = 4 BTBR and *n* = 4 B6 control) were treated in parallel throughout the labeling procedure. Hippocampal tissues were fractionated using the Qproteome™ Cell Compartment Kit according to the manufacturer's instructions (Qiagen, Valencia, CA). Protein extracts representing the cytosolic compartment were used primarily for subsequent proteomic and immunoblotting analyses. This compartment was chosen as it represents the most diverse cellular compartment and therefore the most likely to yield information pertaining to as many molecular signaling processes as possible. These protein extracts were quantified using BCA reagent (ThermoScientific, Rockford, IL). The general iTRAQ labeling protocol consists of: protein reduction and cysteine blocking, protein digestion via trypsin, peptide labeling with iTRAQ reagents, sample combination, strong cation exchange chromatography, desalting with solid phase extraction, and liquid chromatography with tandem mass spectrometric analysis. Briefly, to create our two control iTRAQ samples, 75 μg of protein from two B6 control mice were pooled to and then were designated to be labeled with the 114 mass iTRAQ label mixture—the same procedure using the pooling of two additional separate 75 μg B6 control protein samples was performed to create the second 115 control mass iTRAQ label mixture. In a similar manner the protein extracts from four BTBR mice were evenly distributed into the 116 and 117 mass iTRAQ label groups. Hence the four individual 150 μg protein extracts from each mouse genotype (control B6 114, 115—BTBR 116, 117 iTRAQ labels) were acetone precipitated and resuspended in 20 μL of iTRAQ dissolution buffer [0.5 M triethylammonium bicarbonate (TEAB), ABSciex] containing 0.1% ProteaseMAX detergent (Promega) to denature the proteins. The sample was then reduced by adding iTRAQ Reducing Reagent [Tris(2-carboxyethyl) phosphine (TCEP), ABSciex] to a final concentration of 5 mM and incubated at 60°C for 1 h. Subsequently, the sample was alkylated with iTRAQ Cysteine-Blocking Reagent [10 mM methyl methanethiosulfonate (MMTS), ABSciex] for 10 min at room temperature. The protein samples were then digested with 5 μg sequencing-grade trypsin (Promega) per 100 μg protein at 37°C overnight. Labeling of the samples with iTRAQ labels was performed at room temperature for 2 h. After labeling, the samples to be compared were mixed and underwent an off-line strong cation exchange (SCX) fractionation (ICAT Cation Exchange Buffer Pack, ABSciex) to 16 fractions to reduce input MS sample complexity. After reversed-phase desalting (C18 tips, Pierce Biotechnology), the samples were re-constituted in water with 0.1% formic acid, then stored at −20°C until LC/MS/MS analysis. Western blotting procedures for validation were performed using the same individual protein lysates employed for initial iTRAQ labeling.

### LC/MS/MS analysis

Samples were analyzed using an Eksigent NanoLC Ultra 2D (Dublin, CA) and Thermo Fisher Scientific LTQ Orbitrap XL (San Jose, CA). In brief, peptides were first loaded onto a trap cartridge (Agilent), then eluted onto a reversed phase PicoFrit column (New Objective, Woburn, MA) using a linear 120 min gradient of acetonitrile (2–62%) containing 0.1% formic acid at 250 nL/min flowrate. The eluted peptides were sprayed into the LTQ Orbitrap XL. The data-dependent acquisition mode was enabled, and each FTMS MS1 scan (60,000 resolution) was followed by 6 MS2 scans (alternating CID at unit resolution and HCD at 7500 resolution on 3 precursor ions). The spray voltage and ion transfer tube temperature were set at 1.8 kV and 180°C, respectively.

### Database search and iTRAQ quantification

Proteome Discoverer 1.2 (Thermo Fisher Scientific) was used for protein identification and iTRAQ quantification using Sequest algorithms. The following criteria were followed: SwissProt mouse database; enzyme: trypsin; miscleavages: 2; static modifications: methylthio (+45.988 Da on C), iTRAQ8plex (+304.205 Da on N-terminus and K); dynamic modifications: oxidation (+15.995 Da on M), deamidation (+0.984 Da on N and Q); peptide tolerance as 25 ppm; MS2 tolerance as 0.8 Da. Peptides reported via all search engines were accepted only if they met the false discovery rate of 5% and a protein group identification confidence of 99%. For iTRAQ quantification, the reporter ion intensities in MS2 spectra (*m*/z 114–117, integration width tolerance 50 mmu) were used to calculate the expression ratios among the different conditions (Hippocampus: WT and BTBR). Initial protein identification lists were then subjected to additional quality controls. Hence, confidence limits of acceptable control sample (114, 115 labels) variation from labeling accuracy were set (as a ratio of 114:115 label) as >0.8 and < 1.2. Only proteins identified against such control sample MS2 spectra were considered for further analysis—quantified proteins generated from analysis against non-compliant 114:115 ratio samples were discarded. Proteins quantified relative control levels meeting these criteria were next filtered for 116:(114:115) or 117:(114:115) ratios outside the following limits, < 0.8 and >1.2. Therefore, such proteins were accurately and reliably and differentially co-identified in the BTBR compared to the control B6 tissues.

### Plasma hormone analysis

Plasma insulin, leptin, gastric inhibitory polypeptide (GIP, total), pancreatic polypeptide (PP), and peptide YY (PYY) were measured using a Linco-Millipore 5-plex kit (EMD Millipore, Billerica MA) with a Bio-Plex® 200 suspension array system (Bio-Rad, Hercules CA). Blood samples from BTBR or B6 mice were obtained by exsanguination after the ethical isoflurane euthanasia process. Whole blood was collected (from *n* = 3 animals per genotype each) in EDTA-containing vacutainer tubes (BD Bioscience) to prevent coagulation. Blood was then centrifuged at 12,500 rpm for 12 min at 4°C and plasma supernatant was removed and stored at −80°C. Prior to hormone assays the appropriate standard curve solutions were set up for accurate quantitation. Multiplexed hormone analyses were performed using a 96-well plate format. Briefly, 200 μl of proprietary Assay Buffer is added to each well, the plate is then shaken for 10 min at room temperature before being decanted off. Then 25 μl of either hormone standard or control sample is added to the appropriate wells. Twenty five microliter of Assay Buffer is then added to the background and sample wells. Next 25 μl of the appropriate matrix solution is added to background, standards and control wells followed by the addition of 25 μl of neat samples to the sample wells and then 25 μl of proprietary Linco-Millipore beads to each well followed by overnight incubation at 4°C. After incubation the individual well contents were removed and the wells were washed three times with 200 μl of the proprietary wash buffer followed by the subsequent addition of 50 μl of detection antibodies per well (insulin, leptin, GIP, PP, and PYY) and incubation for 1 h at room temperature. Next 50 ml of streptavidin-phycoerythrin was added per well and allowed to incubate for a further 30 min at room temperature. Following this well contents were removed and the wells were washed three times with 200 μl of wash buffer. Before quantitation using the Bio-Plex 100 μl of proprietary Sheath Fluid was added per well. Each sample was assayed in duplicate on a 96-well plate. Analysis of quality control standards provided in the kits met expectations, validating the accuracy of the panels. In addition to the metabolic hormone panel plasma corticosterone concentration was measured using Corticosterone Double Antibody RIA Kit (MP Biomedicals Solon, OH: # 07120103) according to manufacturer's instructions. Samples were run on a Packard Cobra II Gamma Counter.

### Statistical analysis

Statistical analyses were conducted, with GraphPad Prism v. 5.0, using a Student's *t*-test; *p* ≤ 0.05 was considered statistically significant throughout the study. Error bars represent 95% confidence intervals. All data represent means ± standard error of the mean.

## Results

### Significant alterations in BTBR hippocampal and cortical gene transcription compared to B6

**Figure 1:**
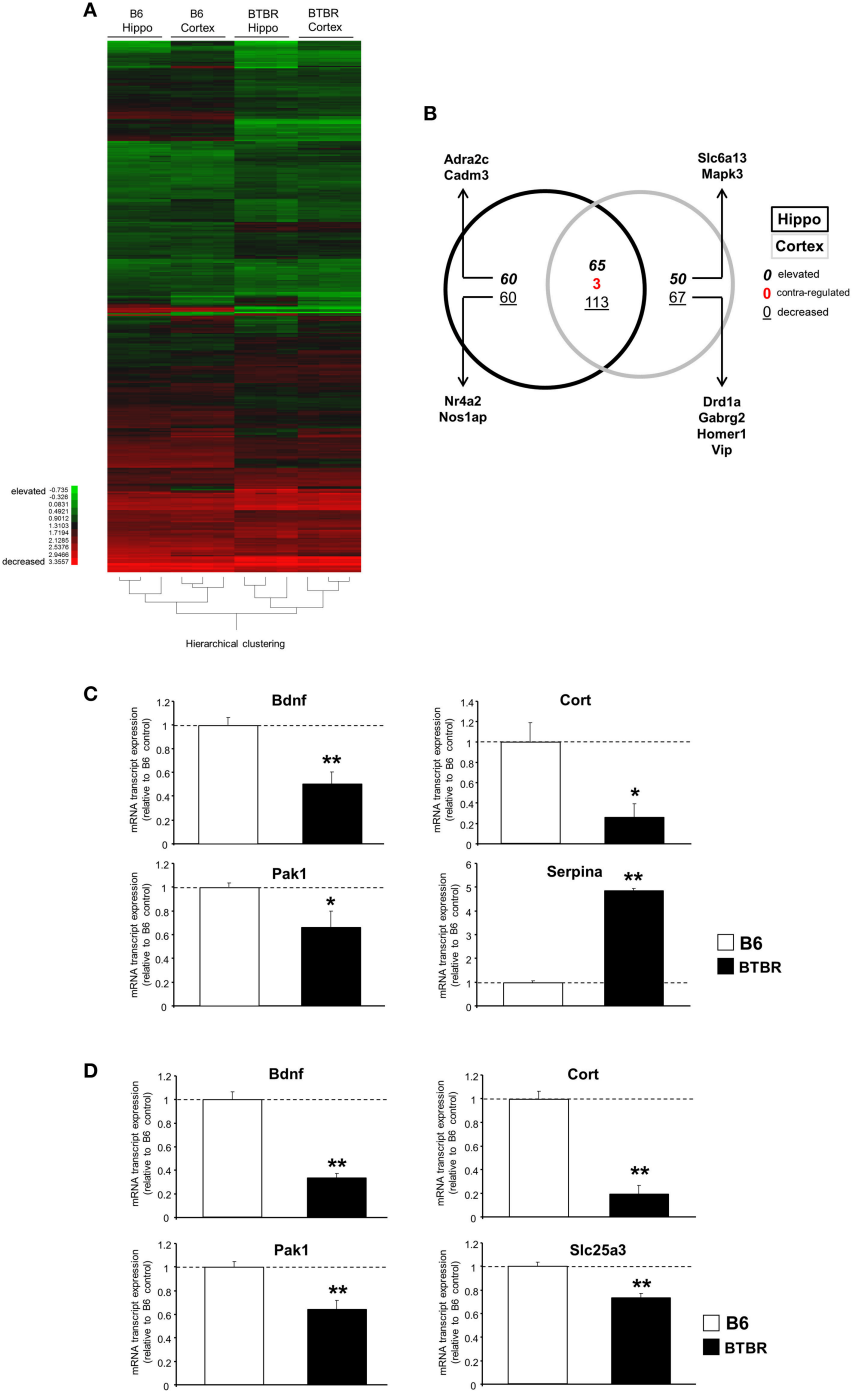
**Significant cortical and hippocampal transcriptomic divergence between BTBR and B6 controls**. **(A)** Heatmap of significantly decreased or elevated transcripts in cortical and hippocampal tissues from BTBR mice compared to tissues from B6 controls. Green blocks = decreased; red blocks = elevated. **(B)** VennPlex diagram of significantly regulated [up- (bold italic), down- (underlined), or contra-regulated (red bold: possessing a diverse expression polarity in different tissues)] transcripts in hippocampal (black line) or cortical (gray line) tissues. Arrows indicate specific transcripts of interest to autistic phenotypes. RT-PCR-mediated validation of significantly-regulated hippocampal **(C)** and cortical **(D)** transcripts. Histogram white bars represent B6 data and black bars represent BTBR data.^*^p ≤ 0.05, ^**^p ≤ 0.01.

Microarray analysis was performed on BTBR and B6 hippocampal (Table S1) and cortical tissues (Table S2). Using k-means hierarchical clustering (Chadwick et al., 2011) we found that both B6 and BTBR tissues specifically clustered according to genotype (Figure 1A). In addition to the k-means clustering, we also performed Principal Component Analysis on all of the transcriptomic datasets and again demonstrated a strong global divergence between B6 and BTBR mouse cortex/hippocampus tissues (Figure S1). With respect to comparing the differences in BTBR cortex and hippocampal tissues (both compared to their respective B6 controls) we found, using VennPlex analysis (Table S3) that there was a strong and coherently-regulated (i.e., expression polarity retained between tissues) overlap (65 transcripts co-elevated and 113 transcripts co-decreased) between the BTBR cortex and hippocampus (Figure 1B). We performed multiple PCR-based validations on selected transcript targets (brain-derived neurotrophic factor [Bdnf], p21-activated kinase type 1 [Pak1], cortistatin [Cort], solute carrier family 25 [mitochondrial carrier; phosphate carrier], member 3 [Slc25a3], Serpin peptidase inhibitor, clade A [Serpina]: chosen to represent both elevated and decreased transcripts; Figure 1C—hippocampus; Figure 1D—cortex). Each of these validations corroborated our array-based transcriptomic data.

### Unbiased bioinformatics approaches reveal specific alterations of functional groups and molecular pathways in BTBR mice

**Figure 2:**
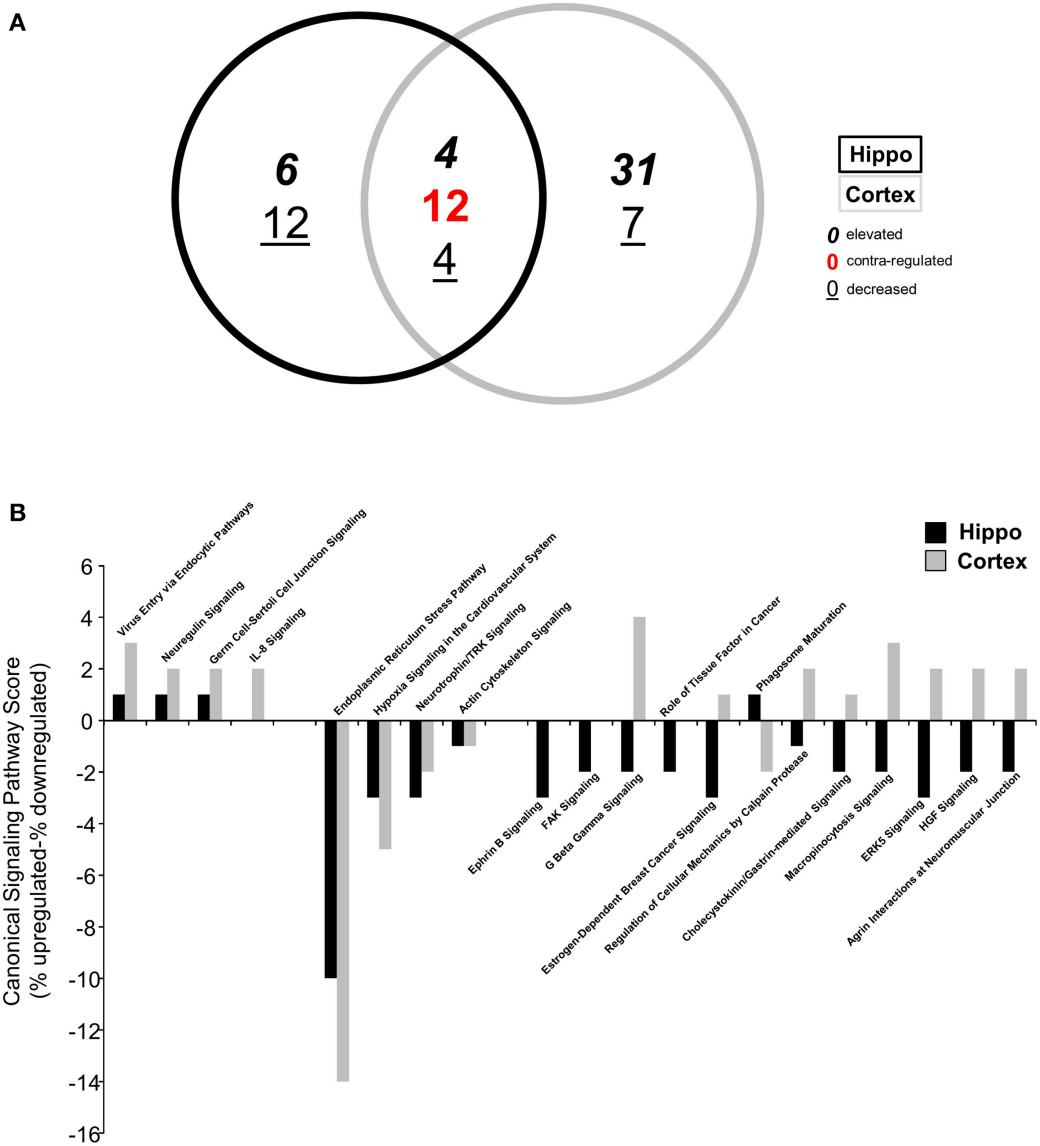
**Differential canonical signaling pathway analysis of BTBR-specific hippocampal and cortical transcriptomes**. **(A)** VennPlex representation of IPA canonical signaling pathway annotation of hippocampal (black line) and cortical (gray line) transcriptomic data (VennPlex numerical set description is as described in Figure 1). **(B)** Differential IPA signaling pathway scores (% elevated transcripts in given pathway minus % decreased transcripts in the given pathway).

**Figure 3:**
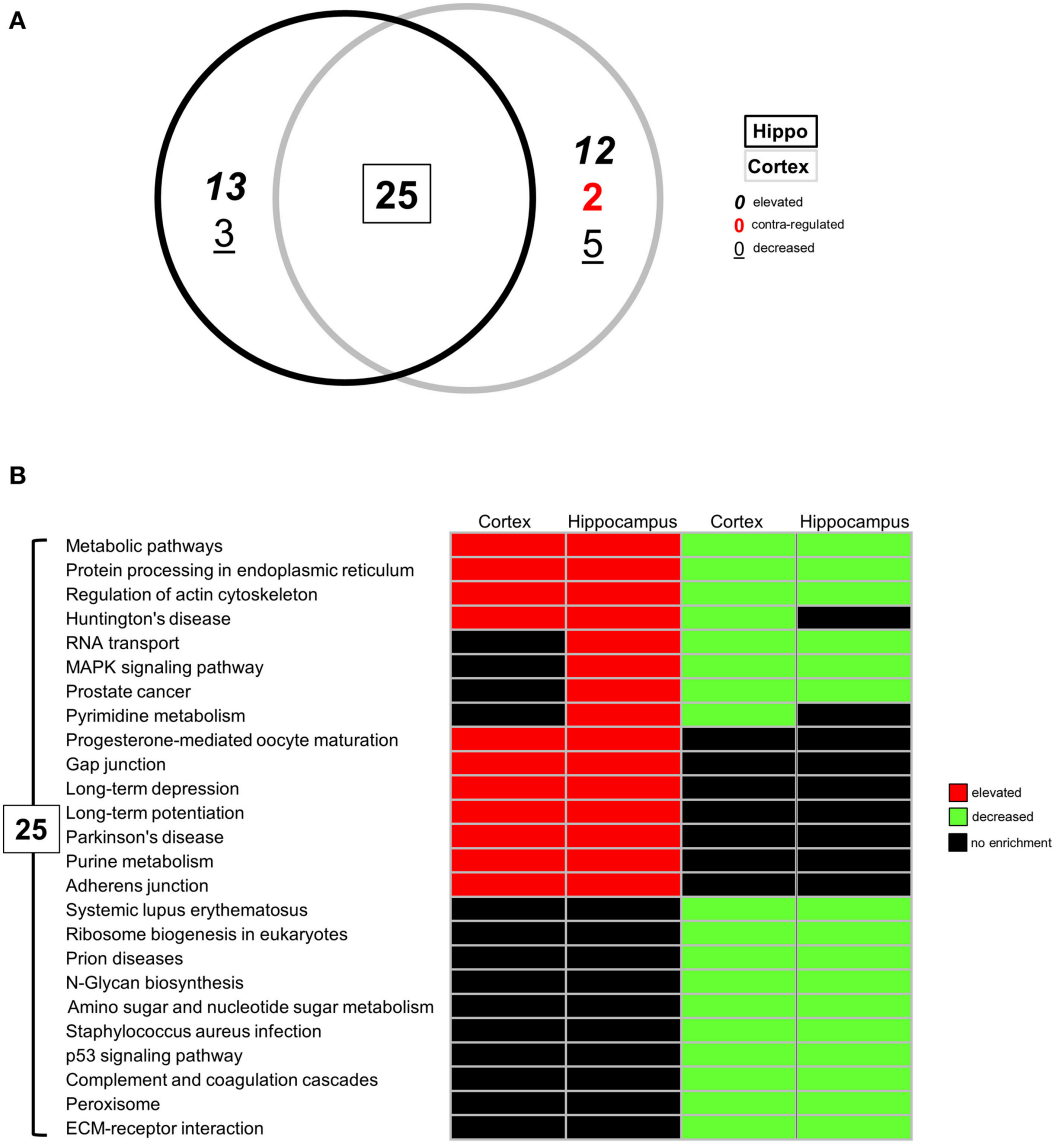
**Differential KEGG pathway analysis of BTBR-specific hippocampal and cortical transcriptomes**. **(A)** VennPlex representation IPA canonical signaling pathway annotation of hippocampal (black line) and cortical (gray line) transcriptomic data (VennPlex numerical set description is as described in Figure 1). **(B)** Differential KEGG pathway population matrix of 25 **(A)** commonly-controlled pathways between hippocampal and cortical tissues. Differential pathway regulation (elevated—red; decreased—green) was generated using data from up- or downregulated input transcriptomic sub-datasets.

Following identification of significantly up- or downregulated transcriptomic alterations, our next step was to determine if coherent functional groups could be constructed from the BTBR transcriptomic data and how these then potentially relate to autistic-related pathophysiology (Figure 2). First, we employed IPA-based canonical pathway analysis of both the hippocampal (Table S4) and cortical (Table S5) transcriptomic datasets. As with the transcript identity analysis (Figure 1B), we found a considerable functional pathway overlap between the two tissues. Using a pathway scoring system assessing the proportional up- or downregulated balance of the populated pathway (positive scores indicate a high degree of elevated transcripts, negative scores indicate a high degree of decreased transcripts) we found that many of the common pathways between the hippocampus and cortex were indeed contra-regulated, suggesting a functional divergence between these tissues in the BTBR mice (Figure 2A). Upon inspection of the common signaling pathways (Figure 2B), it was clear that the BTBR phenotype is underpinned across both tissues by alterations in neurotrophin signaling, cytoskeletal alterations, and endoplasmic reticulum stress. In contrast to the cortex, the BTBR hippocampus appeared to possess a phenotype characterized by reductions in multiple receptor signaling systems (*ephrin B, G protein beta-gamma signaling, cholecystokinin/gastrin, and HGF*), as well as structural modifications (*FAK signaling and macropinocytosis signaling*). To complement our IPA pathway analysis, we also applied KEGG pathway annotation (using up- or downregulated datasets separately) to the BTBR (relative to B6 in each case) hippocampal (Table S6) and cortical (Table S7) data sets. In accordance with the IPA analysis, we found a strong overlap (25 pathways) of signaling pathways significantly populated in both tissues (Figure 3A). For these common KEGG pathways, we found that several of these were populated by elevated or decreased transcriptome datasets in both tissues (*metabolic pathways, protein processing in the endoplasmic reticulum, and regulation of actin cytoskeleton*). Therefore, these pathways indicate a generic form of pathology across both tissues in the BTBR mice. In contrast to these bimodal KEGG pathways, many signaling pathways associated with neural activity were specifically upregulated (*gap junction, long-term depression and potentiation, Parkinson's disease, and adherence junction*), while the pathways populated by downregulated transcripts were strongly related to metabolic stress responses (*p53 signaling pathway, peroxisome, complement and coagulation cascades, and ribosome biogenesis in eukaryotes*; Figure 3B). Our ability to identify functional groups from our experimental transcriptome datasets strongly suggests that these functional groups potentially play a role in the presentation of the ASD-like phenotype in BTBR mice. Consequently, bioinformatics can possibly be used to detect specific functional groups and signaling pathways responsible for the ASD-like phenotype observed in BTBR mice.

**Figure 4:**
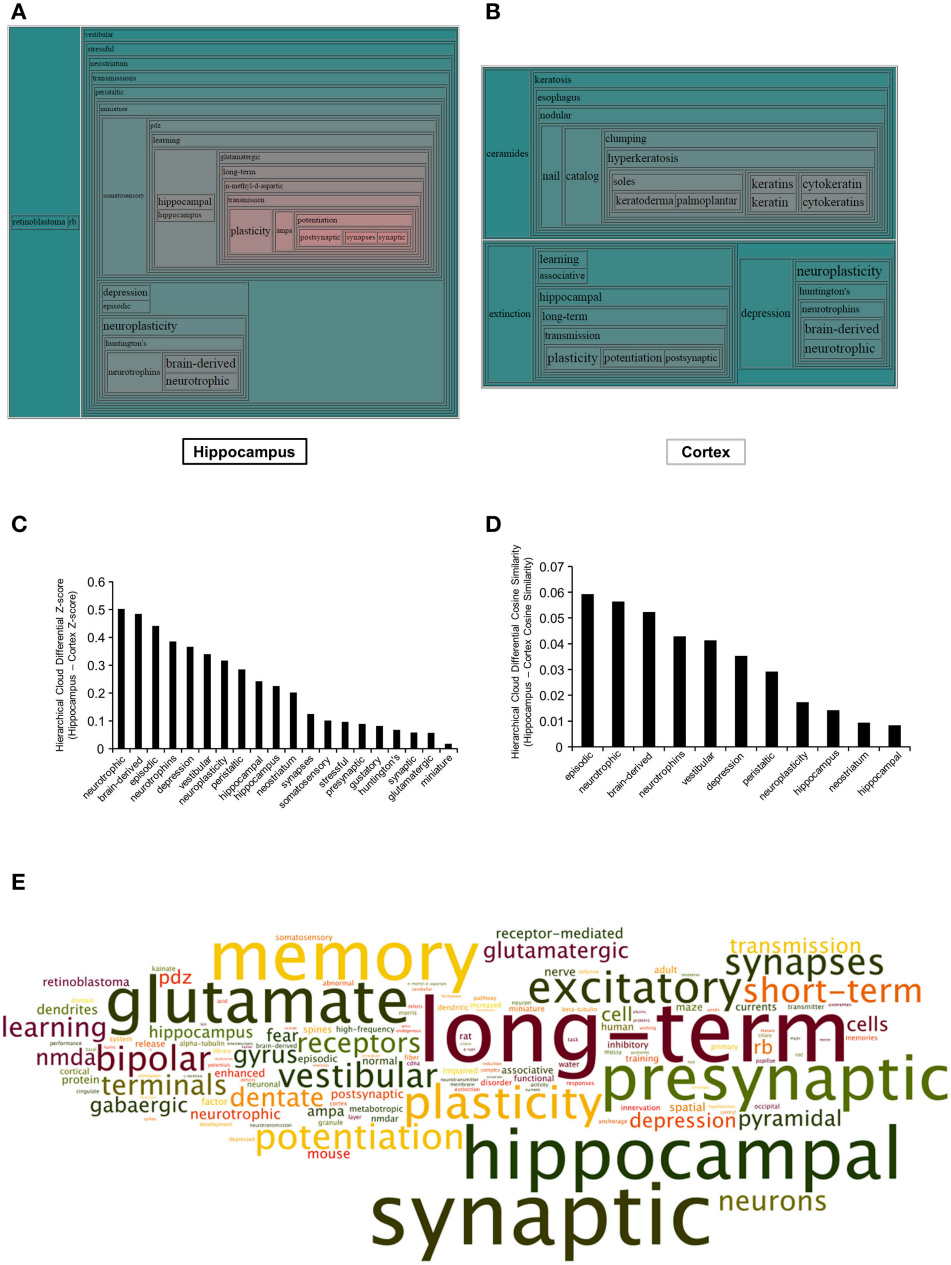
***Textrous!-*based hierarchical wordcloud investigation of BTBR-specific hippocampal and cortical transcriptomes**. Hierarchical wordcloud generated using Textrous! Collective Processing of significantly-regulated hippocampal **(A)** or cortical **(B)** transcripts. Regional proximity of words within the hierarchical wordcloud indicates synergistic functions: increased word text size and increased red hue indicates strength of association between words and the collective contents of the input dataset. Differential Z-score **(C)** and Cosine Similarity score **(D)** analysis between words commonly associated with both hippocampal and cortical input transcriptomic datasets. **(E)** Specific hippocampal global Collective Processing dismantled noun and noun-phrase word cloud: text size indicates relative word occurrence score (text color is randomly assigned).

In addition to performing a curated pathway analysis (KEGG and IPA), we also wished to employ an orthogonal natural language processing-based informatics approach to more effectively appreciate the functional phenotypic status of the BTBR mice. Therefore, we used our previously developed natural language processing-based platform, *Textrous!* (Chen et al., 2013b) in the collective processing mode to generate hierarchical wordclouds, using natural language nouns extracted from multiple biomedical text resources (PubMed Abstracts (http://www.ncbi.nlm.nih.gov/pubmed/) including Online Mendelian Inheritance in Man (http://www.omim.org/) and Jackson Laboratories Mouse Genomatics Mammalian Phenotypes Database (http://www.informatics.jax.org/searches/MP_form.shtml). The use of freeform quantitative wordclouds to convey complex non-canonical signaling-activity relationships is becoming more and more recognized as a novel technique to investigate high-dimensionality data (Baroukh et al., 2011; Cheung et al., 2012; Lynch et al., 2015). The hierarchical wordclouds created using *Textrous!* collective processing from the hippocampus (*Textrous!* output: Table S8) and cortex (Textrous! output: Table S9) generated an extra level of inference with respect to the pathological alterations occurring in the two BTBR tissues (Figures 4A,B). Using the natural language processing-based *Textrous!* analysis, it became apparent that a greater neurotrophic/neurosynaptic phenotype is present in the hippocampus (Figure 4A). To quantify this finding from the hierarchical wordclouds, we assessed the nature of the nouns possessing a greater differential Z- and Cosine Similarity score in the hippocampus compared to the cortex (Figure 4C-Z-score: Figure 4D-Cosine Similarity). In both cases (Figures 4C,D), it became clear that nouns possessing greater hippocampal Z- and Cosine Similarity scores are tightly linked to neurosynaptic activity (*neurotrophic, brain-derived, neurotrophins, neuroplasticity, and synapses*). To more clearly crystallize this specific BTBR hippocampal phenotype, we extracted all the derived nouns and noun-phrases, manually dismantling them into individual nouns, forming a global textual cloud (Wordle [http://www.wordle.net/]: word occurrence scores were calculated using WriteWords [http://www.writewords.org.uk/word_count.asp]; Table S10). In this cloud structure, the size of the noun is correlated to the occurrence score measured from the dismantled noun phrases that are generated using *Textrous!* collective processing. Using this gestalt-level of natural language analysis, in which the impact of every significantly-altered transcript is considered, it is clear that a profound alteration in synaptic, memory, and plasticity functions are evident in this tissue (Figure 4E).

### Differential BTBR vs. control B6 hippocampal protein expression

**Figure 5:**
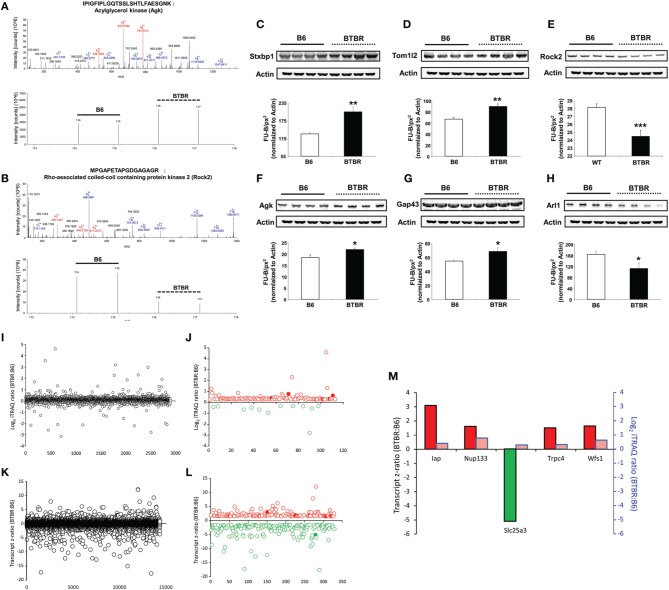
**iTRAQ- and transcriptomic-based identification and quantification of BTBR-modulated hippocampal proteins**. Identification of hippocampal proteins significantly up-regulated in BTBR mice compared to B6 controls via iTRAQ-based quantitative mass spectrometry. An example of the **(A)** isolation and identification of Agk MS^2^ peptide ions (*b* daughter ions in red, *y* daughter ions in blue) and below iTRAQ mass tag reporter ions of Agk peptides found to be elevated in BTBR mice compared to B6 controls. An example of the **(B)** isolation and identification of Rock2 MS^2^ peptide ions (*b* daughter ions in red, y daughter ions in blue) and below iTRAQ mass tag reporter ions of Rock2 peptides found to be lower in BTBR mice compared to B6 controls. Western blot validation of alterations in hippocampal protein expression levels. Validation of alterations in hippocampal protein expression levels in BTBR mice compared to B6 controls via Western blotting. Validation of mass spectrometric identification of altered protein expression levels of the following proteins: Stxbp1 **(C)**, Tom1l2 **(D)**, Rock2 **(E)**, Agk **(F)**, Gap43 **(G)**, and Arl1 **(H)**. Protein expression levels were measured as Fluorescent Units-Background fluorescence per square pixel (FU-B/px^2^) relative to Actin. ^*^p ≤ 0.05, ^**^p ≤ 0.01, ^***^p ≤ 0.001. White bars represent B6 data and black bars represent BTBR data. **(I)** Total hippocampal protein expression variation between BTBR and control B6 mice. **(J)** Reliable and differentially-regulated hippocampal proteins between BTBR and B6 mice (red circles indicated upregulated proteins, green circles indicate downregulated proteins: solid filled circles represent common factors identified at both protein and transcript level). **(K)** Total hippocampal transcript expression variation between BTBR and control B6 mice. **(L)** Reliable and differentially-regulated hippocampal transcripts between BTBR and B6 mice (red circles indicated upregulated proteins, green circles indicate downregulated proteins: solid filled circles represent common factors identified at both protein and transcript level). **(M)** Hippocampal proteins/transcripts commonly identified in both proteomic and microarray analyses. Bars outlined with black lines represent transcriptomic data and those outlined in blue represent proteomic data.

To further build upon the important transcriptomic differences in the BTBR hippocampus compared to B6 controls, we subsequently analyzed overall proteomic alterations in this tissue using iTRAQ-based quantitative mass spectrometry (Table S11). Using iTRAQ labeling of control B6 and BTBR hippocampal tissue we identified and generated iTRAQ ratios for 2907 proteins common between these two genotypes—from this reliable expression ratios of proteins expressed differentially expressed in BTBR mice hippocampi compared to control B6 hippocampi we found that 101 proteins were elevated and 12 were decreased in the BTBR hippocampus (Table S11). To exemplify the protein identification and quantitation process, two representative MS^2^ spectra (*b*- and *y*-ion mediated peptide identification) and iTRAQ reporter ion quantitative spectra (114, 115, 116, and 117 labels) are displayed in Figure 5A (acylglycerol kinase [Agk]) and Figure 5B (Rho-associated coiled-coil containing protein kinase 2 [Rock2]). Next, we confirmed our mass spectrometry results using selective western blots of proteins identified using iTRAQ as upregulated (syntaxin binding protein 1 [Stxbp1], TOM1-like protein 2 [Tom1l2], Agk, growth associated protein 43 [Gap43]) or downregulated (Rock2, ADP-ribosylation factor-like 1 [Arl1]; Figures 5C–H). Upon comparing the hippocampal proteins (Figures 5H–J) and the transcripts (Figures 5K,L) differentially expressed between BTBR and control B6 mice we found five factors commonly identified in these two tissues (Figure 5M).

### Comparative transcriptomic and proteomic analysis reveals important potential central and peripheral physiological connectivity in the BTBR setting

**Figure 6:**
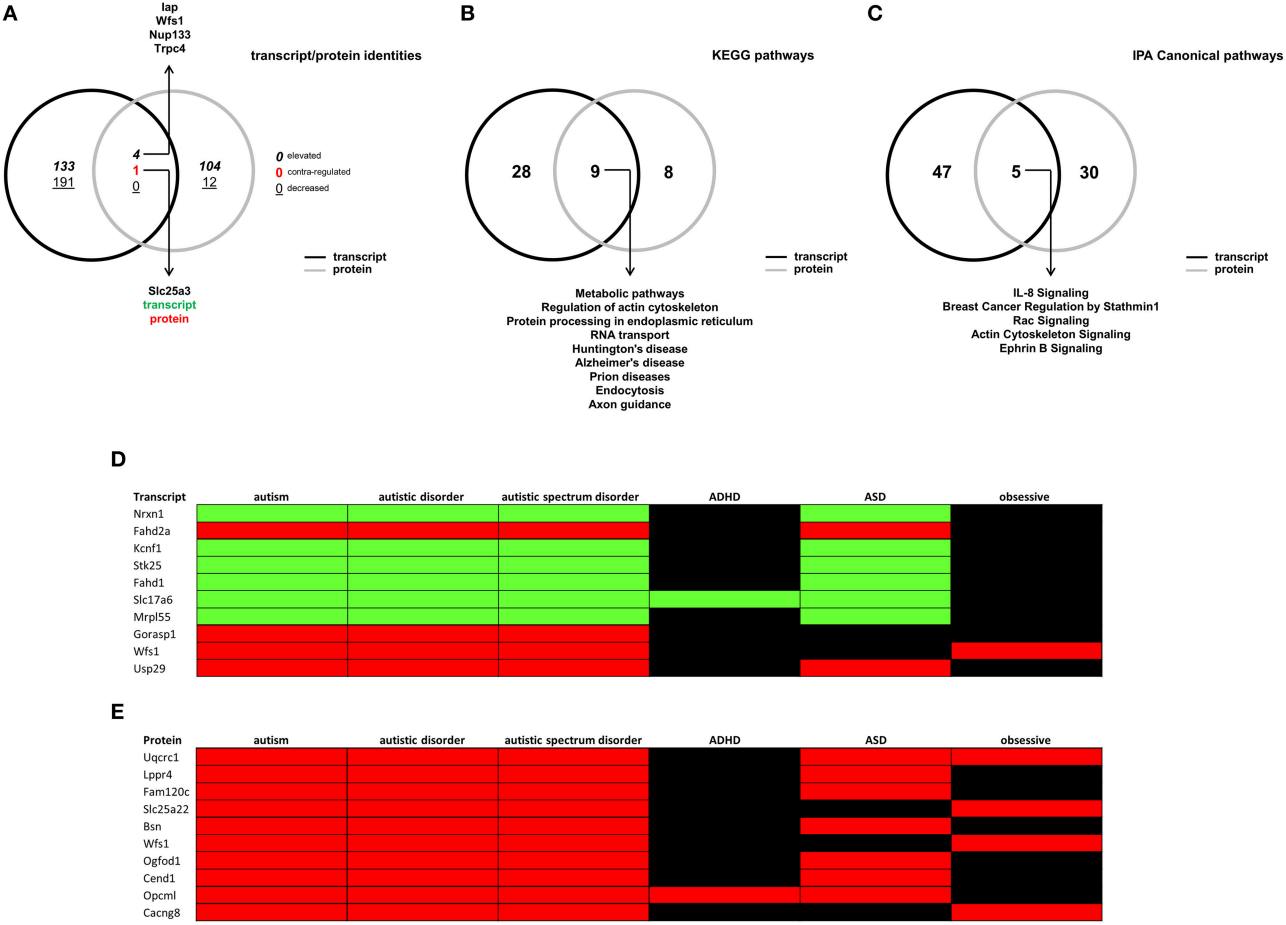
**Latent semantic indexing-based interpretations of hippocampal transcript/protein expression patterns**. **(A)** VennPlex representation of BTBR transcript (black line) and protein (gray line) hippocampal expression profiles (VennPlex numerical set description is as described in Figure 1). Slc25a3 was found to be decreased (green text) in the transcript dataset, while elevated (red text) in the protein dataset. Arrows indicate specific transcripts/proteins of interest to autistic phenotypes. **(B)** VennPlex representation of BTBR transcript (black line) and protein (gray line) hippocampal KEGG pathway annotation profiles. Arrows indicate specific KEGG pathways common to both datasets. **(C)** VennPlex representation of BTBR transcript (black line) and protein (gray line) hippocampal IPA canonical signaling pathway annotation profiles. Arrows indicate specific KEGG pathways common to both datasets. **(D)** Latent Semantic Indexing (LSI) interrogation matrix of significantly-regulated hippocampal transcripts (Top 10 correlating transcripts) extracted using input autism-related search terms (autism—autistic disorder—autistic spectrum disorder—ADHD—ASD—obsessive). **(E)** Latent Semantic Indexing (LSI) interrogation matrix of significantly-regulated hippocampal proteins (Top 10 correlating proteins depicted) extracted using input autism-related search terms (autism—autistic disorder—autistic spectrum disorder—ADHD—ASD—obsessive). Green block = decreased; Red = elevated; black = no correlation identified.

**Figure 7:**
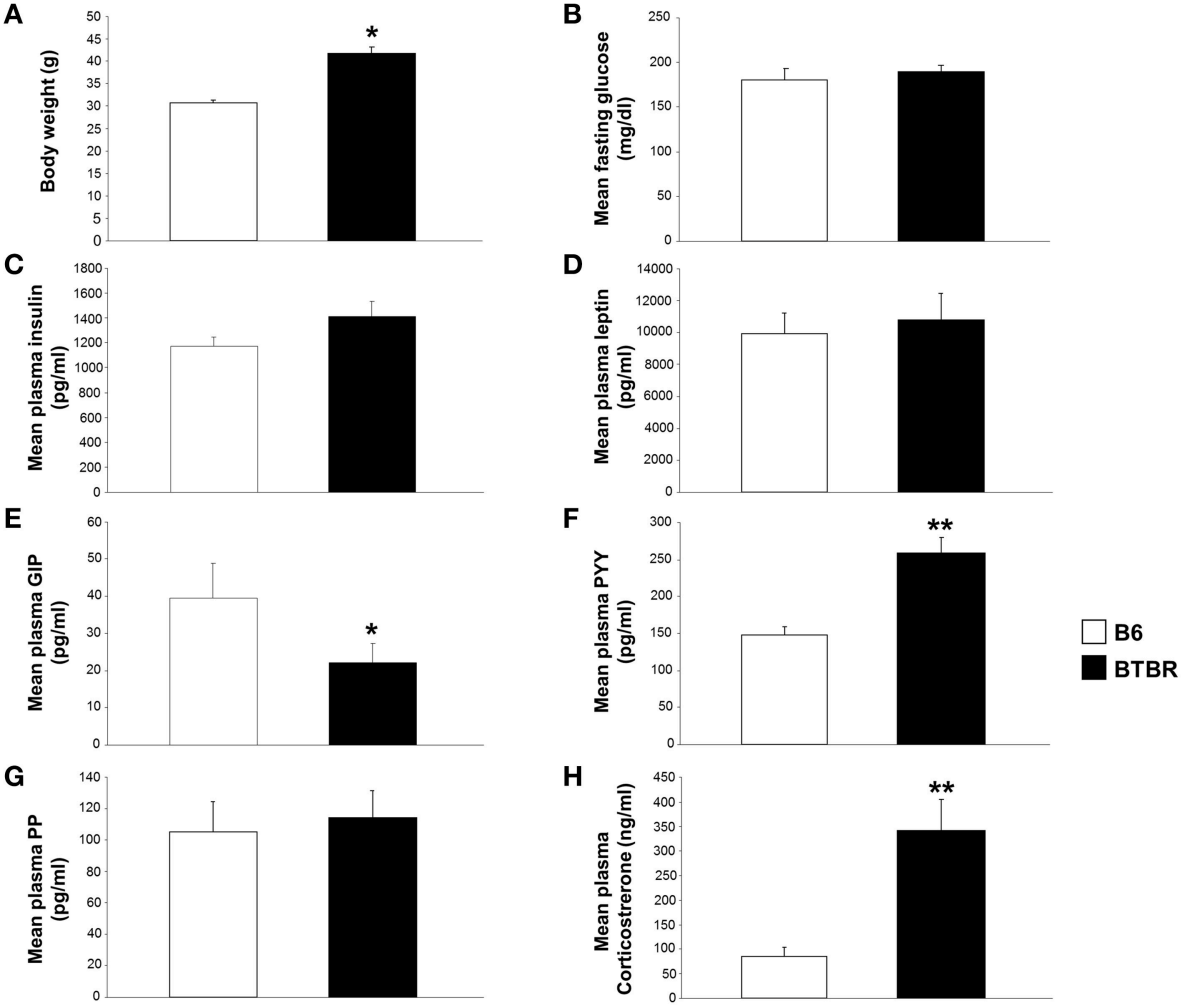
**Significant alterations in body weight, and gut and metabolic hormones in BTBR mice compared to B6 controls**. Significant alterations in body weight, and gut and metabolic hormones in BTBR mice compared to B6 mice. **(A)** Alterations in body weight in BTBR mice compared to B6 controls. Alterations in fasting glucose **(B)**, plasma insulin **(C)**, leptin **(D)**, GIP **(E)**, peptide YY **(F)**, pancreatic polypeptide **(G)**, and corticosterone levels **(H)** in BTBR mice compared to B6 controls. ^*^p ≤ 0.05, ^**^p ≤ 0.01. Histogram white bars represent B6 data and black bars represent BTBR data.

To further investigate the molecular signaling profile of the BTBR hippocampus, we cross-analyzed our two different forms of high-dimensionality data (i.e., transcriptomic and proteomic). When comparing the identities of differentially-regulated factors (Figure 6A), we found that the two datasets were largely distinct aside from four factors: (1) integrin-associated protein form (Iap); (2) Wolframin syndrome 1 (Wfs1); (3) nucleoporin 133 (Nup133); (4) transient receptor potential cation channel, subfamily C, member 4 (Trpc4), which were higher in both datasets in BTBR compared to B6 mice; and (5) Slc25a3, which was lower at the transcript level, but upregulated at the protein level. We also performed both KEGG (Table S12, Figure 6B) and IPA canonical pathway analyses (Table S13, Figure 7C) on differential hippocampal BTBR protein datasets. With respect to the KEGG pathway comparative analysis (Figure 6B), we found that both datasets resulted in the population of nine common signaling pathways clustering in three domains: (1) metabolic (*metabolic pathways, protein processing in endoplasmic reticulum, and RNA transport*); (2) neurodegenerative (*Huntington's disease, Alzheimer's disease, and Prion disease*); and (3) cellular architecture (*regulation of actin cytoskeleton, endocytosis*). Comparing this KEGG analysis with a similar IPA signaling analysis (Figure 6C), we again found that multiple pathways, linked again to cytoskeletal activity (*breast cancer regulation by Stathmin 1, rac signaling, and actin cytoskeleton signaling*) and also receptor platform signaling (*IL-8 signaling and ephrin B signaling*) were significantly populated by both datasets (transcriptomic and proteomic). In order to draw stronger links between our empirical high-dimensionality data and the existing literature corpus related to autism, we then employed GeneIndexer-based latent semantic-indexing (LSI), as previously described (Chadwick et al., 2010), to investigate the relative contribution of individual differentially-altered transcripts and proteins in the hippocampus of BTBR mice to the ASD-like phenotype observed in this murine model. In brief, LSI assesses the strength of correlation between specific transcripts/proteins to “user-defined” interrogation terms by performing textual correlations across large scientific abstract text databases. Using interrogation terms that have been previously associated with the ASD-like phenotype in the BTBR mouse and in human clinical cases of ASD (“*autism*,” “*autistic disorder*,” “*autistic spectrum disorder*,” “*ADHD*,” “*ASD*,” and “*obsessive*”), we sought to detect strong implicit correlations with significantly altered transcripts/proteins present in our empirical hippocampal high-dimensionality datasets. LSI analysis (transcripts—Table S14; proteins—Table S15) revealed that many previously identified autism-related factors were significantly altered in the hippocampus of BTBR mice. The top 10 most strongly autism-correlating empirically-identified transcripts/proteins are depicted in a heatmap format in Figures 6D,E (elevated compared to control = red; decreased compared to control = green). From the transcript analysis, we identified the involvement of neurexin 1 (Yangngam et al., 2014) and Wolframin syndrome 1 (Wfs1; Chakrabarti et al., 2009). From the protein analysis, we again identified Wfs1 as a strong autism-related link, Fam120c (De Wolf et al., 2014), and Bassoon (Bsn; Yoshida et al., 2011). Therefore, the only factor that was strongly implicated in both the transcriptomic and proteomic hippocampal BTBR profiles was Wfs1. The Wfs1 protein has long been implicated in the generation of its titular disease, Wolfram Syndrome, which is an inherited autosomal recessive neurodegenerative disorder (Inoue et al., 1998; Hardy et al., 1999; for review see Rigoli et al., 2011) associated with visual/auditory sensory atrophy and also significant diabetic pathophysiologies. Since many neurodegenerative phenotypes are strongly influenced by metabolic activity (Cai et al., 2012; Janssens et al., 2014; Wang et al., 2014), we investigated whether the consistent alteration of Wfs1 in BTBR mice was associated with any systemic metabolic alterations.

### Significant alterations in body weight and gut and metabolic hormones were found in BTBR mice compared to B6 mice

We assessed differences in body weight and plasma gut and metabolic hormone levels between age/gender-matched BTBR and B6 mice. BTBR mice were found to have significantly increased body mass compared to B6 animals (Figure 7A). Fasting glucose, plasma insulin, leptin, GIP, peptide YY, pancreatic polypeptide, and corticosterone levels were also measured (Figures 7B–H). BTBR mice showed significantly decreased plasma GIP levels and significantly increased plasma peptide YY and plasma corticosterone levels compared to B6 animals (Figures 7E,F,H).

## Discussion

Despite the fact that the behavioral phenotype of the BTBR mouse model of autism has been extensively characterized, the unusual alleles in the BTBR genetic background that contribute to its well-replicated social deficits and repetitive behaviors remain to be determined. In the current study, we have identified a considerable number of transcriptomic and proteomic changes in the BTBR mouse model of ASD compared to B6 mice, which we later validated using iTRAQ, real-time PCR, and western blotting to confirm altered expression levels (Figures 1, 5). Importantly, many of these significantly altered transcripts and proteins, as well as downstream targets of these genes and proteins, have been found to be dysregulated in previous studies of BTBR mice or the human clinical ASD population. Similar to what we observed in the current study, deficits in mature BDNF have been observed in humans with ASD (Sheikh et al., 2010) and a decrease in BDNF mRNA levels has been observed in young BTBR mice (Stephenson et al., 2011); mature BDNF levels were also significantly decreased in the aged BTBR hippocampus and cortex compared with the aged B6 (Jasien et al., 2014).

MAPK3 alterations have been noted in young BTBR mice (Zou et al., 2011; Seese et al., 2014), aged BTBR mice (Jasien et al., 2014), and in the clinical ASD population (Yang et al., 2013). Mutations of multiple genes that have been previously identified in autistic patients were found to be altered in BTBR mice compared to B6 mice, including neurexin 1 (Feng et al., 2006; Szatmari et al., 2007), REEP3 (Castermans et al., 2007), DRD1 (Feng et al., 1999; Hettinger et al., 2008), GABRG2 (Blatt et al., 2001), NOS1AP (Delorme et al., 2010), and NDN (Cai et al., 2008). Furthermore, we have detected significant alterations in transcripts that are downstream targets of effectors known to be dysregulated in the clinical autistic population: Caskin1, which binds to neurexin 1 (Hsueh, 2006), and HOMER31, which binds to SHANK1 and SHANK3 (Abrahams and Geschwind, 2008). SHANK3, in particular, is a very important candidate gene in the ongoing debate of ASD etiology. Known to play a critical role in synaptic function, multiple studies have linked alterations in SHANK3 functionality within the ASD phenotype in a human clinical population (Durand et al., 2007; Bourgeron, 2009). Furthermore, SHANK3 heterozygous mice show deficits in synaptic function and plasticity, in addition to demonstrating reduced reciprocal social interactions reminiscent of the ASD phenotype (Bozdagi et al., 2010; Yang et al., 2012), while SHANK1 knockout mice show reductions in ultrasonic vocalizations and scent marking behavior (Wöhr et al., 2011). Moreover, a number of the genes we found to be significantly altered in the BTBR mouse, while not directly linked to ASD, have been implicated in diseases with a similar trajectory: TSC2 (tuberous sclerosis; Persico and Bourgeron, 2006), PAK1 (FXS; Hayashi et al., 2007), Nr4a2 (Parkinson's, schizophrenia, and manic depression; Lybaek et al., 2009), intellectual disability (Smith et al., 2005), Alzheimer's disease (Pardo and van Duijn, 2005), Slc6a13 (anxiety disorders; Saus et al., 2010), and Adra2c (ADHD; Cho et al., 2008). Similarly, there have been instances in which we have identified genes from the same gene family as those that have previously been linked to ASD: Slc25a3 (Ramoz et al., 2004) and CADM3 (Fujita et al., 2010). Slc25a3 was also found to be altered in the aged BTBR mice compared to the B6 mice (Jasien et al., 2014). With future, more extensive studies employing greater mice populations there will be the potential to uncover potential associations between transcript expression profiles and genomic idiosyncracies within the BTBRT+Itprtf/J model (Jones-Davis et al., 2013).

While it is important to identify the individual genes and proteins dysregulated in both animal models of ASD and in the human clinical population, it is perhaps more worthwhile, given the interconnected network nature of the central nervous system, to examine the functional relationships between multiple coherently-regulated genes and proteins. As a result, we employed IPA canonical signaling pathway, KEGG signaling pathway, and *Textrous!* natural language processing-based informatics analyses to identify significantly altered functional groups and pathways in BTBR mice compared to B6 animals (Figures 2–4, 6). Importantly, many of the significantly altered functional groups and pathways we have identified here have also been indicated in previous studies and, furthermore, have been suggested as a possible etiological mechanism of ASD, including “*axon guidance”* (Suda et al., 2011), “*neurogenesis*” (McCaffery and Deutsch, 2005), and “*regulation of actin cytoskeleton”* (Durand et al., 2012). Our previous informatics analyses of aged BTBR hippocampi demonstrated a strong bias toward degenerative phenotypes associated with calcium management factors and metabolic instabilities (Jasien et al., 2014).

We believe that the rational application of combinatorial informatic approaches are capable of detecting signaling pathways associated with the ASD-like phenotype, in addition to individual transcriptomic and proteomic alterations that contribute to the ASD-like phenotype generation. Similarly the altered metabolic phenotype we observed in the BTBR mice, potentially associated with Wfs1 expression modulation, have been corroborated in the past, with BTBR mice having increased insulin resistance and higher fasting insulin levels (Rabaglia et al., 2005; Flowers et al., 2007). Increased corticosterone levels have also been found in BTBR mice compared to B6 controls (Frye and Llaneza, 2010; Silverman et al., 2010). Notably, Wolfram Syndrome, also known as DIDMOAD (diabetes insipidus, insulin-deficient diabetes mellitus, optic atrophy, and deafness), has been strongly associated with significant disruptions in endoplasmic reticulum functionality (Figure 7B, “*protein processing in endoplasmic reticulum”*), membrane trafficking (Figure 7B, “*endocytosis”*), and calcium homeostasis (Krey and Dolmetsch, 2007). In using wide-scale bioinformatics techniques on an animal model in which the ASD-like phenotype has already been extensively characterized, we aimed to identify the genes and proteins responsible for the presentation of ASD-like phenotypes in this inbred strain mouse model. Given the similarity in presentation of symptoms in BTBR mice compared to the clinical ASD population, we hoped to discover similar parallels between relevant genes and proteins that could be extrapolated to a human clinical population. We hoped that these approaches would lead to the identification of previously undiscovered potential causes of ASD. Given the many links to current clinical literature, we believe that bioinformatics offers specific advantages into the etiology of ASD that studying the pure behavioral phenotype of the BTBR mouse, or genetic mutations in specific ASD families, in and of themselves do not. The first advantage bioinformatics offers is simply the sheer wealth of data gained from genome-wide studies, which allows for a more complete analysis. Second, using unbiased bioinformatics approaches can lead to the discovery of previously unidentified connections, such as our discovery that Caskin1 is significantly altered in BTBR mice. Uncovering previously unknown transcriptomic or proteomic changes associated with the ASD-like phenotype has the potential to lead to novel therapeutic targets for the treatment of ASD. Several groups that have already discovered the merit of wide-scale transcriptomic and proteomic studies have produced complex interactomes that offer a more complete disease profile of ASD (Sakai et al., 2011; Voineagu et al., 2011). In order to ultimately succeed in characterizing the etiology of ASD and subsequently develop effective and targeted therapeutics for these disorders, we believe that bioinformatics and similar wide-scale approaches to studying disease etiology are indispensable avenues of study. Finally, due to the fact that our quantitative transcriptomic and proteomic analyses were strongly correlated with previous literature, we believe it is possible to predict complex behavioral phenotypes from bioinformatics analyses alone. To this effect, we hope that our study and other similar examinations will offer new directions for uncovering the etiology of ASD and its potential therapies.

## Funding

This work was carried out with the support of the Intramural Research Programs of the National Institute on Aging (NIH-AG000325-02) and the National Institute of Mental Health at the National Institutes of Health.

### Conflict of interest statement

The authors declare that the research was conducted in the absence of any commercial or financial relationships that could be construed as a potential conflict of interest.

## Supplementary material

Figure S1 **Principal component analysis of murine transcriptomic data**. (A) The three-dimensional Principal component analysis (PCA: using SAS JMP®) chart indicates the relative spatial positions of the transcriptomic data (expressed as z-scores) obtained from the cortex and hippocampus of control B6 mice (*n* = 3, B6-1, B6-2, and B6-3) and their BTBR counterparts (*n* = 3, BTBR-1, BTBR-2, and BTBR-3). The specific PCs (x, y, and z: %) for the overall analysis are indicated in to the right of three-dimensional plot. Essentially, a strong genotype clustering is observed between B6 controls and BTBR, as well as a considerable spatial separation between cortical and hippocampal tissues in each murine genotype group.

## Abbreviations

Agk: acylglycerol kinase
ASD: autism spectrum disorder
B6: control mice
BTBR: black and tan, brachyury
GIP: gastric inhibitory polypeptide
IPA: ingenuity pathway analysis
iTRAQ: isobaric tags for relative and absolute quantification
KEGG: Kyoto Encyclopedia of Genes and Genomes
LSI: Latent Semantic Indexing
PCR: polymerase chain reaction
Rock2: Rho-associated coiled-coil containing protein kinase 2
Wfs 1: Wolframin syndrome 1.
