# Supplementary figures and images for: Hippocampal Transcriptomic and Proteomic Alterations in the BTBR Mouse Model of Autism Spectrum Disorder
Source: Front Physiol. 2015 Nov 24;6:324. doi: 10.3389/fphys.2015.00324 (PMC4656818; doi:10.3389/fphys.2015.00324)

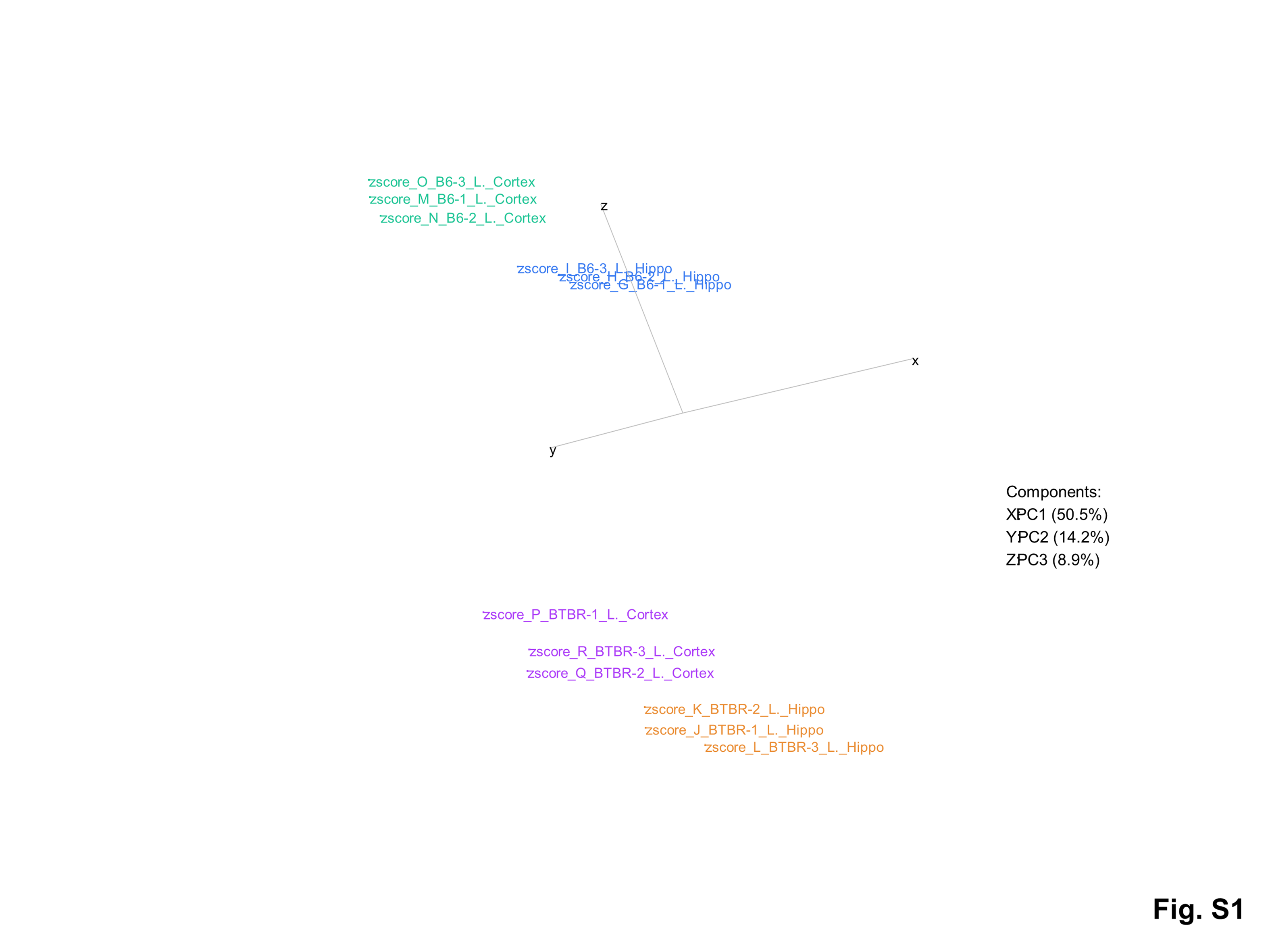

Supplement: Figure S1 — Principal component analysis of murine transcriptomic data. (A) The three-dimensional Principal component analysis (PCA: using SAS JMP®) chart indicates the relative spatial positions of the transcriptomic data (expressed as z-scores) obtained from the cortex and hippocampus of control B6 mice (n = 3, B6-1, B6-2, and B6-3) and their BTBR counterparts (n = 3, BTBR-1, BTBR-2, and BTBR-3). The specific PCs (x, y, and z: %) for the overall analysis are indicated in to the right of three-dimensional plot. Essentially, a strong genotype clustering is observed between B6 controls and BTBR, as well as a considerable spatial separation between cortical and hippocampal tissues in each murine genotype group. [file Image1.TIF]
