# Supplementary material for: Hippocampal Transcriptomic and Proteomic Alterations in the BTBR Mouse Model of Autism Spectrum Disorder
Source: Front Physiol. 2015 Nov 24;6:324. doi: 10.3389/fphys.2015.00324 (PMC4656818; doi:10.3389/fphys.2015.00324)
Supplement: Supplementary file 2 [file Table1.DOCX]

**Table S1. Significant differential mRNA transcript expression between BTBR and B6 hippocampus**. The table indicates the significant and differentially-expressed transcripts between BTBR and B6 mouse hippocampus. For each significantly-regulated (p<0.05) transcript the Official Gene Symbol, textual transcript Definition and expression Z ratio (BTBR versus B6) is given.

| **Gene Symbol** | **Definition** | **Z ratio** |
| --- | --- | --- |
| Slc7a14 | solute carrier family 7 (cationic amino acid transporter, y+ system), member 14 (Slc7a14) | 12.11 |
| Serpina3n | serine (or cysteine) peptidase inhibitor, clade A, member 3N (Serpina3n) | 7.87 |
| Lypd1 | Ly6/Plaur domain containing 1 (Lypd1) | 6.17 |
| Lcmt1 | leucine carboxyl methyltransferase 1 (Lcmt1) | 5.25 |
| Cap1 | CAP, adenylate cyclase-associated protein 1 (yeast) (Cap1) | 4.97 |
| Mrps10 | mitochondrial ribosomal protein S10 (Mrps10), nuclear gene encoding mitochondrial protein | 4.42 |
| Grlf1 | glucocorticoid receptor DNA binding factor 1 (Grlf1) | 4.24 |
| LOC677317 | Mod1 protein, transcript variant 4 (LOC677317) | 4.16 |
| Extl1 | exostoses (multiple)-like 1 (Extl1) | 4.1 |
| Lpl | lipoprotein lipase (Lpl) | 3.92 |
| Kras | v-Ki-ras2 Kirsten rat sarcoma viral oncogene homolog (Kras) | 3.87 |
| 2310002B06Rik | RIKEN cDNA 2310002B06 gene (2310002B06Rik) | 3.76 |
| Usp29 | ubiquitin specific peptidase 29 (Usp29) | 3.7 |
| Centg1 | centaurin, gamma 1 (Centg1) | 3.54 |
| Adi1 | acireductone dioxygenase 1 (Adi1) | 3.4 |
| Ndufb10 | NADH dehydrogenase (ubiquinone) 1 beta subcomplex, 10 (Ndufb10) | 3.39 |
| Iqgap1 | IQ motif containing GTPase activating protein 1 (Iqgap1) | 3.38 |
| Eno1 | enolase 1, alpha non-neuron (Eno1) | 3.25 |
| Nubp2 | nucleotide binding protein 2 (Nubp2) | 3.17 |
| LOC100046744 | Serine/arginine repetitive matrix protein 2 (LOC100046744) | 3.16 |
| Snx30 | sorting nexin family member 30 (Snx30) | 3.16 |
| Rtn4 | reticulon 4 (RTn4) | 3.08 |
| Iap | integrin associated protein (Iap) | 3.08 |
| Scg5 | secretogranin V (Scg5) | 2.94 |
| Sorl1 | sortilin-related receptor, LDLR class A repeats-containing (Sorl1) | 2.89 |
| Hmgn2 | high mobility group nucleosomal binding domain 2 (Hmgn2) | 2.79 |
| 4933439C20Rik | RIKEN cDNA 4933439C20 gene (4933439C20Rik) | 2.75 |
| Cox18 | COX18 cytochrome c oxidase assembly homolog (S. cerevisiae) (Cox18) | 2.6 |
| Zfp94 | zinc finger protein 94 (Zfp94) | 2.53 |
| Blvrb | biliverdin reductase B (flavin reductase (NADPH)) (Blvrb) | 2.53 |
| Coq9 | coenzyme Q9 homolog (yeast) (Coq9) | 2.45 |
| Nicn1 | nicolin 1 (Nicn1) | 2.44 |
| Wbp11 | WW domain binding protein 11 (Wbp11) | 2.36 |
| Sox21 | SRY-box containing gene 21 (Sox21) | 2.41 |
| Bbs7 | Bardet-Biedl syndrome 7 (Bbs7) | 2.38 |
| Ntn2l | netrin 2-like (chicken) (Ntn2l) | 2.31 |
| Uba5 | ubiquitin-like modifier activating enzyme 5 (Uba5) | 2.31 |
| Ndn | necdin (Ndn) | 2.33 |
| Lrrc49 | leucine rich repeat containing 49 (Lrrc49) | 2.3 |
| Ap2s1 | adaptor-related protein complex 2, sigma 1 subunit (Ap2s1) | 2.24 |
| Hn1 | hematological and neurological expressed sequence 1 (Hn1) | 2.22 |
| Dbnl | drebrin-like (Dbnl) | 2.21 |
| Gins4 | GINS complex subunit 4 (Sld5 homolog) (Gins4) | 2.2 |
| Olfml1 | olfactomedin-like 1 (Olfml1) | 2.2 |
| Fbxo34 | F-box protein 34 (Fbxo34) | 2.18 |
| Kpnb1 | karyopherin (importin) beta 1 (Kpnb1) | 2.09 |
| Mrps10 | mitochondrial ribosomal protein S10 (Mrps10), nuclear gene encoding mitochondrial protein | 2.09 |
| Asah3l | N-acylsphingosine amidohydrolase 3-like (Asah3l) | 2.07 |
| Vdac2 | voltage-dependent anion channel 2 (Vdac2) | 2.02 |
| 1190002H23Rik | RIKEN cDNA 1190002H23 gene (1190002H23Rik) | 2 |
| Igsf1 | immunoglobulin superfamily, member 1 (Igsf1), transcript variant 4 | 1.99 |
| Npcd | neuronal pentraxin with chromo domain (Npcd), transcript variant 2 | 1.95 |
| Chd8 | chromodomain helicase DNA binding protein 8 (Chd8) | 1.98 |
| Rerg | RAS-like, estrogen-regulated, growth-inhibitor (Rerg) | 1.98 |
| Pole4 | polymerase (DNA-directed), epsilon 4 (p12 subunit) (Pole4) | 1.94 |
| Clec16a | C-type lectin domain family 16, member A (Clec16a) | 1.93 |
| Ptpn1 | protein tyrosine phosphatase, non-receptor type 1 (Ptpn1) | 1.84 |
| Foxred1 | FAD-dependent oxidoreductase domain containing 1 (Foxred1) | 1.94 |
| Hsp90ab1 | heat shock protein 90kDa alpha (cytosolic), class B member 1 (Hsp90ab1) | 1.96 |
| Lrrc49 | leucine rich repeat containing 49 (Lrrc49) | 1.95 |
| Igsf1 | immunoglobulin superfamily, member 1 (Igsf1), transcript variant 4 | 1.88 |
| Col16a1 | collagen, type XVI, alpha 1 (Col16a1) | 1.89 |
| Cadm3 | cell adhesion molecule 3 (Cadm3) | 1.87 |
| Ptprs | protein tyrosine phosphatase, receptor type, S (Ptprs) | 1.84 |
| Atp9a | ATPase, class II, type 9A (Atp9a) | 1.84 |
| Drbp1 | RNA binding motif protein 45 (Drbp1) | 1.88 |
| Pld3 | phospholipase D family, member 3 (Pld3) | 1.87 |
| Nudc | nuclear distribution gene C homolog (Aspergillus) (Nudc) | 1.87 |
| Gria1 | glutamate receptor, ionotropic, AMPA1 (alpha 1) (Gria1) | 1.88 |
| Spock1 | sparc/osteonectin, cwcv and kazal-like domains proteoglycan 1 (Spock1) | 1.85 |
| Zfp30 | zinc finger protein 30 (Zfp30) | 1.83 |
| Ecsit | ECSIT homolog (Drosophila) (Ecsit) | 1.83 |
| BC008163 | cDNA sequence BC008163 (BC008163) | 1.84 |
| Irf2bp1 | interferon regulatory factor 2 binding protein 1 (Irf2bp1) | 1.8 |
| Lpin2 | lipin 2 (Lpin2) | 1.81 |
| Rreb1 | ras responsive element binding protein 1 (Rreb1), transcript variant 1 | 1.82 |
| Tmod2 | tropomodulin 2 (Tmod2), transcript variant 1 | 1.83 |
| Faim2 | Fas apoptotic inhibitory molecule 2 (Faim2), transcript variant 2 | 1.77 |
| 4930511J11Rik | RIKEN cDNA 4930511J11 gene (4930511J11Rik) | 1.75 |
| Dcp1b | DCP1 decapping enzyme homolog b (S. cerevisiae) (Dcp1b) | 1.77 |
| Snapc3 | small nuclear RNA activating complex, polypeptide 3 (Snapc3) | 1.75 |
| BC057371 | cDNA sequence BC057371 (BC057371) | 1.75 |
| Serpinf1 | serine (or cysteine) peptidase inhibitor, clade F, member 1 (Serpinf1) | 1.73 |
| Guk1 | guanylate kinase 1 (Guk1) | 1.76 |
| 6430517E21Rik | RIKEN cDNA 6430517E21 gene (6430517E21Rik) | 1.74 |
| Tomm70a | translocase of outer mitochondrial membrane 70 homolog A (yeast) (Tomm70a) | 1.69 |
| Anxa5 | annexin A5 (Anxa5) | 1.7 |
| Gfer | growth factor, erv1 (S. cerevisiae)-like (augmenter of liver regeneration) (Gfer) | 1.72 |
| Ahdc1 | AT hook, DNA binding motif, containing 1 (Ahdc1) | 1.69 |
| 3110035E14Rik | RIKEN cDNA 3110035E14 gene (3110035E14Rik) | 1.73 |
| Shd | src homology 2 domain-containing transforming protein D (Shd) | 1.7 |
| Matk | megakaryocyte-associated tyrosine kinase (Matk) | 1.74 |
| Gria1 | glutamate receptor, ionotropic, AMPA1 (alpha 1) (Gria1) | 1.75 |
| Mrpl3 | mitochondrial ribosomal protein L3 (Mrpl3), nuclear gene encoding mitochondrial protein | 1.7 |
| Fbxo6 | F-box protein 6 (Fbxo6) | 1.71 |
| Sae1 | SUMO1 activating enzyme subunit 1 (Sae1) | 1.71 |
| Pgam2 | phosphoglycerate mutase 2 (Pgam2) | 1.68 |
| Cacng5 | calcium channel, voltage-dependent, gamma subunit 5 (Cacng5) | 1.63 |
| Cxx1a | CAAX box 1 homolog A (human) (Cxx1a) | 1.61 |
| Fahd2a | fumarylacetoacetate hydrolase domain containing 2A (Fahd2a) | 1.66 |
| Tubb2b | tubulin, beta 2b (Tubb2b) | 1.69 |
| Sae1 | SUMO1 activating enzyme subunit 1 (Sae1) | 1.66 |
| BC026590 | cDNA sequence BC026590 (BC026590) | 1.66 |
| Tfrc | transferrin receptor (Tfrc) | 1.67 |
| 8430415E04Rik | RIKEN cDNA 8430415E04 gene (8430415E04Rik) | 1.58 |
| Rreb1 | ras responsive element binding protein 1 (Rreb1), transcript variant 1 | 1.66 |
| BC057371 | cDNA sequence BC057371 (BC057371) | 1.62 |
| Adra2c | adrenergic receptor, alpha 2c (Adra2c) | 1.63 |
| Centg1 | centaurin, gamma 1 (Centg1) | 1.6 |
| Actr3b | ARP3 actin-related protein 3 homolog B (yeast) (Actr3b) | 1.57 |
| Atp6v0d1 | ATPase, H+ transporting, lysosomal V0 subunit D1 (Atp6v0d1) | 1.61 |
| Prr13 | proline rich 13 (Prr13) | 1.59 |
| Hnrpdl | heterogeneous nuclear ribonucleoprotein D-like (Hnrpdl) | 1.64 |
| Wfs1 | Wolfram syndrome 1 homolog (human) (Wfs1) | 1.66 |
| Nup133 | nucleoporin 133 (Nup133) | 1.6 |
| Spire1 | spire homolog 1 (Drosophila) (Spire1), transcript variant 2 | 1.59 |
| Nudc | nuclear distribution gene C homolog (Aspergillus) (Nudc) | 1.57 |
| Camk1d | calcium/calmodulin-dependent protein kinase ID (Camk1d) | 1.56 |
| Trappc2l | trafficking protein particle complex 2-like (Trappc2l) | 1.59 |
| BC057552 | cDNA sequence BC057552 (BC057552) | 1.58 |
| Gria1 | glutamate receptor, ionotropic, AMPA1 (alpha 1) (Gria1) | 1.59 |
| B9d1 | B9 protein domain 1 (B9d1) | 1.58 |
| St8sia5 | ST8 alpha-N-acetyl-neuraminide alpha-2,8-sialyltransferase 5 (St8sia5), transcript variant 2 | 1.56 |
| Napa | N-ethylmaleimide sensitive fusion protein attachment protein alpha (Napa) | 1.58 |
| Nt5c | 5',3'-nucleotidase, cytosolic (Nt5c) | 1.55 |
| Ppil3 | peptidylprolyl isomerase (cyclophilin)-like 3 (Ppil3), transcript variant 1 | 1.56 |
| Zdhhc4 | zinc finger, DHHC domain containing 4 (Zdhhc4) | 1.55 |
| Zfp612 | zinc finger protein 612 (Zfp612) | 1.53 |
| Gorasp1 | golgi reassembly stacking protein 1 (Gorasp1) | 1.54 |
| Prkd2 | protein kinase D2 (Prkd2) | 1.55 |
| Vps33b | vacuolar protein sorting 33B (yeast) (Vps33b) | 1.51 |
| Rps2 | ribosomal protein S2 (Rps2) | 1.55 |
| Trpc4 | transient receptor potential cation channel, subfamily C, member 4 (Trpc4) | 1.5 |
| Farsb | phenylalanyl-tRNA synthetase, beta subunit (Farsb) | 1.57 |
| Rb1 | retinoblastoma 1 (Rb1) | 1.51 |
| Mrpl33 | mitochondrial ribosomal protein L33 (Mrpl33), nuclear gene encoding mitochondrial protein | 1.51 |
| Ndrl | N-myc downstream regulated gene 1 (Ndrl) | -1.5 |
| Tspyl3 | TSPY-like 3 (Tspyl3) | -1.51 |
| Hist1h2bj | histone cluster 1, H2bj (Hist1h2bj) | -1.51 |
| Rab6 | RAB6, member RAS oncogene family (Rab6) | -1.52 |
| Sesn1 | sestrin 1 (Sesn1) | -1.51 |
| S100a13 | S100 calcium binding protein A13 (S100a13) | -1.53 |
| Tex261 | testis expressed gene 261 (Tex261) | -1.58 |
| Snrpd1 | small nuclear ribonucleoprotein D1 (Snrpd1) | -1.51 |
| Nrxn1 | neurexin I (Nrxn1) | -1.53 |
| Fahd1 | fumarylacetoacetate hydrolase domain containing 1 (Fahd1), nuclear gene encoding mitochondrial protein | -1.6 |
| Fbxo34 | F-box protein 34 (Fbxo34) | -1.53 |
| Myadm | myeloid-associated differentiation marker (Myadm) | -1.54 |
| Mras | muscle and microspikes RAS (Mras) | -1.59 |
| Tbcb | tubulin folding cofactor B (Tbcb) | -1.55 |
| Gtpbp1 | GTP binding protein 1 (Gtpbp1) | -1.55 |
| Chmp4b | chromatin modifying protein 4B (Chmp4b) | -1.55 |
| Rpo2tc1 | SUB1 homolog (S. cerevisiae) (Rpo2tc1) | -1.52 |
| Stac2 | SH3 and cysteine rich domain 2 (Stac2) | -1.56 |
| Nos1ap | nitric oxide synthase 1 (neuronal) adaptor protein (Nos1ap) | -1.52 |
| Fmn2 | formin 2 (Fmn2) | -1.58 |
| Uhrf1bp1l | UHRF1 (ICBP90) binding protein 1-like (Uhrf1bp1l) | -1.58 |
| Arl3 | ADP-ribosylation factor-like 3 (Arl3) | -1.63 |
| Bex4 | brain expressed gene 4 (Bex4) | -1.6 |
| Myh9 | myosin, heavy polypeptide 9, non-muscle (Myh9) | -1.6 |
| Nars | asparaginyl-tRNA synthetase (Nars) | -1.65 |
| Igsf4a | cell adhesion molecule 1 (Igsf4a) | -1.58 |
| Abhd4 | abhydrolase domain containing 4 (Abhd4) | -1.63 |
| 4933407N01Rik | RIKEN cDNA 4933407N01Rik gene (4933407N01Rik) | -1.63 |
| 1810020D17Rik | RIKEN cDNA 1810020D17 gene (1810020D17Rik) | -1.61 |
| Ddhd1 | DDHD domain containing 1 (Ddhd1) | -1.64 |
| Hsd11b1 | hydroxysteroid 11-beta dehydrogenase 1 (Hsd11b1), transcript variant 1 | -1.61 |
| Nr4a2 | nuclear receptor subfamily 4, group A, member 2 (Nr4a2) | -1.65 |
| Mrpl9 | mitochondrial ribosomal protein L9 (Mrpl9), nuclear gene encoding mitochondrial protein | -1.64 |
| Aprt | adenine phosphoribosyl transferase (Aprt) | -1.65 |
| Tsc22d3 | TSC22 domain family 3 (Tsc22d3), transcript variant 1 | -1.63 |
| Cx3cr1 | chemokine (C-X3-C motif) receptor 1 (Cx3cr1) | -1.66 |
| Ddit4l | DNA-damage-inducible transcript 4-like (Ddit4l) | -1.55 |
| Fmn2 | formin 2 (Fmn2) | -1.65 |
| Zcchc17 | zinc finger, CCHC domain containing 17 (Zcchc17) | -1.68 |
| Ramp2 | receptor (calcitonin) activity modifying protein 2 (Ramp2) | -1.67 |
| BC025076 | membrane magnesium transporter 2 (Mmgt2) | -1.71 |
| Bdnf | brain derived neurotrophic factor (Bdnf), transcript variant 3 | -1.73 |
| Rnf11 | ring finger protein 11 (Rnf11) | -1.72 |
| Ddx24 | DEAD (Asp-Glu-Ala-Asp) box polypeptide 24 (Ddx24) | -1.69 |
| Coro2b | coronin, actin binding protein, 2B (Coro2b) | -1.76 |
| Mgll | monoglyceride lipase (Mgll) | -1.77 |
| Ppapdc2 | phosphatidic acid phosphatase type 2 domain containing 2 (Ppapdc2) | -1.76 |
| Dbi | diazepam binding inhibitor (Dbi), transcript variant 2 | -1.71 |
| Purb | purine rich element binding protein B (Purb) | -1.76 |
| Snrpd1 | small nuclear ribonucleoprotein D1 (Snrpd1) | -1.74 |
| Chn2 | chimerin (chimaerin) 2 (Chn2) | -1.79 |
| Armc8 | armadillo repeat containing 8 (Armc8) | -1.8 |
| Ppm1m | protein phosphatase 1M (Ppm1m), transcript variant 1 | -1.8 |
| Dbi | diazepam binding inhibitor (Dbi), transcript variant 2 | -1.83 |
| Psenen | presenilin enhancer 2 homolog (C. elegans) (Psenen) | -1.8 |
| Cetn4 | centrin 4 (Cetn4) | -1.83 |
| Fscn1 | fascin homolog 1, actin bundling protein (Strongylocentrotus purpuratus) (Fscn1) | -1.89 |
| Ly6a | lymphocyte antigen 6 complex, locus A (Ly6a) | -1.89 |
| Ttc17 | tetratricopeptide repeat domain 17 (Ttc17) | -1.84 |
| B2m | beta-2 microglobulin (B2m) | -1.85 |
| Lgals1 | lectin, galactose binding, soluble 1 (Lgals1) | -1.85 |
| Hsd11b1 | hydroxysteroid 11-beta dehydrogenase 1 (Hsd11b1) | -1.9 |
| Ints7 | integrator complex subunit 7 (Ints7) | -1.91 |
| rp9 | retinitis pigmentosa 9 (human) (rp9) | -1.91 |
| Cnot4 | CCR4-NOT transcription complex, subunit 4 (Cnot4) | -1.96 |
| Gnptab | N-acetylglucosamine-1-phosphate transferase, alpha and beta subunits (Gnptab) | -1.98 |
| 1810030N24Rik | RIKEN cDNA 1810030N24 gene (1810030N24Rik) | -1.98 |
| Ccnd1 | cyclin D1 (Ccnd1) | -2 |
| Meis2 | Meis homeobox 2 (Meis2) | -2.11 |
| Hist2h2ac | histone cluster 2, H2ac (Hist2h2ac) | -2.04 |
| Gtpbp4 | GTP binding protein 4 (Gtpbp4) | -2.05 |
| Clcn7 | chloride channel 7 (Clcn7) | -2.04 |
| Stk25 | serine/threonine kinase 25 (yeast) (Stk25) | -2.08 |
| Amigo2 | adhesion molecule with Ig like domain 2 (Amigo2) | -2.1 |
| Kcnip3 | Kv channel interacting protein 3, calsenilin (Kcnip3) | -2.18 |
| Zc3h13 | zinc finger CCCH type containing 13 (Zc3h13) | -2.14 |
| LOC100046741 | red-1 (LOC100046741) | -2.02 |
| LOC100040573 | putative transcription factor ZNF131, transcript variant 1 (LOC100040573) | -2.12 |
| Ndrg3 | N-myc downstream regulated gene 3 (Ndrg3) | -2.12 |
| Rnf166 | ring finger protein 166 (Rnf166) | -2.17 |
| Mrpl48 | mitochondrial ribosomal protein L48 (Mrpl48), nuclear gene encoding mitochondrial protein | -2.16 |
| Il33 | interleukin 33 (Il33) | -2.2 |
| Tsc2 | tuberous sclerosis 2 (Tsc2), transcript variant 2 | -2.14 |
| Rnpep | arginyl aminopeptidase (aminopeptidase B) (Rnpep) | -2.2 |
| Entpd4 | ectonucleoside triphosphate diphosphohydrolase 4 (Entpd4) | -2.17 |
| Cib2 | calcium and integrin binding family member 2 (Cib2) | -2.19 |
| Capns1 | calpain, small subunit 1 (Capns1) | -2.31 |
| Mrpl48 | mitochondrial ribosomal protein L48 (Mrpl48), transcript variant 1 | -2.19 |
| 1700123O20Rik | RIKEN cDNA 1700123O20 gene (1700123O20Rik) | -2.19 |
| Mrps23 | mitochondrial ribosomal protein S23 (Mrps23), nuclear gene encoding mitochondrial protein | -2.21 |
| Tex261 | testis expressed gene 261 (Tex261) | -2.23 |
| Zfp131 | zinc finger protein 131 (Zfp131) | -2.21 |
| Rpl29 | ribosomal protein L29 (Rpl29) | -2.24 |
| Stt3b | STT3, subunit of the oligosaccharyltransferase complex, homolog B (S. cerevisiae) (Stt3b) | -2.21 |
| Dmwd | dystrophia myotonica-containing WD repeat motif (Dmwd) | -2.28 |
| Caskin1 | CASK interacting protein 1 (Caskin1) | -2.37 |
| Drctnnb1a | family with sequence similarity 126, member A (Drctnnb1a) | -2.31 |
| Hdhd2 | haloacid dehalogenase-like hydrolase domain containing 2 (Hdhd2), transcript variant 1 | -2.4 |
| Slc17a6 | solute carrier family 17 (sodium-dependent inorganic phosphate cotransporter), member 6 (Slc17a6) | -2.31 |
| Cib2 | calcium and integrin binding family member 2 (Cib2) | -2.38 |
| 4930570C03Rik | RIKEN cDNA 4930570C03 gene (4930570C03Rik) | -2.36 |
| 1700025G04Rik | RIKEN cDNA 1700025G04 gene (1700025G04Rik) | -2.43 |
| B2m | beta-2 microglobulin (B2m) | -2.42 |
| Trf | transferrin (Trf) | -2.51 |
| Atf7ip | activating transcription factor 7 interacting protein (Atf7ip) | -2.44 |
| 2610020C11Rik | RIKEN cDNA 2610020C11Rik gene (2610020C11Rik) | -2.44 |
| 6330403K07Rik | RIKEN cDNA 6330403K07 gene (6330403K07Rik) | -2.5 |
| Reep3 | receptor accessory protein 3 (Reep3) | -2.52 |
| Pon2 | paraoxonase 2 (Pon2) | -2.48 |
| Pfdn5 | prefoldin 5 (Pfdn5) | -2.55 |
| Igsf3 | immunoglobulin superfamily, member 3 (Igsf3) | -2.56 |
| Pfdn5 | prefoldin 5 (Pfdn5) | -2.59 |
| Riok1 | RIO kinase 1 (yeast) (Riok1) | -2.64 |
| AI316807 | expressed sequence AI316807 (AI316807) | -2.65 |
| Pop4 | processing of precursor 4, ribonuclease P/MRP family, (S. cerevisiae) (Pop4) | -2.68 |
| Med23 | mediator complex subunit 23 (Med23) | -2.7 |
| Gtf3c1 | general transcription factor III C 1 (Gtf3c1) | -2.71 |
| Crym | crystallin, mu (Crym) | -2.68 |
| C1qc | complement component 1, q subcomponent, C chain (C1qc) | -2.77 |
| Atp5g2 | ATP synthase, H+ transporting, mitochondrial F0 complex, subunit c (subunit 9), isoform 2 (Atp5g2) | -2.72 |
| Ube2m | ubiquitin-conjugating enzyme E2M (UBC12 homolog, yeast) (Ube2m) | -2.86 |
| LOC100048331 | DnaJ (Hsp40) homolog, subfamily A, member 4 (LOC100048331) | -2.77 |
| Sparc | secreted acidic cysteine rich glycoprotein (Sparc) | -2.81 |
| C1qb | complement component 1, q subcomponent, beta polypeptide (C1qb) | -2.85 |
| Ddr1 | discoidin domain receptor family, member 1 (Ddr1), transcript variant 1 | -2.8 |
| Anxa3 | annexin A3 (Anxa3) | -2.82 |
| Sv2b | synaptic vesicle glycoprotein 2 b (Sv2b) | -2.77 |
| Serpine2 | serine (or cysteine) peptidase inhibitor, clade E, member 2 (Serpine2) | -2.86 |
| Anxa3 | annexin A3 (Anxa3) | -2.87 |
| Mobp | myelin-associated oligodendrocytic basic protein (Mobp), transcript variant 3 | -2.84 |
| Cmas | cytidine monophospho-N-acetylneuraminic acid synthetase (Cmas) | -2.86 |
| Rgs9 | regulator of G-protein signaling 9 (Rgs9) | -2.89 |
| Tpr | translocated promoter region (Tpr) | -2.93 |
| Gna13 | guanine nucleotide binding protein, alpha 13 (Gna13) | -2.95 |
| Zmynd11 | zinc finger, MYND domain containing 11 (Zmynd11) | -3.06 |
| Mrpl55 | mitochondrial ribosomal protein L55 (Mrpl55) | -3.01 |
| Napepld | N-acyl phosphatidylethanolamine phospholipase D (Napepld) | -3.14 |
| Nefm | neurofilament, medium polypeptide (Nefm) | -3.11 |
| Rnps1 | ribonucleic acid binding protein S1 (Rnps1), transcript variant 2 | -3.14 |
| Rab6 | RAB6, member RAS oncogene family (Rab6) | -3.19 |
| Mt3 | metallothionein 3 (Mt3) | -3.29 |
| 1190005F20Rik | RIKEN cDNA 1190005F20 gene (1190005F20Rik) | -3.36 |
| Nudt19 | nudix (nucleoside diphosphate linked moiety X)-type motif 19 (Nudt19) | -3.47 |
| Kcnf1 | potassium voltage-gated channel, subfamily F, member 1 (Kcnf1) | -3.56 |
| Tmem68 | transmembrane protein 68 (Tmem68) | -3.67 |
| C1ql2 | complement component 1, q subcomponent-like 2 (C1ql2) | -3.69 |
| LOC546015 | ribosomal protein S9 (LOC546015), misc RNA. | -3.73 |
| 5730469M10Rik | RIKEN cDNA 5730469M10 gene (5730469M10Rik) | -3.76 |
| Mobp | myelin-associated oligodendrocytic basic protein (Mobp), transcript variant 1 | -3.75 |
| Kcnh1 | potassium voltage-gated channel, subfamily H (eag-related), member 1 (Kcnh1), transcript variant 2 | -3.77 |
| Alg1 | asparagine-linked glycosylation 1 homolog (yeast, beta-1,4-mannosyltransferase) (Alg1) | -3.85 |
| Cbfa2t3h | core-binding factor, runt domain, alpha subunit 2, translocated to, 3 homolog (human) (Cbfa2t3h) | -4.28 |
| Acsl6 | acyl-CoA synthetase long-chain family member 6 (Acsl6), transcript variant 4 | -4.31 |
| Nudt19 | nudix (nucleoside diphosphate linked moiety X)-type motif 19 (Nudt19) | -4.78 |
| Med23 | mediator complex subunit 23 (Med23) | -4.76 |
| Rbm28 | RNA binding motif protein 28 (Rbm28), transcript variant 2 | -4.83 |
| Rgl1 | ral guanine nucleotide dissociation stimulator,-like 1 (Rgl1) | -4.81 |
| Hist1h2ao | histone cluster 1, H2ao (Hist1h2ao) | -4.94 |
| Ccndbp1 | cyclin D-type binding-protein 1 (Ccndbp1) | -4.95 |
| Hist1h2af | histone cluster 1, H2af (Hist1h2af) | -5.08 |
| Rapgefl1 | Rap guanine nucleotide exchange factor (GEF)-like 1 (Rapgefl1) | -4.95 |
| Cort | cortistatin (Cort) | -5.13 |
| Slc25a3 | solute carrier family 25 (mitochondrial carrier, phosphate carrier), member 3 (Slc25a3), nuclear gene encoding mitochondrial protein | -5.1 |
| 6430706D22Rik | RIKEN cDNA 6430706D22 gene (6430706D22Rik) | -5.18 |
| Dusp7 | dual specificity phosphatase 7 (Dusp7) | -5.23 |
| Sema5a | sema domain, seven thrombospondin repeats (type 1 and type 1-like), transmembrane domain (TM) and short cytoplasmic domain, (semaphorin) 5A (Sema5a) | -5.3 |
| Bsdc1 | BSD domain containing 1 (Bsdc1) | -5.42 |
| Cort | cortistatin (Cort) | -5.58 |
| BC056474 | cDNA sequence BC056474 (BC056474) | -5.68 |
| Evc2 | Ellis van Creveld syndrome 2 homolog (human) (Evc2) | -5.61 |
| Gde1 | glycerophosphodiester phosphodiesterase 1 (Gde1) | -5.59 |
| Hist1h2bm | histone cluster 1, H2bm (Hist1h2bm) | -5.68 |
| Ehd3 | EH-domain containing 3 (Ehd3) | -5.81 |
| Sdc4 | syndecan 4 (Sdc4) | -5.93 |
| Rbbp9 | retinoblastoma binding protein 9 (Rbbp9) | -5.97 |
| Psmb5 | proteasome (prosome, macropain) subunit, beta type 5 (Psmb5) | -5.98 |
| Slco1c1 | solute carrier organic anion transporter family, member 1c1 (Slco1c1) | -6.02 |
| Pak1 | p21 (CDKN1A)-activated kinase 1 (Pak1) | -6.01 |
| Ccrn4l | CCR4 carbon catabolite repression 4-like (S. cerevisiae) (Ccrn4l) | -6.32 |
| Scoc | short coiled-coil protein (Scoc), transcript variant 1 | -6.45 |
| Prdx2 | peroxiredoxin 2 (Prdx2) | -6.98 |
| Uap1 | UDP-N-acetylglucosamine pyrophosphorylase 1 (Uap1) | -7.04 |
| Sepw1 | selenoprotein W, muscle 1 (Sepw1) | -8.36 |
| 1200015F23Rik | RIKEN cDNA 1200015F23 gene (1200015F23Rik) | -8.73 |
| Sc4mol | sterol-C4-methyl oxidase-like (Sc4mol) | -9.17 |
| Atf4 | activating transcription factor 4 (Atf4) | -9.48 |
| Enpp5 | ectonucleotide pyrophosphatase/phosphodiesterase 5 (Enpp5) | -10.93 |
| Arl3 | ADP-ribosylation factor-like 3 (Arl3) | -11 |
| Hsp90b1 | heat shock protein 90, beta (Grp94), member 1 (Hsp90b1) | -12.28 |
| Fgfr1op2 | FGFR1 oncogene partner 2 (Fgfr1op2) | -12.74 |
| Csrp1 | cysteine and glycine-rich protein 1 (Csrp1) | -17.2 |
| Spink8 | serine peptidase inhibitor, Kazal type 8 (Spink8) | -17.61 |
|  |  |  |
