# Supplementary material for: Hippocampal Transcriptomic and Proteomic Alterations in the BTBR Mouse Model of Autism Spectrum Disorder
Source: Front Physiol. 2015 Nov 24;6:324. doi: 10.3389/fphys.2015.00324 (PMC4656818; doi:10.3389/fphys.2015.00324)
Supplement: Supplementary file 3 [file Table2.DOCX]

**Table S2. Significant differential mRNA transcript expression between BTBR and B6 cortex**. The table indicates the significant and differentially-expressed transcripts between BTBR and B6 mouse cortex. For each significantly-regulated (p<0.05) transcript the Official Gene Symbol, textual transcript Definition and expression Z ratio (BTBR versus B6) is given.

| **Gene Symbol** | **Definition** | **Z ratio** |
| --- | --- | --- |
| Serpina3n | serine (or cysteine) peptidase inhibitor, clade A, member 3N (Serpina3n) | 10.79 |
| Slc7a14 | solute carrier family 7 (cationic amino acid transporter, y+ system), member 14 (Slc7a14) | 10.35 |
| Lcmt1 | leucine carboxyl methyltransferase 1 (Lcmt1) | 5.16 |
| Ndufb10 | NADH dehydrogenase (ubiquinone) 1 beta subcomplex, 10 (Ndufb10) | 4.61 |
| Mrps10 | mitochondrial ribosomal protein S10 (Mrps10), nuclear gene encoding mitochondrial protein | 4.4 |
| Extl1 | exostoses (multiple)-like 1 (Extl1) | 4.42 |
| Cap1 | CAP, adenylate cyclase-associated protein 1 (yeast) (Cap1) | 4.36 |
| Lypd1 | Ly6/Plaur domain containing 1 (Lypd1) | 4.24 |
| Pcdh21 | protocadherin 21 (Pcdh21) | 3.97 |
| Grlf1 | glucocorticoid receptor DNA binding factor 1 (Grlf1) | 4.05 |
| Adi1 | acireductone dioxygenase 1 (Adi1) | 3.9 |
| Iqgap1 | IQ motif containing GTPase activating protein 1 (Iqgap1) | 3.77 |
| Rsph1 | radial spoke head 1 homolog (Chlamydomonas) (Rsph1) | 3.74 |
| Usp29 | ubiquitin specific peptidase 29 (Usp29) | 3.7 |
| Vars2 | valyl-tRNA synthetase 2, mitochondrial (putative) (Vars2) | 3.64 |
| Centg1 | centaurin, gamma 1 (Centg1) | 3.71 |
| 2310002B06Rik | RIKEN cDNA 2310002B06 gene (2310002B06Rik) | 3.57 |
| LOC100046744 | Serine/arginine repetitive matrix protein 2 (LOC100046744) | 3.66 |
| Snx30 | sorting nexin family member 30 (Snx30) | 3.45 |
| LOC677317 | Mod1 protein, transcript variant 4 (LOC677317) | 3.23 |
| Iap | alkaline phosphatase, intestinal (Iap) | 3.43 |
| Olfml1 | olfactomedin-like 1 (Olfml1) | 3.16 |
| 4933439C20Rik | RIKEN cDNA 4933439C20 gene (4933439C20Rik) | 3.36 |
| Kras | v-Ki-ras2 Kirsten rat sarcoma viral oncogene homolog (Kras) | 3.08 |
| Nnat | neuronatin (Nnat), transcript variant 1 | 2.89 |
| Gnai2 | guanine nucleotide binding protein, alpha inhibiting 2 (Gnai2) | 2.89 |
| Nubp2 | nucleotide binding protein 2 (Nubp2) | 2.79 |
| Cox18 | COX18 cytochrome c oxidase assembly homolog (S. cerevisiae) (Cox18) | 2.75 |
| Hmgn2 | high mobility group nucleosomal binding domain 2 (Hmgn2) | 2.75 |
| Blvrb | biliverdin reductase B (flavin reductase (NADPH)) (Blvrb) | 2.54 |
| Asah3l | N-acylsphingosine amidohydrolase 3-like (Asah3l) | 2.47 |
| Nicn1 | nicolin 1 (Nicn1) | 2.71 |
| Anxa5 | annexin A5 (Anxa5) | 2.56 |
| 1200016B10Rik | RIKEN cDNA 1200016B10 gene (1200016B10Rik) | 2.5 |
| Slc6a13 | solute carrier family 6 (neurotransmitter transporter, GABA), member 13 (Slc6a13) | 2.46 |
| Scg5 | secretogranin V (Scg5) | 2.68 |
| Osbpl3 | oxysterol binding protein-like 3 (Osbpl3) | 2.34 |
| Ptgds | prostaglandin D2 synthase (brain) (Ptgds) | 2.62 |
| Sorl1 | sortilin-related receptor, LDLR class A repeats-containing (Sorl1) | 2.52 |
| 4930539E08Rik | RIKEN cDNA 4930539E08 gene (4930539E08Rik) | 2.27 |
| Clec16a | C-type lectin domain family 16, member A (Clec16a) | 2.26 |
| Ndn | necdin (Ndn) | 2.51 |
| Zfp94 | zinc finger protein 94 (Zfp94) | 2.19 |
| B6ip | B6-interacting protein (B6ip) | 2.2 |
| Mfsd11 | major facilitator superfamily domain containing 11 (Mfsd11) | 2.15 |
| Lpin2 | lipin 2 (Lpin2) | 2.13 |
| Anapc13 | anaphase promoting complex subunit 13 (Anapc13) | 2.31 |
| Ahdc1 | AT hook, DNA binding motif, containing 1 (Ahdc1) | 2.18 |
| Mrps10 | mitochondrial ribosomal protein S10 (Mrps10), nuclear gene encoding mitochondrial protein | 2.15 |
| B930076A02 | transmembrane protein 145 (Tmem145) | 2.2 |
| Rab24 | RAB24, member RAS oncogene family (Rab24) | 2.14 |
| Dbnl | drebrin-like (Dbnl) | 2.08 |
| Ntn2l | netrin 2-like (chicken) (Ntn2l) | 2.06 |
| Gins4 | GINS complex subunit 4 (Sld5 homolog) (Gins4) | 2.14 |
| Ccrk | cell cycle related kinase (Ccrk) | 2.03 |
| Chd8 | chromodomain helicase DNA binding protein 8 (Chd8) | 2.11 |
| Ranbp3l | RAN binding protein 3-like (Ranbp3l) | 2.07 |
| Sox21 | SRY-box containing gene 21 (Sox21) | 2.04 |
| Bbs7 | Bardet-Biedl syndrome 7 (Bbs7) | 2.03 |
| Gm129 | gene model 129, (NCBI) (Gm129) | 1.98 |
| Wfs1 | Wolfram syndrome 1 homolog (human) (Wfs1) | 2.03 |
| Gria1 | glutamate receptor, ionotropic, AMPA1 (alpha 1) (Gria1) | 2.09 |
| Ap2s1 | adaptor-related protein complex 2, sigma 1 subunit (Ap2s1) | 1.99 |
| B9d1 | B9 protein domain 1 (B9d1) | 2 |
| Fbxo6 | F-box protein 6 (Fbxo6) | 2.07 |
| Vps33b | vacuolar protein sorting 33B (yeast) (Vps33b) | 1.86 |
| Junb | Jun-B oncogene (Junb) | 1.96 |
| Nudc | nuclear distribution gene C homolog (Aspergillus) (Nudc) | 2.16 |
| Grp | gastrin releasing peptide (Grp) | 1.79 |
| 1190002H23Rik | RIKEN cDNA 1190002H23 gene (1190002H23Rik) | 1.95 |
| Prkd3 | protein kinase D3 (Prkd3) | 1.9 |
| Gfer | growth factor, erv1 (S. cerevisiae)-like (augmenter of liver regeneration) (Gfer) | 1.93 |
| Gria1 | glutamate receptor, ionotropic, AMPA1 (alpha 1) (Gria1) | 1.95 |
| Centg1 | centaurin, gamma 1 (Centg1) | 1.79 |
| Spag5 | sperm associated antigen 5 (Spag5) | 1.8 |
| 1810073G14Rik | RIKEN cDNA 1810073G14 gene (1810073G14Rik) | 1.85 |
| Osbpl3 | oxysterol binding protein-like 3 (Osbpl3) | 1.77 |
| Atp9b | ATPase, class II, type 9B (Atp9b) | 1.8 |
| Foxred1 | FAD-dependent oxidoreductase domain containing 1 (Foxred1) | 1.79 |
| Map3k3 | mitogen-activated protein kinase kinase kinase 3 (Map3k3) | 1.76 |
| Gria1 | glutamate receptor, ionotropic, AMPA1 (alpha 1) (Gria1) | 1.9 |
| Uba5 | ubiquitin-like modifier activating enzyme 5 (Uba5) | 1.87 |
| Tmem2 | transmembrane protein 2 (Tmem2), transcript variant 2 | 1.72 |
| Irf2bp1 | interferon regulatory factor 2 binding protein 1 (Irf2bp1) | 1.79 |
| Guk1 | guanylate kinase 1 (Guk1) | 1.96 |
| Gm129 | gene model 129, (NCBI) (Gm129) | 1.71 |
| Foxn3 | forkhead box N3 (Foxn3) | 1.71 |
| Stxbp2 | syntaxin binding protein 2 (Stxbp2) | 1.69 |
| Mrpl33 | mitochondrial ribosomal protein L33 (Mrpl33), nuclear gene encoding mitochondrial protein | 1.83 |
| Tmsb10 | thymosin, beta 10 (Tmsb10) | 1.81 |
| Mocs1 | molybdenum cofactor synthesis 1 (Mocs1), transcript variant 1 | 1.65 |
| Abhd14b | abhydrolase domain containing 14b (Abhd14b) | 1.71 |
| Tcea2 | transcription elongation factor A (SII), 2 (Tcea2) | 1.74 |
| Nudc | nuclear distribution gene C homolog (Aspergillus) (Nudc) | 1.62 |
| Gp38 | podoplanin (Pdpn) | 1.66 |
| Vdac2 | voltage-dependent anion channel 2 (Vdac2) | 1.7 |
| Prkd2 | protein kinase D2 (Prkd2) | 1.53 |
| Gorasp1 | golgi reassembly stacking protein 1 (Gorasp1) | 1.62 |
| Slc25a3 | solute carrier family 25 (mitochondrial carrier, phosphate carrier), member 3 (Slc25a3) | 1.61 |
| Pigt | phosphatidylinositol glycan anchor biosynthesis, class T (Pigt) | 1.62 |
| EG328644 | predicted gene, EG328644 (EG328644) | 1.55 |
| BC021381 | cDNA sequence BC021381 (BC021381) | 1.53 |
| BC057371 | cDNA sequence BC057371 (BC057371) | 1.51 |
| Srrm2 | serine/arginine repetitive matrix 2 (Srrm2) | 1.62 |
| Slc7a10 | solute carrier family 7 (cationic amino acid transporter, y+ system), member 10 (Slc7a10) | 1.53 |
| Pcbd2 | pterin 4 alpha carbinolamine dehydratase/dimerization cofactor of hepatocyte nuclear factor 1 alpha (TCF1) 2 (Pcbd2) | 1.6 |
| Tnrc6a | trinucleotide repeat containing 6a (Tnrc6a) | 1.64 |
| Itgb1bp1 | integrin beta 1 binding protein 1 (Itgb1bp1) | 1.54 |
| LOC100044298 | PREDICTED: hypothetical protein LOC100044298 (LOC100044298) | 1.61 |
| Tnrc6a | trinucleotide repeat containing 6a (Tnrc6a) | 1.53 |
| Rab24 | RAB24, member RAS oncogene family (Rab24) | 1.57 |
| Src | Rous sarcoma oncogene (Src), transcript variant 2 | 1.53 |
| Rab24 | RAB24, member RAS oncogene family (Rab24) | 1.53 |
| Ebpl | emopamil binding protein-like (Ebpl) | 1.53 |
| Akap8l | A kinase (PRKA) anchor protein 8-like (Akap8l) | 1.57 |
| Adcy2 | adenylate cyclase 2 (Adcy2) | 1.62 |
| Psmb10 | proteasome (prosome, macropain) subunit, beta type 10 (Psmb10) | 1.5 |
| Eno1 | enolase 1, alpha non-neuron (Eno1) | 1.61 |
| Dbp | D site albumin promoter binding protein (Dbp) | 1.56 |
| LOC546015 | ribosomal protein S9 (LOC546015), misc RNA. | 1.68 |
| 1500032D16Rik | RIKEN cDNA 1500032D16 gene (1500032D16Rik) | 1.53 |
| Lrrc49 | leucine rich repeat containing 49 (Lrrc49) | 1.53 |
| Prr13 | proline rich 13 (Prr13) | 1.52 |
| Tmsb10 | thymosin, beta 10 (Tmsb10) | 1.57 |
| Sae1 | SUMO1 activating enzyme subunit 1 (Sae1) | 1.53 |
| Rpn2 | ribophorin II (Rpn2) | 1.53 |
| Rps2 | ribosomal protein S2 (Rps2) | 1.51 |
| Elavl4 | ELAV (embryonic lethal, abnormal vision, Drosophila)-like 4 (Hu antigen D) (Elavl4), transcript variant 1 | -1.56 |
| Iqgap2 | IQ motif containing GTPase activating protein 2 (Iqgap2) | -1.59 |
| Gtf3c1 | general transcription factor III C 1 (Gtf3c1) | -1.56 |
| Ica1 | islet cell autoantigen 1 (Ica1) | -1.51 |
| Cib2 | calcium and integrin binding family member 2 (Cib2) | -1.54 |
| Vip | vasoactive intestinal polypeptide (Vip) | -1.55 |
| Rnf11 | ring finger protein 11 (Rnf11) | -1.59 |
| Ccdc132 | coiled-coil domain containing 132 (Ccdc132) | -1.54 |
| Lyrm5 | LYR motif containing 5 (Lyrm5) | -1.57 |
| Enpp4 | ectonucleotide pyrophosphatase/phosphodiesterase 4 (Enpp4) | -1.59 |
| Mettl3 | methyltransferase-like 3 (Mettl3) | -1.58 |
| AU022870 | expressed sequence AU022870 (AU022870) | -1.57 |
| Gna13 | guanine nucleotide binding protein, alpha 13 (Gna13) | -1.52 |
| Med7 | mediator complex subunit 7 (Med7) | -1.58 |
| Chmp4b | chromatin modifying protein 4B (Chmp4b) | -1.52 |
| Mertk | c-mer proto-oncogene tyrosine kinase (Mertk) | -1.63 |
| Ppapdc2 | phosphatidic acid phosphatase type 2 domain containing 2 (Ppapdc2) | -1.59 |
| Rnpep | arginyl aminopeptidase (aminopeptidase B) (Rnpep) | -1.65 |
| Ykt6 | YKT6 homolog (S. Cerevisiae) (Ykt6) | -1.56 |
| Fbxo34 | F-box protein 34 (Fbxo34) | -1.53 |
| Hdhd2 | haloacid dehalogenase-like hydrolase domain containing 2 (Hdhd2), transcript variant 1 | -1.68 |
| Atg3 | autophagy-related 3 (yeast) (Atg3) | -1.74 |
| BC055107 | cDNA sequence BC055107 (BC055107) | -1.51 |
| Homer1 | homer homolog 1 (Drosophila) (Homer1), transcript variant d | -1.54 |
| Capns1 | calpain, small subunit 1 (Capns1) | -1.63 |
| Rpo1-3 | RNA polymerase 1-3 (Rpo1-3), transcript variant 1 | -1.58 |
| Bok | BCL2-related ovarian killer (Bok) | -1.59 |
| Uchl3 | ubiquitin carboxyl-terminal esterase L3 (ubiquitin thiolesterase) (Uchl3) | -1.74 |
| Pcdha6 | protocadherin alpha 6 (Pcdha6) | -1.67 |
| Flt1 | FMS-like tyrosine kinase 1 (Flt1) | -1.6 |
| Ppm1l | protein phosphatase 1 (formerly 2C)-like (Ppm1l) | -1.72 |
| Itpka | inositol 1,4,5-trisphosphate 3-kinase A (Itpka) | -1.54 |
| BC025076 | membrane magnesium transporter 2 (Mmgt2) | -1.73 |
| Usp33 | ubiquitin specific peptidase 33 (Usp33), transcript variant 1 | -1.6 |
| Adar | adenosine deaminase, RNA-specific (Adar), transcript variant 1 | -1.75 |
| Rnf166 | ring finger protein 166 (Rnf166) | -1.72 |
| Tmem56 | transmembrane protein 56 (Tmem56) | -1.66 |
| Atp5g2 | ATP synthase, H+ transporting, mitochondrial F0 complex, subunit c (subunit 9), isoform 2 (Atp5g2) | -1.72 |
| Dusp14 | dual specificity phosphatase 14 (Dusp14) | -1.74 |
| Homer1 | homer homolog 1 (Drosophila) (Homer1), transcript variant d | -1.59 |
| Dmtf1 | cyclin D binding myb-like transcription factor 1 (Dmtf1) | -1.7 |
| Jdp2 | Jun dimerization protein 2 (Jdp2) | -1.78 |
| Caskin1 | CASK interacting protein 1 (Caskin1) | -1.73 |
| 4930570C03Rik | RIKEN cDNA 4930570C03 gene (4930570C03Rik) | -1.75 |
| Nrxn1 | neurexin I (Nrxn1) | -1.72 |
| C1qc | complement component 1, q subcomponent, C chain (C1qc) | -1.76 |
| Il33 | interleukin 33 (Il33) | -1.75 |
| Tex261 | testis expressed gene 261 (Tex261) | -1.74 |
| Ttc17 | tetratricopeptide repeat domain 17 (Ttc17) | -1.75 |
| Crym | crystallin, mu (Crym) | -1.66 |
| Efhd1 | EF hand domain containing 1 (Efhd1) | -1.78 |
| Snrpd1 | small nuclear ribonucleoprotein D1 (Snrpd1) | -1.67 |
| Enpp6 | ectonucleotide pyrophosphatase/phosphodiesterase 6 (Enpp6) | -1.9 |
| 1500019G21Rik | RIKEN cDNA 1500019G21 gene (1500019G21Rik) | -1.85 |
| Stac2 | SH3 and cysteine rich domain 2 (Stac2) | -1.8 |
| Rcan2 | regulator of calcineurin 2 (Rcan2), transcript variant 1 | -1.7 |
| Darc | Duffy blood group, chemokine receptor (Darc) | -1.72 |
| Luzp2 | leucine zipper protein 2 (Luzp2) | -1.75 |
| Xbp1 | X-box binding protein 1 (Xbp1) | -1.66 |
| Nrn1 | neuritin 1 (Nrn1) | -1.62 |
| Igsf3 | immunoglobulin superfamily, member 3 (Igsf3) | -1.89 |
| Ap1gbp1 | AP1 gamma subunit binding protein 1 (Ap1gbp1), transcript variant 2 | -1.81 |
| Rrm2b | ribonucleotide reductase M2 B (TP53 inducible) (Rrm2b) | -1.88 |
| Clcn7 | chloride channel 7 (Clcn7) | -1.9 |
| Yaf2 | YY1 associated factor 2 (Yaf2) | -1.75 |
| Cpne9 | copine family member IX (Cpne9) | -1.76 |
| Nipsnap1 | 4-nitrophenylphosphatase domain and non-neuronal SNAP25-like protein homolog 1 (C. elegans) (Nipsnap1) | -1.78 |
| Mat2b | methionine adenosyltransferase II, beta (Mat2b) | -1.86 |
| Snrpd1 | small nuclear ribonucleoprotein D1 (Snrpd1) | -1.81 |
| Anxa3 | annexin A3 (Anxa3) | -1.93 |
| Mrpl3 | mitochondrial ribosomal protein L3 (Mrpl3), nuclear gene encoding mitochondrial protein | -1.96 |
| Anxa3 | annexin A3 (Anxa3) | -1.91 |
| Trpc7 | transient receptor potential cation channel, subfamily C, member 7 (Trpc7) | -2.01 |
| 1700123O20Rik | RIKEN cDNA 1700123O20 gene (1700123O20Rik) | -1.92 |
| Sema3a | sema domain, immunoglobulin domain (Ig), short basic domain, secreted, (semaphorin) 3A (Sema3a) | -1.99 |
| Cib2 | calcium and integrin binding family member 2 (Cib2) | -2.04 |
| Ptp4a2 | protein tyrosine phosphatase 4a2 (Ptp4a2) | -2.1 |
| Dpp10 | dipeptidylpeptidase 10 (Dpp10) | -1.91 |
| Lancl1 | LanC (bacterial lantibiotic synthetase component C)-like 1 (Lancl1) | -1.91 |
| Fbxo34 | F-box protein 34 (Fbxo34) | -1.93 |
| Hsd11b1 | hydroxysteroid 11-beta dehydrogenase 1 (Hsd11b1), transcript variant 1 | -2.01 |
| Fmn2 | formin 2 (Fmn2) | -2.06 |
| Trf | transferrin (Trf) | -1.94 |
| Ints7 | integrator complex subunit 7 (Ints7) | -2.11 |
| Rab6 | RAB6, member RAS oncogene family (Rab6) | -2.05 |
| Hsd11b1 | hydroxysteroid 11-beta dehydrogenase 1 (Hsd11b1), transcript variant 1 | -2.05 |
| Gabrg2 | gamma-aminobutyric acid (GABA-A) receptor, subunit gamma 2 (Gabrg2), transcript variant 2 | -2.2 |
| Anln | anillin, actin binding protein (scraps homolog, Drosophila) (Anln) | -2.16 |
| Rpo1-3 | RNA polymerase 1-3 (Rpo1-3), transcript variant 1 | -2.07 |
| B2m | beta-2 microglobulin (B2m) | -2 |
| Rgs7 | regulator of G protein signaling 7 (Rgs7) | -1.99 |
| Pon2 | paraoxonase 2 (Pon2) | -1.98 |
| Reep3 | receptor accessory protein 3 (Reep3) | -2.13 |
| Hist2h2ac | histone cluster 2, H2ac (Hist2h2ac) | -2.03 |
| 1600014C10Rik | RIKEN cDNA 1600014C10 gene (1600014C10Rik) | -2.24 |
| Ppm2c | protein phosphatase 2C, magnesium dependent, catalytic subunit (Ppm2c), nuclear gene encoding mitochondrial protein, transcript variant 1 | -2.25 |
| Lancl1 | LanC (bacterial lantibiotic synthetase component C)-like 1 (Lancl1) | -2.13 |
| 6330403K07Rik | RIKEN cDNA 6330403K07 gene (6330403K07Rik) | -1.98 |
| Ube2m | ubiquitin-conjugating enzyme E2M (UBC12 homolog, yeast) (Ube2m) | -2.3 |
| Ppm1m | protein phosphatase 1M (Ppm1m), transcript variant 1 | -2.32 |
| LOC100048331 | DnaJ (Hsp40) homolog, subfamily A, member 4 (LOC100048331) | -2.33 |
| Nefm | neurofilament, medium polypeptide (Nefm) | -2.42 |
| Mrpl55 | mitochondrial ribosomal protein L55 (Mrpl55) | -2.38 |
| Drd1a | dopamine receptor D1A (Drd1a) | -2.28 |
| AI316807 | expressed sequence AI316807 (AI316807) | -2.22 |
| Rpo2tc1 | SUB1 homolog (S. cerevisiae) (Rpo2tc1) | -2.2 |
| Tsc2 | tuberous sclerosis 2 (Tsc2), transcript variant 2 | -2.21 |
| Mrpl48 | mitochondrial ribosomal protein L48 (Mrpl48), nuclear gene encoding mitochondrial protein | -2.3 |
| Entpd4 | ectonucleoside triphosphate diphosphohydrolase 4 (Entpd4) | -2.19 |
| Serpine2 | serine (or cysteine) peptidase inhibitor, clade E, member 2 (Serpine2) | -2.43 |
| Mobp | myelin-associated oligodendrocytic basic protein (Mobp), transcript variant 1 | -2.29 |
| Tex261 | testis expressed gene 261 (Tex261) | -2.45 |
| Tnnc1 | troponin C, cardiac/slow skeletal (Tnnc1) | -2.47 |
| Gnptab | N-acetylglucosamine-1-phosphate transferase, alpha and beta subunits (Gnptab) | -2.5 |
| Mrps23 | mitochondrial ribosomal protein S23 (Mrps23), nuclear gene encoding mitochondrial protein | -2.48 |
| Mrpl48 | mitochondrial ribosomal protein L48 (Mrpl48), transcript variant 1 | -2.45 |
| Ddit4l | DNA-damage-inducible transcript 4-like (Ddit4l) | -2.48 |
| Zfp131 | zinc finger protein 131 (Zfp131) | -2.44 |
| Rpl29 | ribosomal protein L29 (Rpl29) | -2.36 |
| B2m | beta-2 microglobulin (B2m) | -2.41 |
| Hspb6 | heat shock protein, alpha-crystallin-related, B6 (Hspb6) | -2.59 |
| Dmwd | dystrophia myotonica-containing WD repeat motif (Dmwd) | -2.48 |
| Pfdn5 | prefoldin 5 (Pfdn5) | -2.34 |
| Ccdc59 | coiled-coil domain containing 59 (Ccdc59) | -2.61 |
| Mt3 | metallothionein 3 (Mt3) | -2.45 |
| Med23 | mediator complex subunit 23 (Med23) | -2.59 |
| Drctnnb1a | family with sequence similarity 126, member A (Drctnnb1a) | -2.64 |
| 1190005F20Rik | RIKEN cDNA 1190005F20 gene (1190005F20Rik) | -2.65 |
| Zmynd11 | zinc finger, MYND domain containing 11 (Zmynd11) | -2.66 |
| Pfdn5 | prefoldin 5 (Pfdn5) | -2.48 |
| Gtpbp4 | GTP binding protein 4 (Gtpbp4) | -2.7 |
| Riok1 | RIO kinase 1 (yeast) (Riok1) | -2.8 |
| Ankrd56 | ankyrin repeat domain 56 (Ankrd56) | -2.77 |
| Stt3b | STT3, subunit of the oligosaccharyltransferase complex, homolog B (S. cerevisiae) (Stt3b) | -2.61 |
| Pop4 | processing of precursor 4, ribonuclease P/MRP family, (S. cerevisiae) (Pop4) | -2.79 |
| Atf7ip | activating transcription factor 7 interacting protein (Atf7ip) | -2.91 |
| Ddr1 | discoidin domain receptor family, member 1 (Ddr1), transcript variant 1 | -2.92 |
| 1700025G04Rik | RIKEN cDNA 1700025G04 gene (1700025G04Rik) | -2.92 |
| C1qb | complement component 1, q subcomponent, beta polypeptide (C1qb) | -2.94 |
| Sv2b | synaptic vesicle glycoprotein 2 b (Sv2b) | -2.76 |
| Bdnf | brain derived neurotrophic factor (Bdnf), transcript variant 3 | -3.02 |
| LOC100040573 | putative transcription factor ZNF131, transcript variant 1 (LOC100040573) | -2.95 |
| Nudt19 | nudix (nucleoside diphosphate linked moiety X)-type motif 19 (Nudt19) | -3.23 |
| Tpr | translocated promoter region (Tpr) | -3.21 |
| Kcnf1 | potassium voltage-gated channel, subfamily F, member 1 (Kcnf1) | -3.23 |
| LOC546015 | ribosomal protein S9 (LOC546015), misc RNA. | -3.3 |
| Alg1 | asparagine-linked glycosylation 1 homolog (yeast, beta-1,4-mannosyltransferase) (Alg1) | -3.47 |
| Rpp25 | ribonuclease P 25 subunit (human) (Rpp25) | -3.56 |
| 6430706D22Rik | RIKEN cDNA 6430706D22 gene (6430706D22Rik) | -3.58 |
| Myl4 | myosin, light polypeptide 4 (Myl4) | -3.56 |
| Rbm28 | RNA binding motif protein 28 (Rbm28), transcript variant 2 | -3.68 |
| Rnps1 | ribonucleic acid binding protein S1 (Rnps1), transcript variant 2 | -3.64 |
| Cox6a2 | cytochrome c oxidase, subunit VI a, polypeptide 2 (Cox6a2), nuclear gene encoding mitochondrial protein | -3.87 |
| Kcnh1 | potassium voltage-gated channel, subfamily H (eag-related), member 1 (Kcnh1), transcript variant 2 | -3.86 |
| Cmas | cytidine monophospho-N-acetylneuraminic acid synthetase (Cmas) | -3.84 |
| BC056474 | cDNA sequence BC056474 (BC056474) | -4.04 |
| LOC100045304 | neurofilament protein (LOC100045304) | -4.06 |
| Hist1h2an | histone cluster 1, H2an (Hist1h2an) | -4.07 |
| Rab6 | RAB6, member RAS oncogene family (Rab6) | -3.96 |
| Cort | cortistatin (Cort) | -4.24 |
| Cbfa2t3h | core-binding factor, runt domain, alpha subunit 2, translocated to, 3 homolog (human) (Cbfa2t3h) | -4.24 |
| 1200003I07Rik | RIKEN cDNA 1200003I07 gene (1200003I07Rik), transcript variant 3 | -4.3 |
| 5730469M10Rik | RIKEN cDNA 5730469M10 gene (5730469M10Rik) | -4.22 |
| D14Ertd449e | DNA segment, Chr 14, ERATO Doi 449, expressed (D14Ertd449e) | -4.45 |
| Ehd3 | EH-domain containing 3 (Ehd3) | -4.48 |
| Med23 | mediator complex subunit 23 (Med23) | -4.49 |
| Rapgefl1 | Rap guanine nucleotide exchange factor (GEF)-like 1 (Rapgefl1) | -4.41 |
| Cort | cortistatin (Cort) | -4.63 |
| Tmem68 | transmembrane protein 68 (Tmem68) | -4.75 |
| Hist1h2ao | histone cluster 1, H2ao (Hist1h2ao) | -4.77 |
| Acsl6 | acyl-CoA synthetase long-chain family member 6 (Acsl6), transcript variant 4 | -4.95 |
| Hist1h2af | histone cluster 1, H2af (Hist1h2af) | -5.02 |
| Nudt19 | nudix (nucleoside diphosphate linked moiety X)-type motif 19 (Nudt19) | -4.91 |
| Bsdc1 | BSD domain containing 1 (Bsdc1) | -5.05 |
| Sema5a | sema domain, seven thrombospondin repeats (type 1 and type 1-like), transmembrane domain (TM) and short cytoplasmic domain, (semaphorin) 5A | -5.09 |
| Psmb5 | proteasome (prosome, macropain) subunit, beta type 5 (Psmb5) | -5.09 |
| Sdc4 | syndecan 4 (Sdc4) | -5.37 |
| Dusp7 | dual specificity phosphatase 7 (Dusp7) | -5.24 |
| Kcnv1 | potassium channel, subfamily V, member 1 (Kcnv1) | -5.47 |
| Csnrp3 | cysteine-serine-rich nuclear protein 3 (Csnrp3) | -5.49 |
| Sema5a | sema domain, seven thrombospondin repeats (type 1 and type 1-like), transmembrane domain (TM) and short cytoplasmic domain, (semaphorin) 5A (Sema5a) | -5.46 |
| Ccndbp1 | cyclin D-type binding-protein 1 (Ccndbp1) | -5.79 |
| Rgl1 | ral guanine nucleotide dissociation stimulator,-like 1 (Rgl1) | -5.75 |
| Slco1c1 | solute carrier organic anion transporter family, member 1c1 (Slco1c1) | -6.42 |
| Gde1 | glycerophosphodiester phosphodiesterase 1 (Gde1) | -6.63 |
| Sepw1 | selenoprotein W, muscle 1 (Sepw1) | -6.65 |
| Rbbp9 | retinoblastoma binding protein 9 (Rbbp9) | -7.14 |
| Slc25a3 | solute carrier family 25 (mitochondrial carrier, phosphate carrier), member 3 (Slc25a3), nuclear gene encoding mitochondrial protein | -7.13 |
| Prdx2 | peroxiredoxin 2 (Prdx2) | -7.24 |
| Scoc | short coiled-coil protein (Scoc), transcript variant 1 | -7.59 |
| Ccrn4l | CCR4 carbon catabolite repression 4-like (S. cerevisiae) (Ccrn4l) | -7.67 |
| Pak1 | p21 (CDKN1A)-activated kinase 1 (Pak1) | -7.87 |
| Krt12 | keratin 12 (Krt12) | -8.44 |
| Uap1 | UDP-N-acetylglucosamine pyrophosphorylase 1 (Uap1) | -8.89 |
| Sc4mol | sterol-C4-methyl oxidase-like (Sc4mol) | -9.49 |
| Atf4 | activating transcription factor 4 (Atf4) | -10.14 |
| Arl3 | ADP-ribosylation factor-like 3 (Arl3) | -10.32 |
| Enpp5 | ectonucleotide pyrophosphatase/phosphodiesterase 5 (Enpp5) | -11.79 |
| Fgfr1op2 | FGFR1 oncogene partner 2 (Fgfr1op2) | -12.82 |
| Hsp90b1 | heat shock protein 90, beta (Grp94), member 1 (Hsp90b1) | -12.86 |
| Csrp1 | cysteine and glycine-rich protein 1 (Csrp1) | -17.6 |
