# Supplementary material for: Hippocampal Transcriptomic and Proteomic Alterations in the BTBR Mouse Model of Autism Spectrum Disorder
Source: Front Physiol. 2015 Nov 24;6:324. doi: 10.3389/fphys.2015.00324 (PMC4656818; doi:10.3389/fphys.2015.00324)
Supplement: Supplementary file 4 [file Table3.DOCX]

**Table S3. VennPlex Venn diagram analytical output for differentially-regulated transcripts between BTBR versus B6 Hippocampus and Cortex.** For each significantly-regulated (p<0.05) transcript the Official Gene Symbol and tissue-specific expression Z ratio (BTBR versus B6) is given. VennPlex was employed to generate the specific numerical Venn subsets depicted in Fig. 1B.

| **Gene Symbol** | **Hippocampus Z ratio** | **Cortex Z ratio** |
| --- | --- | --- |
| Slc7a14 | 12.11 | 10.35 |
| Serpina3n | 7.87 | 10.79 |
| Lypd1 | 6.17 | 4.24 |
| Lcmt1 | 5.25 | 5.16 |
| Cap1 | 4.97 | 4.36 |
| Mrps10 | 4.42 | 4.4 |
| Grlf1 | 4.24 | 4.05 |
| LOC677317 | 4.16 | 3.23 |
| Extl1 | 4.1 | 4.42 |
| Kras | 3.87 | 3.08 |
| 2310002B06Rik | 3.76 | 3.57 |
| Usp29 | 3.7 | 3.7 |
| Centg1 | 3.54 | 3.71 |
| Adi1 | 3.4 | 3.9 |
| Ndufb10 | 3.39 | 4.61 |
| Iqgap1 | 3.38 | 3.77 |
| Eno1 | 3.25 | 1.61 |
| Nubp2 | 3.17 | 2.79 |
| LOC100046744 | 3.16 | 3.66 |
| Snx30 | 3.16 | 3.45 |
| Iap | 3.08 | 3.43 |
| Slc25a3 | 2.99 | 1.61 |
| Scg5 | 2.94 | 2.68 |
| Sorl1 | 2.89 | 2.52 |
| Hmgn2 | 2.79 | 2.75 |
| 4933439C20Rik | 2.75 | 3.36 |
| Cox18 | 2.6 | 2.75 |
| Zfp94 | 2.53 | 2.19 |
| Blvrb | 2.53 | 2.54 |
| Nicn1 | 2.44 | 2.71 |
| Sox21 | 2.41 | 2.04 |
| Bbs7 | 2.38 | 2.03 |
| Ntn2l | 2.31 | 2.06 |
| Uba5 | 2.31 | 1.87 |
| Ndn | 2.33 | 2.51 |
| Lrrc49 | 2.3 | 1.53 |
| Ap2s1 | 2.24 | 1.99 |
| Dbnl | 2.21 | 2.08 |
| Gins4 | 2.2 | 2.14 |
| Olfml1 | 2.2 | 3.16 |
| Asah3l | 2.07 | 2.47 |
| Vdac2 | 2.02 | 1.7 |
| 1190002H23Rik | 2 | 1.95 |
| Chd8 | 1.98 | 2.11 |
| Clec16a | 1.93 | 2.26 |
| Foxred1 | 1.94 | 1.79 |
| Nudc | 1.87 | 2.16 |
| Gria1 | 1.88 | 2.09 |
| Irf2bp1 | 1.8 | 1.79 |
| Lpin2 | 1.81 | 2.13 |
| BC057371 | 1.75 | 1.51 |
| Guk1 | 1.76 | 1.96 |
| Anxa5 | 1.7 | 2.56 |
| Gfer | 1.72 | 1.93 |
| Ahdc1 | 1.69 | 2.18 |
| Fbxo6 | 1.71 | 2.07 |
| Sae1 | 1.71 | 1.53 |
| Prr13 | 1.59 | 1.52 |
| Wfs1 | 1.66 | 2.03 |
| B9d1 | 1.58 | 2 |
| Gorasp1 | 1.54 | 1.62 |
| Prkd2 | 1.55 | 1.53 |
| Vps33b | 1.51 | 1.86 |
| Rps2 | 1.55 | 1.51 |
| Mrpl33 | 1.51 | 1.83 |
|  |  |  |
| Rab6 | -1.52 | -2.05 |
| Tex261 | -1.58 | -1.74 |
| Snrpd1 | -1.51 | -1.67 |
| Nrxn1 | -1.53 | -1.72 |
| Chmp4b | -1.55 | -1.52 |
| Rpo2tc1 | -1.52 | -2.2 |
| Stac2 | -1.56 | -1.8 |
| Fmn2 | -1.58 | -2.06 |
| Arl3 | -1.63 | -10.32 |
| Hsd11b1 | -1.61 | -1.65 |
| Ddit4l | -1.55 | -2.48 |
| BC025076 | -1.71 | -1.73 |
| Bdnf | -1.73 | -3.02 |
| Rnf11 | -1.72 | -1.59 |
| Ppapdc2 | -1.76 | -1.59 |
| Ppm1m | -1.8 | -2.32 |
| Ttc17 | -1.84 | -1.75 |
| B2m | -1.85 | -2 |
| Ints7 | -1.91 | -2.11 |
| Gnptab | -1.98 | -2.5 |
| Hist2h2ac | -2.04 | -2.03 |
| Gtpbp4 | -2.05 | -2.7 |
| Clcn7 | -2.04 | -1.9 |
| LOC100040573 | -2.12 | -2.95 |
| Rnf166 | -2.17 | -1.72 |
| Mrpl48 | -2.16 | -2.3 |
| Il33 | -2.2 | -1.75 |
| Tsc2 | -2.14 | -2.21 |
| Rnpep | -2.2 | -1.65 |
| Entpd4 | -2.17 | -2.19 |
| Cib2 | -2.19 | -1.54 |
| Capns1 | -2.31 | -1.63 |
| 1700123O20Rik | -2.19 | -1.92 |
| Mrps23 | -2.21 | -2.48 |
| Zfp131 | -2.21 | -2.44 |
| Rpl29 | -2.24 | -2.36 |
| Stt3b | -2.21 | -2.61 |
| Dmwd | -2.28 | -2.48 |
| Caskin1 | -2.37 | -1.73 |
| Drctnnb1a | -2.31 | -2.64 |
| Hdhd2 | -2.4 | -1.68 |
| 4930570C03Rik | -2.36 | -1.75 |
| 1700025G04Rik | -2.43 | -2.92 |
| Trf | -2.51 | -1.94 |
| Atf7ip | -2.44 | -2.91 |
| 6330403K07Rik | -2.5 | -1.98 |
| Reep3 | -2.52 | -2.13 |
| Pon2 | -2.48 | -1.98 |
| Pfdn5 | -2.55 | -2.34 |
| Igsf3 | -2.56 | -1.89 |
| Riok1 | -2.64 | -2.8 |
| AI316807 | -2.65 | -2.22 |
| Pop4 | -2.68 | -2.79 |
| Med23 | -2.7 | -2.59 |
| Gtf3c1 | -2.71 | -1.56 |
| Crym | -2.68 | -1.66 |
| C1qc | -2.77 | -1.76 |
| Atp5g2 | -2.72 | -1.72 |
| Ube2m | -2.86 | -2.3 |
| LOC100048331 | -2.77 | -2.33 |
| C1qb | -2.85 | -2.94 |
| Ddr1 | -2.8 | -2.92 |
| Anxa3 | -2.82 | -1.93 |
| Sv2b | -2.77 | -2.76 |
| Serpine2 | -2.86 | -2.43 |
| Mobp | -2.84 | -2.29 |
| Cmas | -2.86 | -3.84 |
| Tpr | -2.93 | -3.21 |
| Gna13 | -2.95 | -1.52 |
| Zmynd11 | -3.06 | -2.66 |
| Mrpl55 | -3.01 | -2.38 |
| Nefm | -3.11 | -2.42 |
| Rnps1 | -3.14 | -3.64 |
| Mt3 | -3.29 | -2.45 |
| 1190005F20Rik | -3.36 | -2.65 |
| Nudt19 | -3.47 | -3.23 |
| Kcnf1 | -3.56 | -3.23 |
| Tmem68 | -3.67 | -4.75 |
| 5730469M10Rik | -3.76 | -4.22 |
| Kcnh1 | -3.77 | -3.86 |
| Alg1 | -3.85 | -3.47 |
| Cbfa2t3h | -4.28 | -4.24 |
| Acsl6 | -4.31 | -4.95 |
| Rbm28 | -4.83 | -3.68 |
| Rgl1 | -4.81 | -5.75 |
| Hist1h2ao | -4.94 | -4.77 |
| Ccndbp1 | -4.95 | -5.79 |
| Hist1h2af | -5.08 | -5.02 |
| Rapgefl1 | -4.95 | -4.41 |
| Cort | -5.13 | -4.24 |
| 6430706D22Rik | -5.18 | -3.58 |
| Dusp7 | -5.23 | -5.24 |
| Sema5a | -5.24 | -5.09 |
| Bsdc1 | -5.42 | -5.05 |
| BC056474 | -5.68 | -4.04 |
| Gde1 | -5.59 | -6.63 |
| Ehd3 | -5.81 | -4.48 |
| Sdc4 | -5.93 | -5.37 |
| Rbbp9 | -5.97 | -7.14 |
| Psmb5 | -5.98 | -5.09 |
| Slco1c1 | -6.02 | -6.42 |
| Pak1 | -6.01 | -7.87 |
| Ccrn4l | -6.32 | -7.67 |
| Scoc | -6.45 | -7.59 |
| Prdx2 | -6.98 | -7.24 |
| Uap1 | -7.04 | -8.89 |
| Sepw1 | -8.36 | -6.65 |
| Sc4mol | -9.17 | -9.49 |
| Atf4 | -9.48 | -10.14 |
| Enpp5 | -10.93 | -11.79 |
| Hsp90b1 | -12.28 | -12.86 |
| Fgfr1op2 | -12.74 | -12.82 |
| Csrp1 | -17.2 | -17.6 |
|  |  |  |
| Fbxo34 | 2.18 | -1.53 |
| Mrpl3 | 1.7 | -1.96 |
| Rps9p2 | -3.73 | 1.68 |
|  |  |  |
| Lpl | 3.92 |  |
| Rtn4 | 3.08 |  |
| Coq9 | 2.45 |  |
| Wbp11 | 2.36 |  |
| Hn1 | 2.22 |  |
| Kpnb1 | 2.09 |  |
| Igsf1 | 1.99 |  |
| Npcd | 1.95 |  |
| Rerg | 1.98 |  |
| Pole4 | 1.94 |  |
| Ptpn1 | 1.84 |  |
| Hsp90ab1 | 1.96 |  |
| Col16a1 | 1.89 |  |
| Cadm3 | 1.87 |  |
| Ptprs | 1.84 |  |
| Atp9a | 1.84 |  |
| Drbp1 | 1.88 |  |
| Pld3 | 1.87 |  |
| Spock1 | 1.85 |  |
| Zfp30 | 1.83 |  |
| Ecsit | 1.83 |  |
| BC008163 | 1.84 |  |
| Rreb1 | 1.82 |  |
| Tmod2 | 1.83 |  |
| Faim2 | 1.77 |  |
| 4930511J11Rik | 1.75 |  |
| Dcp1b | 1.77 |  |
| Snapc3 | 1.75 |  |
| Serpinf1 | 1.73 |  |
| 6430517E21Rik | 1.74 |  |
| Tomm70a | 1.69 |  |
| 3110035E14Rik | 1.73 |  |
| Shd | 1.7 |  |
| Matk | 1.74 |  |
| Pgam2 | 1.68 |  |
| Cacng5 | 1.63 |  |
| Cxx1a | 1.61 |  |
| Fahd2a | 1.66 |  |
| Tubb2b | 1.69 |  |
| BC026590 | 1.66 |  |
| Tfrc | 1.67 |  |
| 8430415E04Rik | 1.58 |  |
| Adra2c | 1.63 |  |
| Actr3b | 1.57 |  |
| Atp6v0d1 | 1.61 |  |
| Hnrpdl | 1.64 |  |
| Nup133 | 1.6 |  |
| Spire1 | 1.59 |  |
| Camk1d | 1.56 |  |
| Trappc2l | 1.59 |  |
| BC057552 | 1.58 |  |
| St8sia5 | 1.56 |  |
| Napa | 1.58 |  |
| Nt5c | 1.55 |  |
| Ppil3 | 1.56 |  |
| Zdhhc4 | 1.55 |  |
| Zfp612 | 1.53 |  |
| Trpc4 | 1.5 |  |
| Farsb | 1.57 |  |
| Rb1 | 1.51 |  |
|  |  |  |
| Ndrl | -1.5 |  |
| Tspyl3 | -1.51 |  |
| Hist1h2bj | -1.51 |  |
| Sesn1 | -1.51 |  |
| S100a13 | -1.53 |  |
| Fahd1 | -1.6 |  |
| Myadm | -1.54 |  |
| Mras | -1.59 |  |
| Tbcb | -1.55 |  |
| Gtpbp1 | -1.55 |  |
| Nos1ap | -1.52 |  |
| Uhrf1bp1l | -1.58 |  |
| Bex4 | -1.6 |  |
| Myh9 | -1.6 |  |
| Nars | -1.65 |  |
| Igsf4a | -1.58 |  |
| Abhd4 | -1.63 |  |
| 4933407N01Rik | -1.63 |  |
| 1810020D17Rik | -1.61 |  |
| Ddhd1 | -1.64 |  |
| Nr4a2 | -1.65 |  |
| Mrpl9 | -1.64 |  |
| Aprt | -1.65 |  |
| Tsc22d3 | -1.63 |  |
| Cx3cr1 | -1.66 |  |
| Zcchc17 | -1.68 |  |
| Ramp2 | -1.67 |  |
| Ddx24 | -1.69 |  |
| Coro2b | -1.76 |  |
| Mgll | -1.77 |  |
| Dbi | -1.71 |  |
| Purb | -1.76 |  |
| Chn2 | -1.79 |  |
| Armc8 | -1.8 |  |
| Psenen | -1.8 |  |
| Cetn4 | -1.83 |  |
| Fscn1 | -1.89 |  |
| Ly6a | -1.89 |  |
| Lgals1 | -1.85 |  |
| rp9 | -1.91 |  |
| Cnot4 | -1.96 |  |
| 1810030N24Rik | -1.98 |  |
| Ccnd1 | -2 |  |
| Meis2 | -2.11 |  |
| Stk25 | -2.08 |  |
| Amigo2 | -2.1 |  |
| Kcnip3 | -2.18 |  |
| Zc3h13 | -2.14 |  |
| LOC100046741 | -2.02 |  |
| Ndrg3 | -2.12 |  |
| Slc17a6 | -2.31 |  |
| 2610020C11Rik | -2.44 |  |
| Sparc | -2.81 |  |
| Rgs9 | -2.89 |  |
| Napepld | -3.14 |  |
| C1ql2 | -3.69 |  |
| Evc2 | -5.61 |  |
| Hist1h2bm | -5.68 |  |
| 1200015F23Rik | -8.73 |  |
| Spink8 | -17.61 |  |
|  |  |  |
| Pcdh21 |  | 3.97 |
| Rsph1 |  | 3.74 |
| Vars2 |  | 3.64 |
| Nnat |  | 2.89 |
| Gnai2 |  | 2.89 |
| 1200016B10Rik |  | 2.5 |
| Slc6a13 |  | 2.46 |
| Osbpl3 |  | 2.34 |
| Ptgds |  | 2.62 |
| 4930539E08Rik |  | 2.27 |
| B6ip |  | 2.2 |
| Mfsd11 |  | 2.15 |
| Anapc13 |  | 2.31 |
| B930076A02 |  | 2.2 |
| Rab24 |  | 2.14 |
| Ccrk |  | 2.03 |
| Ranbp3l |  | 2.07 |
| Gm129 |  | 1.98 |
| Junb |  | 1.96 |
| Grp |  | 1.79 |
| Prkd3 |  | 1.9 |
| Spag5 |  | 1.8 |
| 1810073G14Rik |  | 1.85 |
| Atp9b |  | 1.8 |
| Map3k3 |  | 1.76 |
| Tmem2 |  | 1.72 |
| Foxn3 |  | 1.71 |
| Stxbp2 |  | 1.69 |
| Tmsb10 |  | 1.81 |
| Mocs1 |  | 1.65 |
| Abhd14b |  | 1.71 |
| Tcea2 |  | 1.74 |
| Gp38 |  | 1.66 |
| Pigt |  | 1.62 |
| EG328644 |  | 1.55 |
| BC021381 |  | 1.53 |
| Srrm2 |  | 1.62 |
| Slc7a10 |  | 1.53 |
| Pcbd2 |  | 1.6 |
| Tnrc6a |  | 1.64 |
| Itgb1bp1 |  | 1.54 |
| LOC100044298 |  | 1.61 |
| Src |  | 1.53 |
| Ebpl |  | 1.53 |
| Akap8l |  | 1.57 |
| Adcy2 |  | 1.62 |
| Psmb10 |  | 1.5 |
| Dbp |  | 1.56 |
| 1500032D16Rik |  | 1.53 |
| Rpn2 |  | 1.53 |
|  |  |  |
| Elavl4 |  | -1.56 |
| Iqgap2 |  | -1.59 |
| Ica1 |  | -1.51 |
| Vip |  | -1.55 |
| Ccdc132 |  | -1.54 |
| Lyrm5 |  | -1.57 |
| Enpp4 |  | -1.59 |
| Mettl3 |  | -1.58 |
| AU022870 |  | -1.57 |
| Med7 |  | -1.58 |
| Mertk |  | -1.63 |
| Ykt6 |  | -1.56 |
| Atg3 |  | -1.74 |
| BC055107 |  | -1.51 |
| Homer1 |  | -1.54 |
| Rpo1-3 |  | -1.58 |
| Bok |  | -1.59 |
| Uchl3 |  | -1.74 |
| Pcdha6 |  | -1.67 |
| Flt1 |  | -1.6 |
| Ppm1l |  | -1.72 |
| Itpka |  | -1.54 |
| Usp33 |  | -1.6 |
| Adar |  | -1.75 |
| Tmem56 |  | -1.66 |
| Dusp14 |  | -1.74 |
| Dmtf1 |  | -1.7 |
| Jdp2 |  | -1.78 |
| Efhd1 |  | -1.78 |
| Enpp6 |  | -1.9 |
| 1500019G21Rik |  | -1.85 |
| Rcan2 |  | -1.7 |
| Darc |  | -1.72 |
| Luzp2 |  | -1.75 |
| Xbp1 |  | -1.66 |
| Nrn1 |  | -1.62 |
| Ap1gbp1 |  | -1.81 |
| Rrm2b |  | -1.88 |
| Yaf2 |  | -1.75 |
| Cpne9 |  | -1.76 |
| Nipsnap1 |  | -1.78 |
| Mat2b |  | -1.86 |
| Trpc7 |  | -2.01 |
| Sema3a |  | -1.99 |
| Ptp4a2 |  | -2.1 |
| Dpp10 |  | -1.91 |
| Lancl1 |  | -1.91 |
| Gabrg2 |  | -2.2 |
| Anln |  | -2.16 |
| Rgs7 |  | -1.99 |
| 1600014C10Rik |  | -2.24 |
| Ppm2c |  | -2.25 |
| Drd1a |  | -2.28 |
| Tnnc1 |  | -2.47 |
| Hspb6 |  | -2.59 |
| Ccdc59 |  | -2.61 |
| Ankrd56 |  | -2.77 |
| Rpp25 |  | -3.56 |
| Myl4 |  | -3.56 |
| Cox6a2 |  | -3.87 |
| LOC100045304 |  | -4.06 |
| Hist1h2an |  | -4.07 |
| 1200003I07Rik |  | -4.3 |
| D14Ertd449e |  | -4.45 |
| Kcnv1 |  | -5.47 |
| Csnrp3 |  | -5.49 |
| Krt12 |  | -8.44 |
