# Supplementary material for: Hippocampal Transcriptomic and Proteomic Alterations in the BTBR Mouse Model of Autism Spectrum Disorder
Source: Front Physiol. 2015 Nov 24;6:324. doi: 10.3389/fphys.2015.00324 (PMC4656818; doi:10.3389/fphys.2015.00324)
Supplement: Supplementary file 5 [file Table4.DOCX]

**Table S4. Canonical Pathway Signaling analysis for transcripts differentially regulated in BTBR hippocampus compared to B6 controls.** Significantly-populated canonical signaling pathways, generated using the transcripts significantly and differentially regulated in BTBR hippocampus compared to B6 controls, are depicted. The pathway enrichment probability (stated as –log_10_(p-value)) and enrichment ratio are stated. The percentages of the total pathway genelist populated by down- (Downregulated) or upregulated (Upregulated) transcripts from the input datasets are also indicated.

| **Canonical Signaling Pathway** | **-log(p-value)** | **Ratio** | **Downregulated** | **Upregulated** |
| --- | --- | --- | --- | --- |
| Glycolysis I | 1.45E+00 | 8.00E-02 | 0/25 (0%) | 2/25 (8%) |
| Gluconeogenesis I | 1.45E+00 | 8.00E-02 | 0/25 (0%) | 2/25 (8%) |
| phagosome maturation | 2.51E+00 | 5.00E-02 | 2/120 (2%) | 4/120 (3%) |
| Neuregulin Signaling | 2.40E+00 | 5.68E-02 | 2/88 (2%) | 3/88 (3%) |
| Virus Entry via Endocytic Pathways | 2.38E+00 | 5.62E-02 | 2/89 (2%) | 3/89 (3%) |
| Glioma Signaling | 2.26E+00 | 5.26E-02 | 2/95 (2%) | 3/95 (3%) |
| Telomerase Signaling | 2.19E+00 | 5.05E-02 | 2/99 (2%) | 3/99 (3%) |
| Pancreatic Adenocarcinoma Signaling | 2.06E+00 | 4.72E-02 | 2/106 (2%) | 3/106 (3%) |
| Epithelial Adherens Junction Signaling | 1.52E+00 | 3.42E-02 | 2/146 (1%) | 3/146 (2%) |
| Germ Cell-Sertoli Cell Junction Signaling | 1.37E+00 | 3.12E-02 | 2/160 (1%) | 3/160 (2%) |
| Melanoma Signaling | 2.82E+00 | 9.52E-02 | 2/42 (5%) | 2/42 (5%) |
| Acute Phase Response Signaling | 2.39E+00 | 4.14E-02 | 3/169 (2%) | 4/169 (2%) |
| IL-8 Signaling | 2.19E+00 | 3.80E-02 | 4/184 (2%) | 3/184 (2%) |
| Non-Small Cell Lung Cancer Signaling | 2.13E+00 | 6.15E-02 | 2/65 (3%) | 2/65 (3%) |
| Choline Biosynthesis III | 2.00E+00 | 1.54E-01 | 1/13 (8%) | 1/13 (8%) |
| PEDF Signaling | 2.00E+00 | 5.63E-02 | 2/71 (3%) | 2/71 (3%) |
| Bladder Cancer Signaling | 1.70E+00 | 4.60E-02 | 2/87 (2%) | 2/87 (2%) |
| PPARα/RXRα Activation | 1.70E+00 | 3.35E-02 | 3/179 (2%) | 3/179 (2%) |
| Chronic Myeloid Leukemia Signaling | 1.61E+00 | 4.30E-02 | 2/93 (2%) | 2/93 (2%) |
| mTOR Signaling | 1.60E+00 | 3.19E-02 | 3/188 (2%) | 3/188 (2%) |
| Breast Cancer Regulation by Stathmin1 | 1.57E+00 | 3.14E-02 | 3/191 (2%) | 3/191 (2%) |
| Estrogen-mediated S-phase Entry | 1.49E+00 | 8.33E-02 | 1/24 (4%) | 1/24 (4%) |
| Triacylglycerol Degradation | 1.49E+00 | 8.33E-02 | 1/24 (4%) | 1/24 (4%) |
| Rac Signaling | 1.45E+00 | 3.85E-02 | 2/104 (2%) | 2/104 (2%) |
| Lipid Antigen Presentation by CD1 | 1.42E+00 | 7.69E-02 | 1/26 (4%) | 1/26 (4%) |
| Prostate Cancer Signaling | 4.27E+00 | 8.54E-02 | 4/82 (5%) | 3/82 (4%) |
| Regulation of Cellular Mechanics by Calpain Protease | 3.24E+00 | 8.77E-02 | 3/57 (5%) | 2/57 (4%) |
| PI3K/AKT Signaling | 2.45E+00 | 4.88E-02 | 4/123 (3%) | 2/123 (2%) |
| PPAR Signaling | 2.28E+00 | 5.32E-02 | 3/94 (3%) | 2/94 (2%) |
| Actin Cytoskeleton Signaling | 1.82E+00 | 3.23E-02 | 4/217 (2%) | 3/217 (1%) |
| Glioblastoma Multiforme Signaling | 1.52E+00 | 3.42E-02 | 3/146 (2%) | 2/146 (1%) |
| ErbB4 Signaling | 1.46E+00 | 5.00E-02 | 2/60 (3%) | 1/60 (2%) |
| GM-CSF Signaling | 1.43E+00 | 4.84E-02 | 2/62 (3%) | 1/62 (2%) |
| Angiopoietin Signaling | 1.36E+00 | 4.55E-02 | 2/66 (3%) | 1/66 (2%) |
| FAK Signaling | 1.70E+00 | 4.60E-02 | 3/87 (3%) | 1/87 (1%) |
| G Beta Gamma Signaling | 1.68E+00 | 4.55E-02 | 3/88 (3%) | 1/88 (1%) |
| Endometrial Cancer Signaling | 1.62E+00 | 5.77E-02 | 2/52 (4%) | 1/52 (2%) |
| ErbB2-ErbB3 Signaling | 1.52E+00 | 5.26E-02 | 2/57 (4%) | 1/57 (2%) |
| Cholecystokinin/Gastrin-mediated Signaling | 1.49E+00 | 3.96E-02 | 3/101 (3%) | 1/101 (1%) |
| HGF Signaling | 1.44E+00 | 3.81E-02 | 3/105 (3%) | 1/105 (1%) |
| Role of Tissue Factor in Cancer | 1.38E+00 | 3.64E-02 | 3/110 (3%) | 1/110 (1%) |
| Macropinocytosis Signaling | 1.33E+00 | 4.41E-02 | 2/68 (3%) | 1/68 (1%) |
| Agrin Interactions at Neuromuscular Junction | 1.31E+00 | 4.35E-02 | 2/69 (3%) | 1/69 (1%) |
| Estrogen-Dependent Breast Cancer Signaling | 2.20E+00 | 6.45E-02 | 3/62 (5%) | 1/62 (2%) |
| ERK5 Signaling | 2.18E+00 | 6.35E-02 | 3/63 (5%) | 1/63 (2%) |
| Hypoxia Signaling in the Cardiovascular System | 2.13E+00 | 6.15E-02 | 3/65 (5%) | 1/65 (2%) |
| Cell Cycle Regulation by BTG Family Proteins | 2.09E+00 | 8.57E-02 | 2/35 (6%) | 1/35 (3%) |
| Neurotrophin/TRK Signaling | 2.08E+00 | 5.97E-02 | 3/67 (4%) | 1/67 (1%) |
| Ephrin B Signaling | 1.95E+00 | 5.48E-02 | 3/73 (4%) | 1/73 (1%) |
| HER-2 Signaling in Breast Cancer | 1.90E+00 | 5.26E-02 | 3/76 (4%) | 1/76 (1%) |
| Thyroid Cancer Signaling | 2.90E+00 | 1.00E-01 | 3/40 (8%) | 1/40 (3%) |
| Endoplasmic Reticulum Stress Pathway | 1.60E+00 | 9.52E-02 | 2/21 (10%) | 0/21 (0%) |
