# Supplementary material for: Hippocampal Transcriptomic and Proteomic Alterations in the BTBR Mouse Model of Autism Spectrum Disorder
Source: Front Physiol. 2015 Nov 24;6:324. doi: 10.3389/fphys.2015.00324 (PMC4656818; doi:10.3389/fphys.2015.00324)
Supplement: Supplementary file 6 [file Table5.DOCX]

**Table S5. Canonical Pathway Signaling analysis for transcripts differentially regulated in BTBR cortex compared to B6 controls.** Significantly-populated canonical signaling pathways, generated using the transcripts significantly and differentially regulated in BTBR cortex compared to B6 controls, are depicted. The pathway enrichment probability (stated as –log_10_(p-value)) and enrichment ratio are stated. The percentages of the total pathway genelist populated by down- (Downregulated) or upregulated (Upregulated) transcripts from the input datasets are also indicated.

| **Canonical Signaling Pathway** | **-log(p-value)** | **Ratio** | **Downregulated** | **Upregulated** |
| --- | --- | --- | --- | --- |
| UVC-Induced MAPK Signaling | 1.91E+00 | 7.14E-02 | 0/42 (0%) | 3/42 (7%) |
| α-Adrenergic Signaling | 1.75E+00 | 4.60E-02 | 0/87 (0%) | 4/87 (5%) |
| G Beta Gamma Signaling | 4.16E+00 | 7.95E-02 | 2/88 (2%) | 5/88 (6%) |
| Erythropoietin Signaling | 1.38E+00 | 4.48E-02 | 0/67 (0%) | 3/67 (4%) |
| Chemokine Signaling | 1.32E+00 | 4.23E-02 | 0/71 (0%) | 3/71 (4%) |
| GNRH Signaling | 3.91E+00 | 6.20E-02 | 2/129 (2%) | 6/129 (5%) |
| Virus Entry via Endocytic Pathways | 2.44E+00 | 5.62E-02 | 1/89 (1%) | 4/89 (4%) |
| GABA Receptor Signaling | 2.13E+00 | 5.97E-02 | 1/67 (1%) | 3/67 (4%) |
| Macropinocytosis Signaling | 2.11E+00 | 5.88E-02 | 1/68 (1%) | 3/68 (4%) |
| GPCR-Mediated Integration of Enteroendocrine Signaling Exemplified by an L Cell | 2.05E+00 | 5.63E-02 | 1/71 (1%) | 3/71 (4%) |
| Melanocyte Development and Pigmentation Signaling | 1.80E+00 | 4.76E-02 | 1/84 (1%) | 3/84 (4%) |
| Gap Junction Signaling | 1.48E+00 | 3.23E-02 | 0/155 (0%) | 5/155 (3%) |
| ERK5 Signaling | 3.11E+00 | 7.94E-02 | 2/63 (3%) | 3/63 (5%) |
| CREB Signaling in Neurons | 2.44E+00 | 4.09E-02 | 2/171 (1%) | 5/171 (3%) |
| IL-8 Signaling | 2.27E+00 | 3.80E-02 | 2/184 (1%) | 5/184 (3%) |
| Synaptic Long Term Depression | 2.22E+00 | 4.23E-02 | 2/142 (1%) | 4/142 (3%) |
| Thrombin Signaling | 2.18E+00 | 3.66E-02 | 2/191 (1%) | 5/191 (3%) |
| p70S6K Signaling | 1.92E+00 | 4.20E-02 | 1/119 (1%) | 4/119 (3%) |
| P2Y Purigenic Receptor Signaling Pathway | 1.92E+00 | 4.20E-02 | 1/119 (1%) | 4/119 (3%) |
| Gαi Signaling | 1.90E+00 | 4.17E-02 | 1/120 (1%) | 4/120 (3%) |
| Endothelin-1 Signaling | 1.84E+00 | 3.49E-02 | 1/172 (1%) | 5/172 (3%) |
| Role of NFAT in Cardiac Hypertrophy | 1.76E+00 | 3.35E-02 | 1/179 (1%) | 5/179 (3%) |
| Neuregulin Signaling | 1.73E+00 | 4.55E-02 | 1/88 (1%) | 3/88 (3%) |
| Regulation of Cellular Mechanics by Calpain Protease | 1.56E+00 | 5.26E-02 | 1/57 (2%) | 2/57 (4%) |
| Neuropathic Pain Signaling In Dorsal Horn Neurons | 1.55E+00 | 4.00E-02 | 1/100 (1%) | 3/100 (3%) |
| HGF Signaling | 1.48E+00 | 3.81E-02 | 1/105 (1%) | 3/105 (3%) |
| Renin-Angiotensin Signaling | 1.43E+00 | 3.67E-02 | 1/109 (1%) | 3/109 (3%) |
| Germ Cell-Sertoli Cell Junction Signaling | 1.43E+00 | 3.12E-02 | 1/160 (1%) | 4/160 (3%) |
| Androgen Signaling | 1.41E+00 | 3.60E-02 | 1/111 (1%) | 3/111 (3%) |
| Agrin Interactions at Neuromuscular Junction | 1.35E+00 | 4.35E-02 | 1/69 (1%) | 2/69 (3%) |
| 14-3-3-mediated Signaling | 1.34E+00 | 3.42E-02 | 1/117 (1%) | 3/117 (3%) |
| CCR3 Signaling in Eosinophils | 1.34E+00 | 3.42E-02 | 1/117 (1%) | 3/117 (3%) |
| IL-3 Signaling | 1.32E+00 | 4.23E-02 | 1/71 (1%) | 2/71 (3%) |
| Synaptic Long Term Potentiation | 1.32E+00 | 3.36E-02 | 1/119 (1%) | 3/119 (3%) |
| CXCR4 Signaling | 3.42E+00 | 5.26E-02 | 3/152 (2%) | 5/152 (3%) |
| Cholecystokinin/Gastrin-mediated Signaling | 2.21E+00 | 4.95E-02 | 2/101 (2%) | 3/101 (3%) |
| Corticotropin Releasing Hormone Signaling | 2.04E+00 | 4.50E-02 | 2/111 (2%) | 3/111 (3%) |
| Phospholipase C Signaling | 1.69E+00 | 2.93E-02 | 3/239 (1%) | 4/239 (2%) |
| G-Protein Coupled Receptor Signaling | 1.54E+00 | 2.73E-02 | 3/256 (1%) | 4/256 (2%) |
| Estrogen-Dependent Breast Cancer Signaling | 1.46E+00 | 4.84E-02 | 1/62 (2%) | 2/62 (3%) |
| Tec Kinase Signaling | 1.45E+00 | 3.16E-02 | 2/158 (1%) | 3/158 (2%) |
| Dopamine-DARPP32 Feedback in cAMP Signaling | 1.42E+00 | 3.11E-02 | 2/161 (1%) | 3/161 (2%) |
| Cardiac Hypertrophy Signaling | 1.36E+00 | 2.69E-02 | 2/223 (1%) | 4/223 (2%) |
| Breast Cancer Regulation by Stathmin1 | 2.18E+00 | 3.66E-02 | 3/191 (2%) | 4/191 (2%) |
| Ephrin B Signaling | 2.00E+00 | 5.48E-02 | 2/73 (3%) | 2/73 (3%) |
| STAT3 Pathway | 2.00E+00 | 5.48E-02 | 2/73 (3%) | 2/73 (3%) |
| Ephrin Receptor Signaling | 1.81E+00 | 3.45E-02 | 3/174 (2%) | 3/174 (2%) |
| FAK Signaling | 1.75E+00 | 4.60E-02 | 2/87 (2%) | 2/87 (2%) |
| CDK5 Signaling | 1.56E+00 | 4.04E-02 | 2/99 (2%) | 2/99 (2%) |
| Rac Signaling | 1.50E+00 | 3.85E-02 | 2/104 (2%) | 2/104 (2%) |
| Lipid Antigen Presentation by CD1 | 1.45E+00 | 7.69E-02 | 1/26 (4%) | 1/26 (4%) |
| Role of Tissue Factor in Cancer | 1.42E+00 | 3.64E-02 | 2/110 (2%) | 2/110 (2%) |
| cAMP-mediated signaling | 1.39E+00 | 2.74E-02 | 3/219 (1%) | 3/219 (1%) |
| Gα12/13 Signaling | 1.34E+00 | 3.42E-02 | 2/117 (2%) | 2/117 (2%) |
| Cdc42 Signaling | 1.89E+00 | 3.59E-02 | 4/167 (2%) | 2/167 (1%) |
| Actin Cytoskeleton Signaling | 1.40E+00 | 2.76E-02 | 4/217 (2%) | 2/217 (1%) |
| Mitotic Roles of Polo-Like Kinase | 1.40E+00 | 4.55E-02 | 2/66 (3%) | 1/66 (2%) |
| RhoGDI Signaling | 1.31E+00 | 2.89E-02 | 3/173 (2%) | 2/173 (1%) |
| NF-κB Signaling | 1.31E+00 | 2.89E-02 | 3/173 (2%) | 2/173 (1%) |
| Protein Ubiquitination Pathway | 2.55E+00 | 3.53E-02 | 7/255 (3%) | 2/255 (1%) |
| Calcium Signaling | 1.77E+00 | 3.37E-02 | 5/178 (3%) | 1/178 (1%) |
| Neurotrophin/TRK Signaling | 1.38E+00 | 4.48E-02 | 2/67 (3%) | 1/67 (1%) |
| phagosome maturation | 1.31E+00 | 3.33E-02 | 3/120 (3%) | 1/120 (1%) |
| Hypoxia Signaling in the Cardiovascular System | 1.41E+00 | 4.62E-02 | 3/65 (5%) | 0/65 (0%) |
| Unfolded protein response | 1.62E+00 | 5.56E-02 | 3/54 (6%) | 0/54 (0%) |
| Endoplasmic Reticulum Stress Pathway | 2.77E+00 | 1.43E-01 | 3/21 (14%) | 0/21 (0%) |
