# Supplementary material for: Hippocampal Transcriptomic and Proteomic Alterations in the BTBR Mouse Model of Autism Spectrum Disorder
Source: Front Physiol. 2015 Nov 24;6:324. doi: 10.3389/fphys.2015.00324 (PMC4656818; doi:10.3389/fphys.2015.00324)
Supplement: Supplementary file 7 [file Table6.DOCX]

**Table S6. KEGG pathway analysis for transcripts differentially regulated in BTBR hippocampus compared to B6 controls.** Significantly-populated KEGG signaling pathways, generated using the transcripts significantly and differentially regulated in BTBR hippocampus compared to B6 controls, are depicted. Before KEGG pathway annotation, significant and differentially-regulated transcripts in the BTBR mice were separated into upregulated or downregulated (compared to B6 controls) lists. KEGG pathways generated from the upregulated transcript list are indicated in the KEGG Pathway – UPREGULATED set, while those pathways populated by the downregulated transcripts are indicated in the KEGG Pathway – DOWNREGULATED sets. For each specific KEGG pathway annotation the following parameter indices are indicated: **C** - total background number of transcripts populating the KEGG pathway; **O** – number of observed transcripts within the input dataset that are contained within the specific KEGG pathway; **E** – number of transcripts from the input dataset expected to be present at background levels; **R** – transcript enrichment factor in specific KEGG pathway, **P** – enrichment probability; **H** – hybrid score = -log_10_P * R.

| **KEGG Pathway - UPREGULATED** | **C** | **O** | **E** | **R** | **P** | **H** |
| --- | --- | --- | --- | --- | --- | --- |
| Metabolic pathways | 1184 | 10 | 2.29 | 4.36 | 0.0031 | 10.937663 |
| Dorso-ventral axis formation | 22 | 2 | 0.04 | 46.97 | 0.0083 | 97.740902 |
| Prostate cancer | 89 | 3 | 0.17 | 17.41 | 0.0083 | 36.22885 |
| Adherens junction | 75 | 2 | 0.15 | 13.78 | 0.0157 | 24.860503 |
| Long-term potentiation | 69 | 2 | 0.13 | 14.97 | 0.0157 | 27.007382 |
| Purine metabolism | 168 | 3 | 0.33 | 9.23 | 0.0157 | 16.651846 |
| Glioma | 66 | 2 | 0.13 | 15.66 | 0.0157 | 28.252211 |
| Phagosome | 176 | 3 | 0.34 | 8.81 | 0.0157 | 15.894124 |
| Melanoma | 72 | 2 | 0.14 | 14.35 | 0.0157 | 25.88884 |
| Bladder cancer | 43 | 2 | 0.08 | 24.03 | 0.0157 | 43.352531 |
| Huntington's disease | 197 | 3 | 0.38 | 7.87 | 0.0157 | 14.19827 |
| Non-small cell lung cancer | 55 | 2 | 0.11 | 18.79 | 0.0157 | 33.899046 |
| Long-term depression | 72 | 2 | 0.14 | 14.35 | 0.0157 | 25.88884 |
| Protein processing in endoplasmic reticulum | 169 | 3 | 0.33 | 9.17 | 0.0157 | 16.5436 |
| RNA degradation | 76 | 2 | 0.15 | 13.6 | 0.0157 | 24.535765 |
| Regulation of actin cytoskeleton | 216 | 3 | 0.42 | 7.18 | 0.0157 | 12.95344 |
| Pancreatic cancer | 71 | 2 | 0.14 | 14.55 | 0.0157 | 26.24966 |
| Chronic myeloid leukemia | 74 | 2 | 0.14 | 13.96 | 0.0157 | 25.185241 |
| Glycolysis / Gluconeogenesis | 62 | 2 | 0.12 | 16.67 | 0.0157 | 30.074353 |
| Progesterone-mediated oocyte maturation | 88 | 2 | 0.17 | 11.74 | 0.0189 | 20.234338 |
| Gap junction | 88 | 2 | 0.17 | 11.74 | 0.0189 | 20.234338 |
| MAPK signaling pathway | 268 | 3 | 0.52 | 5.78 | 0.0214 | 9.6502084 |
| Pyrimidine metabolism | 99 | 2 | 0.19 | 10.44 | 0.0214 | 17.43048 |
| Pathways in cancer | 325 | 3 | 0.63 | 4.77 | 0.0329 | 7.0729756 |
| Insulin signaling pathway | 137 | 2 | 0.27 | 7.54 | 0.0362 | 10.867337 |
| Oxidative phosphorylation | 147 | 2 | 0.28 | 7.03 | 0.0386 | 9.9362912 |
| Parkinson's disease | 148 | 2 | 0.29 | 6.98 | 0.0386 | 9.8656206 |
| RNA transport | 168 | 2 | 0.33 | 6.15 | 0.0468 | 8.177988 |
|  |  |  |  |  |  |  |
| **KEGG Pathway - DOWNREGULATED** | **C** | **O** | **E** | **R** | **P** | **H** |
| Systemic lupus erythematosus | 149 | 8 | 0.41 | 19.42 | 3.40E-07 | 125.61868 |
| Ribosome biogenesis in eukaryotes | 86 | 4 | 0.24 | 16.82 | 0.0016 | 47.026702 |
| Metabolic pathways | 1184 | 11 | 3.27 | 3.36 | 0.0055 | 7.5923814 |
| p53 signaling pathway | 70 | 3 | 0.19 | 15.5 | 0.0066 | 33.797069 |
| MAPK signaling pathway | 268 | 5 | 0.74 | 6.75 | 0.0066 | 14.718078 |
| Prostate cancer | 89 | 3 | 0.25 | 12.19 | 0.011 | 23.875423 |
| Thyroid cancer | 30 | 2 | 0.08 | 24.11 | 0.0132 | 45.312963 |
| Regulation of actin cytoskeleton | 216 | 4 | 0.6 | 6.7 | 0.0132 | 12.592155 |
| Prion diseases | 35 | 2 | 0.1 | 20.66 | 0.0158 | 37.215745 |
| Amino sugar and nucleotide sugar metabolism | 48 | 2 | 0.13 | 15.07 | 0.0234 | 24.575917 |
| Staphylococcus aureus infection | 50 | 2 | 0.14 | 14.47 | 0.0234 | 23.597447 |
| N-Glycan biosynthesis | 50 | 2 | 0.14 | 14.47 | 0.0234 | 23.597447 |
| Protein processing in endoplasmic reticulum | 169 | 3 | 0.47 | 6.42 | 0.0276 | 10.009364 |
| RNA transport | 168 | 3 | 0.46 | 6.46 | 0.0276 | 10.071727 |
| PPAR signaling pathway | 80 | 2 | 0.22 | 9.04 | 0.0406 | 12.578925 |
| Peroxisome | 80 | 2 | 0.22 | 9.04 | 0.0406 | 12.578925 |
| Complement and coagulation cascades | 76 | 2 | 0.21 | 9.52 | 0.0406 | 13.246832 |
| ECM-receptor interaction | 86 | 2 | 0.24 | 8.41 | 0.0438 | 11.425203 |
| Viral myocarditis | 89 | 2 | 0.25 | 8.13 | 0.0441 | 11.020714 |
