# Supplementary material for: Hippocampal Transcriptomic and Proteomic Alterations in the BTBR Mouse Model of Autism Spectrum Disorder
Source: Front Physiol. 2015 Nov 24;6:324. doi: 10.3389/fphys.2015.00324 (PMC4656818; doi:10.3389/fphys.2015.00324)
Supplement: Supplementary file 8 [file Table7.DOCX]

**Table S7. KEGG pathway analysis for transcripts differentially regulated in BTBR cortex compared to B6 controls.** Significantly-populated KEGG signaling pathways, generated using the transcripts significantly and differentially regulated in BTBR cortex compared to B6 controls, are depicted. Before KEGG pathway annotation, significant and differentially-regulated transcripts in the BTBR mice were separated into upregulated or downregulated (compared to B6 controls) lists. KEGG pathways generated from the upregulated transcript list are indicated in the KEGG Pathway – UPREGULATED set, while those pathways populated by the downregulated transcripts are indicated in the KEGG Pathway – DOWNREGULATED sets. For each specific KEGG pathway annotation the following parameter indices are indicated: **C** - total background number of transcripts populating the KEGG pathway; **O** – number of observed transcripts within the input dataset that are contained within the specific KEGG pathway; **E** – number of transcripts from the input dataset expected to be present at background levels; **R** – transcript enrichment factor in specific KEGG pathway, **P** – enrichment probability; **H** – hybrid score = -log_10_P * R.

| **KEGG Pathway - UPREGULATED** | **C** | **O** | **E** | **R** | **P** | **H** |
| --- | --- | --- | --- | --- | --- | --- |
| Progesterone-mediated oocyte maturation | 88 | 4 | 0.15 | 27.4 | 0.0002 | 101.3518 |
| Gap junction | 88 | 4 | 0.15 | 27.4 | 0.0002 | 101.3518 |
| GnRH signaling pathway | 99 | 4 | 0.16 | 24.35 | 0.0002 | 90.06992 |
| Long-term depression | 72 | 3 | 0.12 | 25.11 | 0.0014 | 71.66073 |
| Melanogenesis | 100 | 3 | 0.17 | 18.08 | 0.0032 | 45.10689 |
| Metabolic pathways | 1184 | 8 | 1.96 | 4.07 | 0.0036 | 9.945849 |
| Tight junction | 137 | 3 | 0.23 | 13.2 | 0.0062 | 29.14043 |
| Protein processing in endoplasmic reticulum | 169 | 3 | 0.28 | 10.7 | 0.0098 | 21.49388 |
| Chemokine signaling pathway | 185 | 3 | 0.31 | 9.77 | 0.0111 | 19.09719 |
| Huntington's disease | 197 | 3 | 0.33 | 9.18 | 0.0119 | 17.66648 |
| Gastric acid secretion | 73 | 2 | 0.12 | 16.51 | 0.013 | 31.1388 |
| Adherens junction | 75 | 2 | 0.12 | 16.07 | 0.013 | 30.30893 |
| Regulation of actin cytoskeleton | 216 | 3 | 0.36 | 8.37 | 0.013 | 15.78629 |
| VEGF signaling pathway | 76 | 2 | 0.13 | 15.86 | 0.013 | 29.91286 |
| Long-term potentiation | 69 | 2 | 0.11 | 17.47 | 0.013 | 32.94941 |
| ErbB signaling pathway | 87 | 2 | 0.14 | 13.86 | 0.0157 | 25.00483 |
| Oocyte meiosis | 113 | 2 | 0.19 | 10.67 | 0.0243 | 17.22558 |
| Leukocyte transendothelial migration | 120 | 2 | 0.2 | 10.05 | 0.0256 | 15.99719 |
| Axon guidance | 131 | 2 | 0.22 | 9.2 | 0.0273 | 14.3873 |
| Neurotrophin signaling pathway | 131 | 2 | 0.22 | 9.2 | 0.0273 | 14.3873 |
| Ubiquitin mediated proteolysis | 140 | 2 | 0.23 | 8.61 | 0.0294 | 13.18753 |
| Parkinson's disease | 148 | 2 | 0.25 | 8.14 | 0.0311 | 12.26893 |
| Purine metabolism | 168 | 2 | 0.28 | 7.18 | 0.0376 | 10.23015 |
| Calcium signaling pathway | 178 | 2 | 0.3 | 6.77 | 0.0399 | 9.471413 |
| Focal adhesion | 200 | 2 | 0.33 | 6.03 | 0.0474 | 7.985057 |
|  |  |  |  |  |  |  |
| **KEGG Pathway - DOWNREGULATED** | **C** | **O** | **E** | **R** | **P** | **H** |
| Systemic lupus erythematosus | 149 | 7 | 0.42 | 16.47 | 9.42E-06 | 82.77738 |
| Ribosome biogenesis in eukaryotes | 86 | 5 | 0.25 | 20.39 | 8.98E-05 | 82.5127 |
| Metabolic pathways | 1184 | 13 | 3.38 | 3.85 | 0.0005 | 12.70897 |
| RNA transport | 168 | 4 | 0.48 | 8.35 | 0.0088 | 17.16357 |
| Protein processing in endoplasmic reticulum | 169 | 4 | 0.48 | 8.3 | 0.0088 | 17.06079 |
| MAPK signaling pathway | 268 | 5 | 0.76 | 6.54 | 0.0088 | 13.44308 |
| Prion diseases | 35 | 2 | 0.1 | 20.04 | 0.0225 | 33.02226 |
| N-Glycan biosynthesis | 50 | 2 | 0.14 | 14.03 | 0.0265 | 22.12186 |
| Axon guidance | 131 | 3 | 0.37 | 8.03 | 0.0265 | 12.66134 |
| Amino sugar and nucleotide sugar metabolism | 48 | 2 | 0.14 | 14.61 | 0.0265 | 23.03638 |
| Staphylococcus aureus infection | 50 | 2 | 0.14 | 14.03 | 0.0265 | 22.12186 |
| Malaria | 46 | 2 | 0.13 | 15.25 | 0.0265 | 24.0455 |
| Cytosolic DNA-sensing pathway | 56 | 2 | 0.16 | 12.52 | 0.0304 | 18.99442 |
| Calcium signaling pathway | 178 | 3 | 0.51 | 5.91 | 0.0365 | 8.496849 |
| p53 signaling pathway | 70 | 2 | 0.2 | 10.02 | 0.0401 | 13.99649 |
| Endocytosis | 220 | 3 | 0.63 | 4.78 | 0.0404 | 6.661497 |
| Huntington's disease | 197 | 3 | 0.56 | 5.34 | 0.0404 | 7.441924 |
| Cardiac muscle contraction | 81 | 2 | 0.23 | 8.66 | 0.0404 | 12.06874 |
| Complement and coagulation cascades | 76 | 2 | 0.22 | 9.23 | 0.0404 | 12.8631 |
| Peroxisome | 80 | 2 | 0.23 | 8.77 | 0.0404 | 12.22204 |
| ECM-receptor interaction | 86 | 2 | 0.25 | 8.16 | 0.0404 | 11.37193 |
| Regulation of actin cytoskeleton | 216 | 3 | 0.62 | 4.87 | 0.0404 | 6.786923 |
| Prostate cancer | 89 | 2 | 0.25 | 7.88 | 0.0409 | 10.93962 |
| Pyrimidine metabolism | 99 | 2 | 0.28 | 7.08 | 0.0466 | 9.427828 |
| Chagas disease (American trypanosomiasis) | 100 | 2 | 0.29 | 7.01 | 0.0466 | 9.334615 |
