# Supplementary material for: Hippocampal Transcriptomic and Proteomic Alterations in the BTBR Mouse Model of Autism Spectrum Disorder
Source: Front Physiol. 2015 Nov 24;6:324. doi: 10.3389/fphys.2015.00324 (PMC4656818; doi:10.3389/fphys.2015.00324)
Supplement: Supplementary file 9 [file Table8.DOCX]

**Table S8. *Textrous!* Collective Processing Hierarchical Cloud data output from BTBR hippocampal transcriptomic data**. For each derived noun the Cosine Similarity score, Z-score and p-Value are indicated.

| **Word** | **Cosine Similarity** | **Z-score** | **p-Value** |
| --- | --- | --- | --- |
| brain-derived | 0.640852783 | 3.196866694 | 0.000694325 |
| neurotrophic | 0.638805031 | 3.186767815 | 0.000718784 |
| neuroplasticity | 0.620021034 | 3.094130982 | 0.000987387 |
| plasticity | 0.550558889 | 2.751565277 | 0.002961626 |
| depression | 0.526884095 | 2.634808551 | 0.004206864 |
| hippocampal | 0.525202534 | 2.626515617 | 0.004307067 |
| neurotrophins | 0.481548795 | 2.411228944 | 0.007954424 |
| retinoblastoma | 0.45725863 | 2.291437402 | 0.010981707 |
| rb | 0.453697776 | 2.273876375 | 0.011482993 |
| potentiation | 0.434363602 | 2.178526239 | 0.014665834 |
| episodic | 0.428308863 | 2.148666151 | 0.0158172 |
| learning | 0.425861013 | 2.136594115 | 0.016298996 |
| long-term | 0.425342251 | 2.134035741 | 0.01642139 |
| huntington's | 0.417535901 | 2.09553725 | 0.018041095 |
| synapses | 0.405089513 | 2.034155531 | 0.020975798 |
| synaptic | 0.401986672 | 2.018853284 | 0.02174361 |
| hippocampus | 0.401583349 | 2.01686422 | 0.021847757 |
| transmissions | 0.400863122 | 2.013312287 | 0.022057315 |
| postsynaptic | 0.400837511 | 2.013185982 | 0.022057315 |
| neostriatum | 0.397891005 | 1.998654729 | 0.022804177 |
| stressful | 0.395678426 | 1.987742979 | 0.023405845 |
| transmission | 0.376708014 | 1.894186801 | 0.029112505 |
| peristaltic | 0.376498205 | 1.893152092 | 0.029178935 |
| vestibular | 0.372809011 | 1.874958131 | 0.030396362 |
| glutamatergic | 0.366523921 | 1.843962021 | 0.03259157 |
| ampa | 0.360973352 | 1.816588342 | 0.034608557 |
| n-methyl-d-aspartic | 0.360088173 | 1.812222915 | 0.034993098 |
| miniature | 0.357666029 | 1.800277653 | 0.035930319 |
| pdz | 0.346477382 | 1.745098721 | 0.040492434 |
| somatosensory | 0.344971549 | 1.737672424 | 0.041105407 |
| axonemes | 0.344008186 | 1.732921416 | 0.041547834 |
| excitatory | 0.340575192 | 1.715990957 | 0.043081021 |
| nmda | 0.340403572 | 1.715144581 | 0.043172613 |
| hcs | 0.335712281 | 1.69200859 | 0.045322987 |
| extinction | 0.334898031 | 1.687992964 | 0.045705614 |
| nmdar | 0.334340346 | 1.685242632 | 0.045994286 |
| n-ras | 0.334142527 | 1.68426705 | 0.046090834 |
| bipolar | 0.33329838 | 1.680103981 | 0.046478658 |
| enucleation | 0.332890139 | 1.678090662 | 0.04667355 |
| presynaptic | 0.331539443 | 1.67142945 | 0.047360839 |
| gustatory | 0.328056544 | 1.654252876 | 0.049063756 |
| n-methyl-d-aspartate | 0.327388626 | 1.650958912 | 0.049369287 |
| depressed | 0.326272057 | 1.645452342 | 0.049984906 |
| innervation | 0.323449104 | 1.631530413 | 0.051339743 |
| eating | 0.323353503 | 1.631058942 | 0.05144516 |
| mechanoreceptor | 0.322151658 | 1.625131816 | 0.052081279 |
| post-synaptic | 0.319726 | 1.613169225 | 0.053372254 |
| edwards | 0.317491242 | 1.602148088 | 0.054577805 |
| ionotropic | 0.31638255 | 1.596680367 | 0.055132854 |
| pentylenetetrazol | 0.316106215 | 1.595317566 | 0.055356118 |
| morris | 0.315592337 | 1.592783281 | 0.055580096 |
| gabaergic | 0.315289881 | 1.591291664 | 0.055804788 |
| propionic | 0.311049699 | 1.570380404 | 0.058207556 |
| victims | 0.305034222 | 1.540713939 | 0.061658393 |
| high-frequency | 0.303578286 | 1.533533715 | 0.062514826 |
| kainic | 0.297416482 | 1.503145613 | 0.066419522 |
| piriform | 0.295973784 | 1.496030676 | 0.067326828 |
| cingulate | 0.294386764 | 1.488203988 | 0.068375447 |
| terminals | 0.291394816 | 1.473448632 | 0.070375518 |
| line-derived | 0.289461775 | 1.463915474 | 0.071596974 |
| gyrus | 0.289401182 | 1.463616645 | 0.071596974 |
| geniculate | 0.286581753 | 1.4497121 | 0.07352926 |
| dendrites | 0.28426594 | 1.43829123 | 0.075217027 |
| pyramidal | 0.282185352 | 1.428030416 | 0.076645929 |
| training | 0.28053969 | 1.419914522 | 0.077803841 |
| memories | 0.275708667 | 1.396089419 | 0.081357248 |
| papillae | 0.275068453 | 1.392932081 | 0.081809895 |
| maze | 0.273786761 | 1.386611174 | 0.082720881 |
| relapsing-remitting | 0.270656421 | 1.371173314 | 0.085187478 |
| axoneme | 0.27049005 | 1.370352822 | 0.085343451 |
| transmitter | 0.270018735 | 1.368028442 | 0.085656038 |
| optokinetic | 0.269736111 | 1.366634627 | 0.085812653 |
| fear | 0.2695706 | 1.365818382 | 0.085969482 |
| utricle | 0.26920289 | 1.364004947 | 0.086283783 |
| beta-tubulin | 0.266573434 | 1.351037289 | 0.088347716 |
| metallochaperone | 0.266545895 | 1.350901477 | 0.088347716 |
| saccule | 0.266017046 | 1.348293356 | 0.088829191 |
| metabotropic | 0.266004824 | 1.348233081 | 0.088829191 |
| glutamate | 0.264825546 | 1.342417249 | 0.089797998 |
| associative | 0.264312737 | 1.339888235 | 0.090122672 |
| neurotrophin | 0.262762473 | 1.332242812 | 0.091430094 |
| short-term | 0.262369736 | 1.330305957 | 0.091759136 |
| hippocampi | 0.262101988 | 1.328985508 | 0.091923985 |
| small-cell | 0.261621257 | 1.326614694 | 0.092254341 |
| kainate | 0.261418118 | 1.325612873 | 0.092419849 |
| spines | 0.258848388 | 1.312939763 | 0.094591474 |
| monocular | 0.257468087 | 1.306132546 | 0.095776279 |
| electroconvulsive | 0.257170913 | 1.304666977 | 0.095946424 |
| naturally-occurring | 0.257109502 | 1.304364117 | 0.096116791 |
| anchorage | 0.25647037 | 1.301212117 | 0.096629227 |
| phospho | 0.256372595 | 1.300729922 | 0.096629227 |
| hypofunction | 0.25611472 | 1.299458161 | 0.096971965 |
| experiences | 0.25541956 | 1.296029851 | 0.097487742 |
| dentate | 0.253075239 | 1.284468386 | 0.099570978 |
| memory | 0.252510211 | 1.281681847 | 0.099921323 |
| spikes | 0.252326643 | 1.280776547 | 0.100096833 |
| occipital | 0.252164888 | 1.279978818 | 0.100272568 |
| p-type | 0.251273085 | 1.275580722 | 0.10097776 |
| deafferentation | 0.248324057 | 1.261037033 | 0.103654424 |
| alpha-tubulin | 0.248206254 | 1.260456065 | 0.103834681 |
