# Supplementary material for: Hippocampal Transcriptomic and Proteomic Alterations in the BTBR Mouse Model of Autism Spectrum Disorder
Source: Front Physiol. 2015 Nov 24;6:324. doi: 10.3389/fphys.2015.00324 (PMC4656818; doi:10.3389/fphys.2015.00324)
Supplement: Supplementary file 10 [file Table9.DOCX]

**Table S9. *Textrous!* Collective Processing Hierarchical Cloud data output from BTBR cortical transcriptomic data**. For each derived noun the Cosine Similarity score, Z-score and p-Value are indicated.

| **Word** | **Cosine Similarity** | **Z-score** | **p-Value** |
| --- | --- | --- | --- |
| neuroplasticity | 0.60277599 | 2.778278971 | 0.002734731 |
| plasticity | 0.598258898 | 2.757591426 | 0.00290781 |
| brain-derived | 0.588590485 | 2.713311675 | 0.003333855 |
| neurotrophic | 0.582445449 | 2.685168412 | 0.00362649 |
| cytokeratin | 0.557976822 | 2.573106094 | 0.00504106 |
| keratins | 0.556487405 | 2.566284804 | 0.005143941 |
| keratin | 0.555088513 | 2.55987811 | 0.005233608 |
| cytokeratins | 0.551212556 | 2.542126856 | 0.005511009 |
| potentiation | 0.528092067 | 2.43623879 | 0.007425342 |
| learning | 0.517322541 | 2.38691612 | 0.008493246 |
| hippocampal | 0.516879545 | 2.384887271 | 0.008539561 |
| hyperkeratosis | 0.516813661 | 2.384585531 | 0.008539561 |
| keratoderma | 0.50541584 | 2.33238537 | 0.009850346 |
| palmoplantar | 0.49663482 | 2.292169734 | 0.010952822 |
| catalog | 0.49648861 | 2.291500114 | 0.010952822 |
| nail | 0.495525548 | 2.287089448 | 0.011097912 |
| long-term | 0.492049697 | 2.271170618 | 0.011573489 |
| depression | 0.49162223 | 2.269212887 | 0.011634163 |
| clumping | 0.47910901 | 2.211904382 | 0.013483332 |
| soles | 0.47714308 | 2.202900742 | 0.013797374 |
| transmission | 0.4652226 | 2.148306888 | 0.015856878 |
| postsynaptic | 0.449392892 | 2.07580941 | 0.018946975 |
| extinction | 0.449198747 | 2.074920255 | 0.018993267 |
| keratosis | 0.448242151 | 2.070539203 | 0.019179398 |
| ceramides | 0.441179904 | 2.038195267 | 0.020774966 |
| huntington's | 0.439103538 | 2.028685848 | 0.021229145 |
| neurotrophins | 0.438693779 | 2.026809216 | 0.021331206 |
| associative | 0.437084934 | 2.019440971 | 0.02174361 |
| nodular | 0.436157996 | 2.015195746 | 0.021952325 |
| esophagus | 0.435455533 | 2.011978582 | 0.022109968 |
| sponge | 0.431732705 | 1.994928636 | 0.02302144 |
| hair | 0.429083797 | 1.98279707 | 0.023683715 |
| ampa | 0.428132397 | 1.978439812 | 0.023964353 |
| n-methyl-d-aspartic | 0.425785632 | 1.96769201 | 0.024534019 |
| synaptic | 0.424441748 | 1.961537242 | 0.024881242 |
| scalp | 0.423544139 | 1.95742634 | 0.025173734 |
| hard | 0.421757656 | 1.949244538 | 0.025647712 |
| epidermis | 0.420918155 | 1.945399761 | 0.025887489 |
| simple | 0.416982508 | 1.927375137 | 0.026989815 |
| corneum | 0.414741163 | 1.917110145 | 0.027618966 |
| synapses | 0.413207403 | 1.910085777 | 0.028066607 |
| stratum | 0.41301945 | 1.909224979 | 0.028131046 |
| epithelia | 0.41117309 | 1.900768954 | 0.028651006 |
| stressful | 0.40923192 | 1.891878709 | 0.02924549 |
| resilience | 0.397694844 | 1.839040785 | 0.032957593 |
| ficoll | 0.395652051 | 1.829685126 | 0.033624969 |
| papillae | 0.393788634 | 1.821150983 | 0.034303428 |
| keratinocytes | 0.390365612 | 1.805474098 | 0.035537341 |
| nmda | 0.389621172 | 1.802064683 | 0.035772703 |
| neostriatum | 0.38862324 | 1.797494317 | 0.03616781 |
| hypofunction | 0.388102549 | 1.795109637 | 0.03632685 |
| hippocampus | 0.38745229 | 1.792131558 | 0.036566483 |
| glutamatergic | 0.386496185 | 1.787752756 | 0.036888004 |
| miniature | 0.385446006 | 1.782943107 | 0.037293152 |
| barrett | 0.38536109 | 1.782554204 | 0.037293152 |
| memories | 0.383435149 | 1.77373371 | 0.038031575 |
| fuchs | 0.378973797 | 1.753301445 | 0.039801004 |
| agalactiae | 0.375573145 | 1.737727014 | 0.041105407 |
| excitatory | 0.3748307 | 1.734326736 | 0.041459042 |
| parakeratosis | 0.372605726 | 1.72413672 | 0.042353922 |
| nmdar | 0.369995545 | 1.712182516 | 0.043448334 |
| post-synaptic | 0.369974863 | 1.712087794 | 0.043448334 |
| episodic | 0.369174094 | 1.708420405 | 0.043818171 |
| ionotropic | 0.368690648 | 1.706206301 | 0.04400404 |
| n-methyl-d-aspartate | 0.368478118 | 1.705232947 | 0.044097212 |
| rearing | 0.368049528 | 1.703270077 | 0.044284034 |
| eccrine | 0.367651494 | 1.701447144 | 0.044471494 |
| nails | 0.366732209 | 1.697236969 | 0.04484833 |
| desmoplakin | 0.366490006 | 1.696127717 | 0.04494294 |
| sheath | 0.36286926 | 1.679545295 | 0.046478658 |
| anagen | 0.356192003 | 1.648964547 | 0.049573817 |
| somatosensory | 0.353597948 | 1.637084196 | 0.050815235 |
| skin | 0.353113288 | 1.634864534 | 0.051024524 |
| merkel | 0.349648518 | 1.61899645 | 0.052723632 |
| peristaltic | 0.347363017 | 1.608529229 | 0.053808171 |
| type-ii | 0.347332515 | 1.608389538 | 0.053917589 |
| iis | 0.346352408 | 1.603900804 | 0.054357026 |
| globus | 0.346150189 | 1.602974675 | 0.054467327 |
| gabaergic | 0.345466214 | 1.599842182 | 0.054799292 |
| fragility | 0.34524841 | 1.598844675 | 0.054910301 |
| avoidance | 0.344801156 | 1.596796323 | 0.055132854 |
| presynaptic | 0.341809842 | 1.583096596 | 0.056710732 |
| propionic | 0.341591473 | 1.582096501 | 0.056824785 |
| gustatory | 0.339693425 | 1.573403751 | 0.057859409 |
| spiny | 0.339388629 | 1.572007834 | 0.057975276 |
| smell | 0.339205893 | 1.571170933 | 0.058091324 |
| congenita | 0.339205637 | 1.571169759 | 0.058091324 |
| short-term | 0.339025213 | 1.570343448 | 0.058207556 |
| acquisition | 0.336559795 | 1.559052236 | 0.05949819 |
| kainic | 0.335952498 | 1.556270916 | 0.059854046 |
| areas | 0.334494369 | 1.549592923 | 0.060570758 |
| pads | 0.332355755 | 1.539798417 | 0.061780177 |
| vestibular | 0.331521829 | 1.535979175 | 0.06226919 |
| type-i | 0.32906313 | 1.524718731 | 0.063629549 |
| barrett's | 0.328868142 | 1.523825718 | 0.063754356 |
| appetitive | 0.328064696 | 1.52014607 | 0.064255488 |
| filament | 0.326788219 | 1.514300011 | 0.06501292 |
| ketamine | 0.325761901 | 1.509599643 | 0.065521712 |
| igd | 0.321714785 | 1.491064511 | 0.067980747 |
| superficial | 0.320936989 | 1.487502331 | 0.068375447 |
