# Supplementary material for: Hippocampal Transcriptomic and Proteomic Alterations in the BTBR Mouse Model of Autism Spectrum Disorder
Source: Front Physiol. 2015 Nov 24;6:324. doi: 10.3389/fphys.2015.00324 (PMC4656818; doi:10.3389/fphys.2015.00324)
Supplement: Supplementary file 11 [file Table10.DOCX]

**Table S10. Word counts for *Textrous!* noun-phrase extraction from BTBR hippocampal data.** For each extracted noun the number of occurences in the total output data is depicted. Word occurrence scores were calculated using WriteWords (WriteWords: http://www.writewords.org.uk/word_count.asp).

| **Word** | **Occurrences** |
| --- | --- |
| term | 234 |
| long | 171 |
| synaptic | 168 |
| hippocampal | 137 |
| memory | 132 |
| presynaptic | 121 |
| glutamate | 121 |
| plasticity | 89 |
| excitatory | 86 |
| synapses | 71 |
| potentiation | 69 |
| short | 66 |
| bipolar | 66 |
| vestibular | 65 |
| terminals | 54 |
| neurons | 54 |
| receptors | 53 |
| learning | 52 |
| dentate | 50 |
| gyrus | 47 |
| nmda | 43 |
| pyramidal | 42 |
| glutamatergic | 42 |
| transmission | 41 |
| cell | 41 |
| gabaergic | 40 |
| depression | 39 |
| cells | 38 |
| rb | 37 |
| pdz | 36 |
| fear | 33 |
| receptor | 31 |
| hippocampus | 31 |
| neurotrophic | 28 |
| mediated | 27 |
| ampa | 27 |
| mouse | 26 |
| dendrites | 26 |
| tubulin | 24 |
| retinoblastoma | 23 |
| postsynaptic | 23 |
| nerve | 23 |
| spatial | 22 |
| protein | 22 |
| enhanced | 21 |
| normal | 20 |
| maze | 20 |
| high | 20 |
| frequency | 20 |
| currents | 20 |
| human | 19 |
| episodic | 19 |
| brain | 19 |
| associative | 19 |
| adult | 19 |
| spines | 18 |
| rat | 18 |
| factor | 18 |
| training | 17 |
| release | 17 |
| nmdar | 17 |
| inhibitory | 17 |
| metabotropic | 16 |
| impaired | 16 |
| functional | 16 |
| domain | 16 |
| derived | 16 |
| cortical | 16 |
| water | 15 |
| somatosensory | 15 |
| neuronal | 15 |
| miniature | 15 |
| disorder | 15 |
| type | 14 |
| primary | 14 |
| innervation | 14 |
| dendritic | 14 |
| transmitter | 13 |
| system | 13 |
| memories | 13 |
| kainate | 13 |
| increased | 13 |
| granule | 13 |
| fiber | 13 |
| alpha | 13 |
| abnormal | 13 |
| terminal | 12 |
| occipital | 12 |
| responses | 11 |
| pathway | 11 |
| neuron | 11 |
| n | 11 |
| mossy | 11 |
| morris | 11 |
| library | 11 |
| layer | 11 |
| cortex | 11 |
| cdna | 11 |
| beta | 11 |
| anchorage | 11 |
| task | 10 |
| performance | 10 |
| papillae | 10 |
| extinction | 10 |
| development | 10 |
| depressed | 10 |
| cingulate | 10 |
| calcium | 10 |
| activity | 10 |
| acid | 10 |
| rod | 9 |
| potentials | 9 |
| neurotransmitter | 9 |
| neurotransmission | 9 |
| membrane | 9 |
| defective | 9 |
| complex | 9 |
| ciliary | 9 |
| central | 9 |
| axonemes | 9 |
| working | 8 |
| tumor | 8 |
| stimulation | 8 |
| spikes | 8 |
| specific | 8 |
| response | 8 |
| proteins | 8 |
| methyl | 8 |
| mature | 8 |
| kainic | 8 |
| interneurons | 8 |
| induction | 8 |
| hypofunction | 8 |
| formation | 8 |
| endogenous | 8 |
| deficits | 8 |
| defects | 8 |
| d | 8 |
| current | 8 |
| cerebellar | 8 |
| basal | 8 |
| axoneme | 8 |
| spindle | 7 |
| retention | 7 |
| plasma | 7 |
| p | 7 |
| motor | 7 |
| major | 7 |
| late | 7 |
| ionotropic | 7 |
| ii | 7 |
| c | 7 |
| aspartate | 7 |
| treatment | 6 |
| transporter | 6 |
| transport | 6 |
| transmissions | 6 |
| toxicity | 6 |
| suppressor | 6 |
| spontaneous | 6 |
| sites | 6 |
| single | 6 |
| sensory | 6 |
| processes | 6 |
| nucleus | 6 |
| multiple | 6 |
| loss | 6 |
| levels | 6 |
| hair | 6 |
| facilitation | 6 |
| extracellular | 6 |
| expression | 6 |
| evoked | 6 |
| dysfunction | 6 |
| developing | 6 |
| defect | 6 |
| conserved | 6 |
| conditioned | 6 |
| channels | 6 |
| cellular | 6 |
| affective | 6 |
| administration | 6 |
| vesicular | 5 |
| vesicle | 5 |
| verbal | 5 |
| tests | 5 |
| superior | 5 |
| strength | 5 |
| spine | 5 |
| retinal | 5 |
| region | 5 |
| photoreceptor | 5 |
| patients | 5 |
| organs | 5 |
| neurotrophin | 5 |
| mutant | 5 |
| motif | 5 |
| mechanism | 5 |
| maturation | 5 |
| markers | 5 |
| ligand | 5 |
| hcs | 5 |
| fast | 5 |
| endplate | 5 |
| eating | 5 |
| early | 5 |
| distal | 5 |
| differentiation | 5 |
| dependent | 5 |
| deficit | 5 |
| cultured | 5 |
| binding | 5 |
| axon | 5 |
| anterior | 5 |
| activation | 5 |
| uptake | 4 |
| unipolar | 4 |
| transporters | 4 |
| temporal | 4 |
| synthesis | 4 |
| syndrome | 4 |
| synapse | 4 |
| symptoms | 4 |
| sympathetic | 4 |
| site | 4 |
| signs | 4 |
| signaling | 4 |
| rodent | 4 |
| radial | 4 |
| promoter | 4 |
| production | 4 |
| phosphorylation | 4 |
| optokinetic | 4 |
| nuclei | 4 |
| neural | 4 |
| mitotic | 4 |
| lines | 4 |
| layers | 4 |
| isolated | 4 |
| higher | 4 |
| geniculate | 4 |
| forebrain | 4 |
| endings | 4 |
| ectopic | 4 |
| dopamine | 4 |
| disabilities | 4 |
| dehydrogenase | 4 |
| deficiency | 4 |
| containing | 4 |
| connections | 4 |
| conditioning | 4 |
| concentrations | 4 |
| blocks | 4 |
| auditory | 4 |
| areas | 4 |
| affinity | 4 |
| active | 4 |
| volume | 3 |
| visual | 3 |
| variable | 3 |
| transaminase | 3 |
| trace | 3 |
| tissue | 3 |
| survival | 3 |
| structural | 3 |
| storage | 3 |
| small | 3 |
| schwannomas | 3 |
| role | 3 |
| respiratory | 3 |
| residue | 3 |
| remitting | 3 |
| related | 3 |
| relapsing | 3 |
| reference | 3 |
| reduced | 3 |
| reconstitution | 3 |
| recall | 3 |
| purkinje | 3 |
| properties | 3 |
| proliferation | 3 |
| potential | 3 |
| piriform | 3 |
| phenotypes | 3 |
| phenotype | 3 |
| partial | 3 |
| oxaloacetate | 3 |
| onset | 3 |
| nuclear | 3 |
| mutants | 3 |
| mrna | 3 |
| morphogenesis | 3 |
| mild | 3 |
| mental | 3 |
| membranes | 3 |
| machinery | 3 |
| locus | 3 |
| line | 3 |
| individual | 3 |
| inactivation | 3 |
| hippocampi | 3 |
| hearing | 3 |
| gustatory | 3 |
| glial | 3 |
| genes | 3 |
| ganglion | 3 |
| functions | 3 |
| form | 3 |
| folding | 3 |
| field | 3 |
| excitotoxin | 3 |
| events | 3 |
| endurance | 3 |
| emotional | 3 |
| electroconvulsive | 3 |
| effects | 3 |
| dopaminergic | 3 |
| domains | 3 |
| disorders | 3 |
| disease | 3 |
| dermal | 3 |
| delivery | 3 |
| decreased | 3 |
| decline | 3 |
| cytosolic | 3 |
| cytoplasmic | 3 |
| cultures | 3 |
| culture | 3 |
| control | 3 |
| consolidation | 3 |
| cone | 3 |
| cholinergic | 3 |
| chicken | 3 |
| chemical | 3 |
| capacity | 3 |
| blockade | 3 |
| bilateral | 3 |
| bidirectional | 3 |
| ataxia | 3 |
| area | 3 |
| apparatus | 3 |
| agonist | 3 |
| afferent | 3 |
| actions | 3 |
| weakness | 2 |
| voltage | 2 |
| vocal | 2 |
| vivo | 2 |
| victims | 2 |
| vesicles | 2 |
| utricle | 2 |
| unilateral | 2 |
| trials | 2 |
| tract | 2 |
| total | 2 |
| target | 2 |
| support | 2 |
| subtype | 2 |
| structures | 2 |
| stretch | 2 |
| stable | 2 |
| squid | 2 |
| spreading | 2 |
| spindles | 2 |
| signals | 2 |
| signal | 2 |
| severe | 2 |
| sequence | 2 |
| secretion | 2 |
| sclerosis | 2 |
| reversible | 2 |
| residues | 2 |
| remote | 2 |
| regulation | 2 |
| regulating | 2 |
| regions | 2 |
| reflex | 2 |
| reduction | 2 |
| recurrent | 2 |
| recognition | 2 |
| rapid | 2 |
| proximal | 2 |
| proper | 2 |
| prolonged | 2 |
| proliferative | 2 |
| projection | 2 |
| progenitor | 2 |
| process | 2 |
| problems | 2 |
| probability | 2 |
| principal | 2 |
| postnatal | 2 |
| postmortem | 2 |
| posterior | 2 |
| phosphatase | 2 |
| persistent | 2 |
| persistence | 2 |
| period | 2 |
| parallel | 2 |
| organization | 2 |
| organ | 2 |
| oral | 2 |
| number | 2 |
| nicotinic | 2 |
| neurotrophins | 2 |
| neurogenesis | 2 |
| monocular | 2 |
| molecules | 2 |
| molecule | 2 |
| modulating | 2 |
| microtubule | 2 |
| metallochaperone | 2 |
| marker | 2 |
| malfunction | 2 |
| lower | 2 |
| low | 2 |
| localization | 2 |
| like | 2 |
| libraries | 2 |
| lateral | 2 |
| lasting | 2 |
| involvement | 2 |
| interactions | 2 |
| interaction | 2 |
| integrity | 2 |
| injection | 2 |
| inhibition | 2 |
| induced | 2 |
| independent | 2 |
| increase | 2 |
| impairment | 2 |
| immune | 2 |
| immature | 2 |
| illness | 2 |
| humoral | 2 |
| homeostasis | 2 |
| highly | 2 |
| health | 2 |
| growth | 2 |
| granular | 2 |
| glycogen | 2 |
| giant | 2 |
| genome | 2 |
| gated | 2 |
| ganglia | 2 |
| functionally | 2 |
| fimbria | 2 |
| fields | 2 |
| familial | 2 |
| exercise | 2 |
| essential | 2 |
| epithelium | 2 |
| epithelial | 2 |
| epithelia | 2 |
| epileptic | 2 |
| enucleation | 2 |
| end | 2 |
| elevated | 2 |
| effector | 2 |
| edwards | 2 |
| depletion | 2 |
| dependence | 2 |
| density | 2 |
| death | 2 |
| damage | 2 |
| cytokine | 2 |
| components | 2 |
| complete | 2 |
| competitive | 2 |
| compartments | 2 |
| class | 2 |
| characteristics | 2 |
| channel | 2 |
| cerebral | 2 |
| cardiac | 2 |
| bridge | 2 |
| bodies | 2 |
| blocking | 2 |
| behavior | 2 |
| b | 2 |
| autoreceptor | 2 |
| atrophy | 2 |
| assembly | 2 |
| aqueduct | 2 |
| antagonist | 2 |
| age | 2 |
| adaptive | 2 |
| acquisition | 2 |
| abnormalities | 2 |
| ability | 2 |
| zones | 1 |
| zone | 1 |
| zinc | 1 |
| x | 1 |
| wiring | 1 |
| weight | 1 |
| vibrations | 1 |
| viability | 1 |
| vertebrate | 1 |
| ventral | 1 |
| velocity | 1 |
| variants | 1 |
| upstream | 1 |
| up | 1 |
| unwanted | 1 |
| universal | 1 |
| ultrastructure | 1 |
| ultrasonic | 1 |
| ubiquitous | 1 |
| tyrosine | 1 |
| typical | 1 |
| transfection | 1 |
| transcripts | 1 |
| transcript | 1 |
| trafficking | 1 |
| tracts | 1 |
| tooth | 1 |
| tonic | 1 |
| tongue | 1 |
| tone | 1 |
| tissues | 1 |
| tension | 1 |
| temperature | 1 |
| tegmental | 1 |
| tagging | 1 |
| tagged | 1 |
| t | 1 |
| systolic | 1 |
| systemic | 1 |
| synchronous | 1 |
| synaptophysin | 1 |
| sustained | 1 |
| susceptibility | 1 |
| survivors | 1 |
| suppression | 1 |
| supply | 1 |
| superfamily | 1 |
| suicide | 1 |
| subunits | 1 |
| subunit | 1 |
| subtypes | 1 |
| substrate | 1 |
| subjects | 1 |
| subjective | 1 |
| studies | 1 |
| stressful | 1 |
| strengthening | 1 |
| stimulated | 1 |
| stereocilia | 1 |
| status | 1 |
| starvation | 1 |
| staining | 1 |
| stabilization | 1 |
| sporadic | 1 |
| spinal | 1 |
| spiking | 1 |
| sperm | 1 |
| specificity | 1 |
| spaces | 1 |
| space | 1 |
| somatic | 1 |
| sodium | 1 |
| smaller | 1 |
| side | 1 |
| shock | 1 |
| shapes | 1 |
| shape | 1 |
| series | 1 |
| sequences | 1 |
| sensorineural | 1 |
| selective | 1 |
| selection | 1 |
| seizures | 1 |
| seizure | 1 |
| screening | 1 |
| schwannoma | 1 |
| scaling | 1 |
| saccule | 1 |
| rules | 1 |
| roles | 1 |
| rigid | 1 |
| reversal | 1 |
| retrograde | 1 |
| retrieval | 1 |
| retraction | 1 |
| results | 1 |
| restriction | 1 |
| resolution | 1 |
| residual | 1 |
| required | 1 |
| repression | 1 |
| representation | 1 |
| repetitive | 1 |
| removal | 1 |
| reinforcement | 1 |
| regular | 1 |
| reflexes | 1 |
| recycling | 1 |
| recovery | 1 |
| reciprocal | 1 |
| ras | 1 |
| range | 1 |
| radiolabeled | 1 |
| purinergic | 1 |
| pumps | 1 |
| psychotic | 1 |
| pseudogenes | 1 |
| protection | 1 |
| proprotein | 1 |
| propionic | 1 |
| progressive | 1 |
| programming | 1 |
| prognosis | 1 |
| processing | 1 |
| probe | 1 |
| presenilin | 1 |
| predominant | 1 |
| precursors | 1 |
| precursor | 1 |
| potassium | 1 |
| post | 1 |
| positive | 1 |
| position | 1 |
| pools | 1 |
| polypeptides | 1 |
| polymer | 1 |
| pocket | 1 |
| pka | 1 |
| physiology | 1 |
| physical | 1 |
| phosphorylating | 1 |
| phosphoprotein | 1 |
| phospho | 1 |
| phenomena | 1 |
| permanent | 1 |
| perivascular | 1 |
| peristaltic | 1 |
| peripheral | 1 |
| pentylenetetrazol | 1 |
| pedigree | 1 |
| pattern | 1 |
| pathways | 1 |
| paternal | 1 |
| partners | 1 |
| partially | 1 |
| part | 1 |
| parasympathetic | 1 |
| paralysis | 1 |
| oxidation | 1 |
| ovarian | 1 |
| oscillations | 1 |
| organizing | 1 |
| organizer | 1 |
| open | 1 |
| ocular | 1 |
| occurring | 1 |
| nociceptor | 1 |
| niche | 1 |
| neurotoxin | 1 |
| neuroplasticity | 1 |
| neuromuscular | 1 |
| neurogenic | 1 |
| neurite | 1 |
| nervous | 1 |
| neostriatum | 1 |
| negative | 1 |
| naturally | 1 |
| native | 1 |
| nasal | 1 |
| mushroom | 1 |
| muscle | 1 |
| murine | 1 |
| multiprotein | 1 |
| mtdna | 1 |
| movements | 1 |
| motifs | 1 |
| mood | 1 |
| monosodium | 1 |
| monocyte | 1 |
| molar | 1 |
| modulated | 1 |
| modifications | 1 |
| mitochondrial | 1 |
| microvascular | 1 |
| mesenchymal | 1 |
| median | 1 |
| medial | 1 |
| mechanotransduction | 1 |
| mechanoreceptor | 1 |
| mechanisms | 1 |
| maximum | 1 |
| matrix | 1 |
| maternal | 1 |
| manic | 1 |
| mammalian | 1 |
| male | 1 |
| maintenance | 1 |
| macular | 1 |
| macromolecular | 1 |
| local | 1 |
| lobes | 1 |
| lobe | 1 |
| loading | 1 |
| linked | 1 |
| lingual | 1 |
| lineage | 1 |
| leukemia | 1 |
| lesions | 1 |
| leptin | 1 |
| lectins | 1 |
| learned | 1 |
| latent | 1 |
| language | 1 |
| labyrinth | 1 |
| labeling | 1 |
| knockdown | 1 |
| ion | 1 |
| invariant | 1 |
| intrinsic | 1 |
| intraperitoneal | 1 |
| intracerebral | 1 |
| internalization | 1 |
| interacting | 1 |
| intensive | 1 |
| insufficient | 1 |
| inhibitor | 1 |
| infusion | 1 |
| inflammation | 1 |
| independence | 1 |
| incubation | 1 |
| increases | 1 |
| inappropriate | 1 |
| impulse | 1 |
| impairments | 1 |
| immunologic | 1 |
| iii | 1 |
| hypoplastic | 1 |
| huntington's | 1 |
| horns | 1 |
| horn | 1 |
| horizontal | 1 |
| homogeneous | 1 |
| history | 1 |
| histories | 1 |
| hereditary | 1 |
| hemodialysis | 1 |
| healthy | 1 |
| haplotype | 1 |
| groups | 1 |
| group | 1 |
| groove | 1 |
| greater | 1 |
| gradient | 1 |
| golgi | 1 |
| glycoprotein | 1 |
| glycinergic | 1 |
| glycine | 1 |
| genomic | 1 |
| generalization | 1 |
| gate | 1 |
| gaba | 1 |
| frontal | 1 |
| fractions | 1 |
| forms | 1 |
| food | 1 |
| follow | 1 |
| focus | 1 |
| flux | 1 |
| flagella | 1 |
| fire | 1 |
| fibers | 1 |
| female | 1 |
| feedback | 1 |
| families | 1 |
| failure | 1 |
| factors | 1 |
| facilitated | 1 |
| extraordinary | 1 |
| extension | 1 |
| exposure | 1 |
| experiments | 1 |
| experiences | 1 |
| expansions | 1 |
| exercises | 1 |
| excitation | 1 |
| excess | 1 |
| episodes | 1 |
| epilepsies | 1 |
| epigenetic | 1 |
| epididymal | 1 |
| ephrins | 1 |
| enzyme | 1 |
| enlarged | 1 |
| endothelial | 1 |
| endosomes | 1 |
| endolymph | 1 |
| encephalopathy | 1 |
| eminence | 1 |
| embryonic | 1 |
| elimination | 1 |
| elements | 1 |
| electrical | 1 |
| efflux | 1 |
| efficiency | 1 |
| efficacy | 1 |
| dystonic | 1 |
| dysgenesis | 1 |
| drug | 1 |
| drives | 1 |
| drive | 1 |
| dominance | 1 |
| disturbed | 1 |
| distribution | 1 |
| dissociated | 1 |
| disorganized | 1 |
| disability | 1 |
| difficulty | 1 |
| differentiated | 1 |
| dialysis | 1 |
| desmosomal | 1 |
| desensitization | 1 |
| depressing | 1 |
| dental | 1 |
| dendrite | 1 |
| deletion | 1 |
| degradation | 1 |
| degeneration | 1 |
| defined | 1 |
| deep | 1 |
| decreases | 1 |
| decarboxylase | 1 |
| deafferentation | 1 |
| da | 1 |
| cytotoxicity | 1 |
| cytosol | 1 |
| cytoskeletal | 1 |
| cycle | 1 |
| critical | 1 |
| corticostriatal | 1 |
| cortices | 1 |
| contribution | 1 |
| contractility | 1 |
| contractile | 1 |
| continuous | 1 |
| contacts | 1 |
| constant | 1 |
| connecting | 1 |
| confusion | 1 |
| cones | 1 |
| conditions | 1 |
| condition | 1 |
| concentration | 1 |
| component | 1 |
| complications | 1 |
| compartment | 1 |
| common | 1 |
| commissure | 1 |
| cognate | 1 |
| cns | 1 |
| clusters | 1 |
| clustering | 1 |
| clinic | 1 |
| clefts | 1 |
| cleft | 1 |
| clearance | 1 |
| circuits | 1 |
| cilium | 1 |
| cilia | 1 |
| chromatin | 1 |
| childhood | 1 |
| chief | 1 |
| chains | 1 |
| cervical | 1 |
| centers | 1 |
| catalytic | 1 |
| cases | 1 |
| carriers | 1 |
| carboxy | 1 |
| capture | 1 |
| cannabinoid | 1 |
| calorie | 1 |
| calls | 1 |
| calcifications | 1 |
| calcification | 1 |
| burst | 1 |
| bundles | 1 |
| building | 1 |
| bridging | 1 |
| brainstem | 1 |
| bovine | 1 |
| boutons | 1 |
| block | 1 |
| benefits | 1 |
| behaviors | 1 |
| behavio | 1 |
| beginning | 1 |
| bath | 1 |
| basis | 1 |
| axons | 1 |
| axonal | 1 |
| avoidance | 1 |
| aversive | 1 |
| autoreceptors | 1 |
| autoimmune | 1 |
| attachment | 1 |
| atpases | 1 |
| asymptomatic | 1 |
| asymmetric | 1 |
| astrocytic | 1 |
| associations | 1 |
| associates | 1 |
| aspartic | 1 |
| array | 1 |
| arrangement | 1 |
| arbor | 1 |
| apical | 1 |
| antibody | 1 |
| antagonists | 1 |
| antagonism | 1 |
| analysis | 1 |
| amacrine | 1 |
| allele | 1 |
| alignment | 1 |
| airway | 1 |
| agents | 1 |
| afferents | 1 |
| adaptors | 1 |
| adaptation | 1 |
| acute | 1 |
| activations | 1 |
| activated | 1 |
| action | 1 |
| actin | 1 |
| acquired | 1 |
| acetylation | 1 |
| acetylated | 1 |
