# Supplementary material for: Hippocampal Transcriptomic and Proteomic Alterations in the BTBR Mouse Model of Autism Spectrum Disorder
Source: Front Physiol. 2015 Nov 24;6:324. doi: 10.3389/fphys.2015.00324 (PMC4656818; doi:10.3389/fphys.2015.00324)
Supplement: Supplementary file 12 [file Table11.DOCX]

**Table S11. Quantitative proteomic analysis of BTBR hippocampus compared to B6.** For each protein reliably and differentially regulated between the BTBR and B6 hippocampal tissues, the Gene Symbol, Protein Description and Log_2_-transformed BTBR:B6 iTRAQ ratio is given.

| **Gene Symbol** | **Protein Description** | **Log_2_ ratio BTBR:B6** |
| --- | --- | --- |
| Stxbp1 | syntaxin binding protein 1 [Mus musculus] | 4.593007712 |
| Oxr1 | Oxr1 protein [Mus musculus] | 2.300396576 |
| Tom1L2 | target of myb1-like protein 2 [Mus musculus] | 1.326426443 |
| A1m | Alpha-1-macroglobulin precursor (Alpha-1-M) [Contains: Alpha-1-macroglobulin 165 kDa subunit; Alpha-1-macroglobulin 45 kDa subunit] | 1.231432348 |
| Agk | acylglycerol kinase [Mus musculus] | 0.869637229 |
| Chmp6 | chromatin modifying protein 6 [Mus musculus] | 0.867120543 |
| Mrpl53 | mitochondrial ribosomal protein L53 [Mus musculus] | 0.78084818 |
| Nup133 | Nuclear pore complex protein Nup133 (Nucleoporin Nup133) (133 kDa nucleoporin) | 0.776914015 |
| Pnmal2 | PNMA-like 2 [Mus musculus] | 0.707320944 |
| Arl6ip1 | ADP-ribosylation factor-like 6 interacting protein 1 [Mus musculus] | 0.644919469 |
| Pitpnm3 | Pitpnm family member 3 [Mus musculus] | 0.633503524 |
| Wfs1 | Wolfram syndrome 1 homolog (human) [Mus musculus] | 0.632919557 |
| Mut | Mut protein [Mus musculus] | 0.622432948 |
| Gipc3 | GIPC PDZ domain containing family, member 3 [Mus musculus] | 0.595765802 |
| Apoa1 | apolipoprotein A-I [Mus musculus] | 0.580040861 |
| Phpt1 | phosphohistidine phosphatase [Mus musculus] | 0.572419664 |
| Lrsam1 | leucine rich repeat and sterile alpha motif containing 1 [Mus musculus] | 0.489345753 |
| Ogfod1 | Ogfod1 protein [Mus musculus] | 0.475610385 |
| Lancl1 | LanC (bacterial lantibiotic synthetase component C)-like 1 [Mus musculus] | 0.44641324 |
| Rell2 | RELT-like 2 [Mus musculus] | 0.440953863 |
| Ccdc6 | coiled-coil domain containing 6 [Mus musculus] | 0.426566787 |
| Fam120c | family with sequence similarity 120C ; constitutive coactivator of PPAR-gamma-like protein 2 [Mus musculus] | 0.425385007 |
| Sf1 | splicing factor 1 [Mus musculus] | 0.423156042 |
| Gnb2 | guanine nucleotide-binding protein, beta-2 subunit [Mus musculus] | 0.423124526 |
| Dync1i1 | Cytoplasmic dynein 1 intermediate chain 1 (Cytoplasmic dynein intermediate chain 1) (Dynein intermediate chain 1, cytosolic) (DH IC-1) | 0.420080783 |
| Prnp | Chain A, Mouse Prion Protein Fragment 121-231 | 0.418152629 |
| Macf1 | Microtubule-actin cross-linking factor 1 (Actin cross-linking family 7) | 0.417387351 |
| Cpeb3 | cytoplasmic polyadenylation element binding protein 3 [Mus musculus] | 0.411636951 |
| Pnck | Protein Kinase [Mus musculus] | 0.40605819 |
| Iap | integrin-associated protein form 4 [Mus musculus] | 0.405325877 |
| Ppih | peptidylprolyl isomerase H [Homo sapiens] | 0.404091805 |
| Atp5d | ATP synthase, H+ transporting, mitochondrial F1 complex, delta subunit precursor [Mus musculus] | 0.401263731 |
| Nelf | nasal embryonic LHRH factor [Mus musculus] | 0.394143483 |
| Atp5c1 | ATP synthase, H+ transporting, mitochondrial F1 complex, gamma subunit isoform b [Mus musculus] | 0.392194675 |
| Ncam1 | Neural cell adhesion molecule 1, 180 kDa isoform precursor (N-CAM 180) (NCAM-180) (CD56 antigen) | 0.391891452 |
| Gm996 | Gene model 996, (NCBI) [Mus musculus] | 0.381242477 |
| Cox4i1 | cytochrome c oxidase subunit IV isoform 1 [Mus musculus] | 0.37725963 |
| Uqcrc1 | Cytochrome b-c1 complex subunit 1, mitochondrial precursor (Ubiquinol-cytochrome-c reductase complex core protein 1) (Core protein I) (Complex III subunit 1) | 0.376724191 |
| Elp3 | Elp3 protein [Mus musculus] | 0.37284425 |
| Ndpk2 | nucleoside-diphosphate kinase 2 [Mus musculus] | 0.372591955 |
| Gap43 | growth associated protein 43 [Mus musculus] | 0.366550831 |
| Wnk1 | WNK lysine deficient protein kinase 1 [Mus musculus] | 0.36350444 |
| Eif3f | Eukaryotic translation initiation factor 3, subunit F [Mus musculus] | 0.359186921 |
| Enah | NDPP-1 protein [Mus musculus] | 0.358948923 |
| Brsk2 | brain-selective kinase 2 isoform alpha [Mus musculus] | 0.352881789 |
| Chchd3 | coiled-coil-helix-coiled-coil-helix domain containing 3 [Mus musculus] | 0.351042206 |
| Fkbp12 | FK506-binding protein [Mus musculus] | 0.346204908 |
| Cend1 | cell cycle exit and neuronal differentiation 1 [Mus musculus] | 0.346175241 |
| Atp5e | H(+)-ATP synthase epsilon-subunit [rats, liver, Peptide Mitochondrial, 50 aa] | 0.342496094 |
| Prdx3 | peroxiredoxin 3 [Mus musculus] | 0.338559816 |
| Shroom2 | shroom family member 2 [Mus musculus] | 0.33767229 |
| Gnb1 | guanine nucleotide-binding protein, beta-1 subunit [Mus musculus] | 0.336362479 |
| Cfl2 | cofilin 2, muscle [Mus musculus] | 0.335809196 |
| Sapap3 | synapse-associated protein 90/postsynaptic density-95-associated protein 3(-) [Mus musculus] | 0.334626451 |
| Scn1b | sodium channel, voltage-gated, type I, beta [Mus musculus] | 0.332890894 |
| Ank2 | ankyrin 2, brain [Mus musculus] | 0.331664644 |
| Rnf214 | ring finger protein 214 [Mus musculus] | 0.330012094 |
| Lppr4 | lipid phosphate phosphatase-related protein type 4 [Mus musculus] | 0.326871424 |
| Arhgef2 | rho/rac guanine nucleotide exchange factor (GEF) 2 [Mus musculus] | 0.324896277 |
| Trpc4 | transient receptor potential cation channel, subfamily C, member 4 [Mus musculus] | 0.323180455 |
| Cast | CAST1/ERC2 splicing variant-1 [Mus musculus] | 0.322823862 |
| Cacng8 | voltage-dependent calcium channel gamma-8 subunit [Mus musculus] | 0.321788911 |
| Ccdc32 | Coiled-coil domain containing 32 [Mus musculus] | 0.312256225 |
| GcvT | aminomethyltransferase gcvT [Mus musculus] | 0.312138633 |
| Rph3a | rabphilin 3A [Mus musculus] | 0.311388376 |
| Hnrnpu | SP120 [Mus musculus] | 0.307939386 |
| Trappc8 | trafficking protein particle complex 8 [Mus musculus] | 0.306421207 |
| Bsn | Protein bassoon [Mus musculus] | 0.304497591 |
| Dek | DEK oncogene (DNA binding) [Mus musculus] | 0.304117736 |
| Tsc1 | Tsc1 protein [Mus musculus] | 0.298816493 |
| Slc25a3 | solute carrier family 25 (mitochondrial carrier; phosphate carrier), member 3 [Mus musculus] | 0.297254698 |
| Mkk3 | mitogen-activated protein kinase kinase 3 [Mus musculus] | 0.294692963 |
| PnbA | Chain A, Crystal Structure Of The Mouse Acetylcholinesterase- Gallamine Complex | 0.294468797 |
| Dlg1 | discs, large homolog 1 (Drosophila) [Mus musculus] | 0.293678899 |
| Pfetin | BTB/POZ domain-containing protein KCTD12 (Pfetin) (Predominantly fetal expressed T1 domain) | 0.292735718 |
| Eps15l1 | epidermal growth factor receptor pathway substrate 15-like 1 [Mus musculus] | 0.291126304 |
| Gpx4 | phospholipid hydroperoxide glutathione peroxidase [Mus musculus] | 0.290635954 |
| Ma6d1 | MAP6 domain-containing protein 1 [Mus musculus] | 0.290350869 |
| Rps28 | ribosomal protein S28 [Mus musculus] | 0.287237226 |
| Atp5j2 | ATP synthase, H+ transporting, mitochondrial F0 complex, subunit f, isoform 2 [Mus musculus] | 0.287155494 |
| Clcn6 | chloride channel 6 [Mus musculus] | 0.286519752 |
| Zfp238 | zinc finger protein 238 isoform 2 [Mus musculus] | 0.285069055 |
| Fkbp8 | Bcl-2 inhibitor of transcription isoform b [Mus musculus] | 0.284989459 |
| Hnrph1 | heterogeneous nuclear ribonucleoprotein H1 [Mus musculus] | 0.284695375 |
| Kqt2 | potassium voltage-gated channel, subfamily Q, member 2 [Mus musculus] | 0.28372831 |
| Opcml | opioid binding protein/cell adhesion molecule-like [Mus musculus] | 0.281924374 |
| Git1 | G protein-coupled receptor kinase-interactor 1 [Mus musculus] | 0.281361758 |
| Spna2 | spectrin alpha 2 [Mus musculus] | 0.278550826 |
| Glrx | glutaredoxin [Mus musculus] | 0.278158567 |
| Snrpa1 | U2 small nuclear ribonucleoprotein A' [Mus musculus] | 0.275979234 |
| Arpc1b | actin related protein 2/3 complex, subunit 1B [Mus musculus] | 0.274084729 |
| Serinc5 | serine incorporator 5 [Mus musculus] | 0.273493978 |
| Pcdh1 | protocadherin 1 [Mus musculus] | 0.272774747 |
| Ndufb11 | neuronal protein 15.6 [Mus musculus] | 0.272218888 |
| Sept9 | septin 9 isoform a [Mus musculus] | 0.271503421 |
| Psd3 | pleckstrin and Sec7 domain containing 3 [Mus musculus] | 0.269662143 |
| Atp2b2 | ATPase, Ca++ transporting, plasma membrane 2 [Mus musculus] | 0.269373264 |
| Eif4ebp2 | eukaryotic translation initiation factor 4E binding protein 2 [Mus musculus] | 0.268678934 |
| Cenpv | proline-rich polypeptide 6 isoform 1 [Mus musculus] | 0.268015885 |
| Slc25a22 | solute carrier family 25 (mitochondrial carrier, glutamate), member 22 [Mus musculus] | 0.26690192 |
| Scpdh | Probable saccharopine dehydrogenase [Mus musculus] | 0.264487689 |
| Pak2 | Serine/threonine-protein kinase PAK 2 (p21-activated kinase 2) (PAK-2) (Gamma-PAK) [Contains: PAK-2p27; PAK-2p34] | -0.33160434 |
| Mosc2 | MOCO sulphurase C-terminal domain containing 2 [Mus musculus] | -0.338953804 |
| Serpinb1a | serine (or cysteine) peptidase inhibitor, clade B, member 1a [Mus musculus] | -0.347479488 |
| Ppip5k2 | diphosphoinositol pentakisphosphate kinase 2 [Mus musculus] | -0.353112738 |
| Arl1 | Arl1 protein [Mus musculus] | -0.366852004 |
| Akap12 | A kinase (PRKA) anchor protein (gravin) 12 [Mus musculus] | -0.416223657 |
| Epha3 | Eph receptor A3 [Mus musculus] | -0.470707895 |
| Rpn1 | ribophorin [Mus musculus] | -0.561576371 |
| Dgkh | diacylglycerol kinase, eta isoform 2 [Mus musculus] | -0.661733151 |
| A030007L17Rik | hypothetical protein LOC68252 [Mus musculus] | -0.663617713 |
| Hba-a1 | hemoglobin alpha, adult chain 1 [Mus musculus] | -0.946924654 |
| Rock2 | Rho-associated coiled-coil containing protein kinase 2 [Mus musculus] | -2.797789665 |
