# Supplementary material for: Hippocampal Transcriptomic and Proteomic Alterations in the BTBR Mouse Model of Autism Spectrum Disorder
Source: Front Physiol. 2015 Nov 24;6:324. doi: 10.3389/fphys.2015.00324 (PMC4656818; doi:10.3389/fphys.2015.00324)
Supplement: Supplementary file 13 [file Table12.DOCX]

**Table S12. KEGG pathway analysis for proteins differentially regulated in BTBR hippocampus compared to B6 controls.** Significantly-populated KEGG signaling pathways, generated using the proteins differentially regulated in BTBR hippocampus compared to B6 controls, are depicted. For each specific KEGG pathway annotation the following parameter indices are indicated: **C** - total background number of transcripts populating the KEGG pathway; **O** – number of observed transcripts within the input dataset that are contained within the specific KEGG pathway; **E** – number of transcripts from the input dataset expected to be present at background levels; **R** – transcript enrichment factor in specific KEGG pathway, **P** – enrichment probability; **H** – hybrid score = -log_10_P * R.

| **KEGG Pathway** | **C** | **O** | **E** | **R** | **P** | **H** |
| --- | --- | --- | --- | --- | --- | --- |
| Oxidative phosphorylation | 147 | 7 | 0.21 | 34.02 | 3.17E-08 | 255.114 |
| Parkinson's disease | 148 | 6 | 0.21 | 28.96 | 6.31E-07 | 179.5511 |
| Huntington's disease | 197 | 6 | 0.28 | 21.76 | 1.71E-06 | 125.49 |
| Alzheimer's disease | 188 | 6 | 0.26 | 22.8 | 1.71E-06 | 131.4877 |
| Regulation of actin cytoskeleton | 216 | 6 | 0.3 | 19.84 | 2.34E-06 | 111.7148 |
| Axon guidance | 131 | 4 | 0.18 | 21.81 | 0.0001 | 87.24 |
| Cardiac muscle contraction | 81 | 3 | 0.11 | 26.46 | 0.0005 | 87.34525 |
| Metabolic pathways | 1184 | 8 | 1.66 | 4.83 | 0.0007 | 15.23818 |
| Prion diseases | 35 | 2 | 0.05 | 40.82 | 0.002 | 110.172 |
| Spliceosome | 138 | 3 | 0.19 | 15.53 | 0.002 | 41.915 |
| Chemokine signaling pathway | 185 | 3 | 0.26 | 11.58 | 0.0038 | 28.02611 |
| Glutathione metabolism | 54 | 2 | 0.08 | 26.46 | 0.0039 | 63.74043 |
| Fc gamma R-mediated phagocytosis | 90 | 2 | 0.13 | 15.87 | 0.01 | 31.74 |
| RNA transport | 168 | 2 | 0.24 | 8.5 | 0.0283 | 13.15982 |
| Protein processing in endoplasmic reticulum | 169 | 2 | 0.24 | 8.45 | 0.0283 | 13.0824 |
| Focal adhesion | 200 | 2 | 0.28 | 7.14 | 0.0362 | 10.29082 |
| Endocytosis | 220 | 2 | 0.31 | 6.49 | 0.0406 | 9.030666 |
