# Supplementary material for: Hippocampal Transcriptomic and Proteomic Alterations in the BTBR Mouse Model of Autism Spectrum Disorder
Source: Front Physiol. 2015 Nov 24;6:324. doi: 10.3389/fphys.2015.00324 (PMC4656818; doi:10.3389/fphys.2015.00324)
Supplement: Supplementary file 14 [file Table13.DOCX]

**Table S13. Canonical Pathway Signaling analysis for proteins differentially regulated in BTBR hippocampus compared to B6 controls.** Significantly-populated canonical signaling pathways, generated using proteins differentially regulated in BTBR hippocampus compared to B6 controls, are depicted. The pathway enrichment probability (stated as –log_10_(p-value)) and enrichment ratio are stated. The percentages of the total pathway genelist populated by down- (Downregulated) or upregulated (Upregulated) transcripts from the input datasets are also indicated.

| **Canonical Signaling Pathway** | **-log(p-value)** | **Ratio** | **Downregulated** | **Upregulated** |
| --- | --- | --- | --- | --- |
| Mitochondrial Dysfunction | 7.05E+00 | 5.26E-02 | 0/171 (0%) | 9/171 (5%) |
| Oxidative Phosphorylation | 6.17E+00 | 6.42E-02 | 0/109 (0%) | 7/109 (6%) |
| Signaling by Rho Family GTPases | 4.92E+00 | 3.42E-02 | 2/234 (1%) | 6/234 (3%) |
| RhoGDI Signaling | 4.83E+00 | 4.05E-02 | 2/173 (1%) | 5/173 (3%) |
| Ephrin Receptor Signaling | 4.82E+00 | 4.02E-02 | 3/174 (2%) | 4/174 (2%) |
| CCR3 Signaling in Eosinophils | 3.70E+00 | 4.27E-02 | 2/117 (2%) | 3/117 (3%) |
| Ephrin B Signaling | 3.45E+00 | 5.48E-02 | 1/73 (1%) | 3/73 (4%) |
| PAK Signaling | 3.13E+00 | 4.49E-02 | 2/89 (2%) | 2/89 (2%) |
| Axonal Guidance Signaling | 3.08E+00 | 1.85E-02 | 3/433 (1%) | 5/433 (1%) |
| Ephrin A Signaling | 2.86E+00 | 6.25E-02 | 2/48 (4%) | 1/48 (2%) |
| Semaphorin Signaling in Neurons | 2.73E+00 | 5.66E-02 | 2/53 (4%) | 1/53 (2%) |
| RhoA Signaling | 2.62E+00 | 3.28E-02 | 1/122 (1%) | 3/122 (2%) |
| Actin Cytoskeleton Signaling | 2.49E+00 | 2.30E-02 | 2/217 (1%) | 3/217 (1%) |
| CXCR4 Signaling | 2.28E+00 | 2.63E-02 | 2/152 (1%) | 2/152 (1%) |
| G Protein Signaling Mediated by Tubby | 2.00E+00 | 6.06E-02 | 0/33 (0%) | 2/33 (6%) |
| IL-8 Signaling | 1.99E+00 | 2.17E-02 | 2/184 (1%) | 2/184 (1%) |
| Thrombin Signaling | 1.94E+00 | 2.09E-02 | 1/191 (1%) | 3/191 (2%) |
| Breast Cancer Regulation by Stathmin1 | 1.94E+00 | 2.09E-02 | 1/191 (1%) | 3/191 (2%) |
| Rac Signaling | 1.92E+00 | 2.88E-02 | 1/104 (1%) | 2/104 (2%) |
| fMLP Signaling in Neutrophils | 1.87E+00 | 2.78E-02 | 0/108 (0%) | 3/108 (3%) |
| Ascorbate Recycling (Cytosolic) | 1.86E+00 | 3.33E-01 | 0/3 (0%) | 1/3 (33%) |
| Glutathione Redox Reactions II | 1.86E+00 | 3.33E-01 | 0/3 (0%) | 1/3 (33%) |
| Methylmalonyl Pathway | 1.74E+00 | 2.50E-01 | 0/4 (0%) | 1/4 (25%) |
| 2-oxobutanoate Degradation I | 1.64E+00 | 2.00E-01 | 0/5 (0%) | 1/5 (20%) |
| Cardiac β-adrenergic Signaling | 1.63E+00 | 2.26E-02 | 1/133 (1%) | 2/133 (2%) |
| Actin Nucleation by ARP-WASP Complex | 1.57E+00 | 3.57E-02 | 1/56 (2%) | 1/56 (2%) |
| Regulation of eIF4 and p70S6K Signaling | 1.53E+00 | 2.05E-02 | 0/146 (0%) | 3/146 (2%) |
| Gαq Signaling | 1.52E+00 | 2.04E-02 | 1/147 (1%) | 2/147 (1%) |
| Inositol Pyrophosphates Biosynthesis | 1.50E+00 | 1.43E-01 | 1/7 (14%) | 0/7 (0%) |
| Antiproliferative Role of Somatostatin Receptor 2 | 1.47E+00 | 3.17E-02 | 0/63 (0%) | 2/63 (3%) |
| Tec Kinase Signaling | 1.44E+00 | 1.90E-02 | 1/158 (1%) | 2/158 (1%) |
| CCR5 Signaling in Macrophages | 1.40E+00 | 2.90E-02 | 0/69 (0%) | 2/69 (3%) |
| Calcium Transport I | 1.39E+00 | 1.11E-01 | 0/9 (0%) | 1/9 (11%) |
| Cdc42 Signaling | 1.38E+00 | 1.80E-02 | 1/167 (1%) | 2/167 (1%) |
| Calcium Signaling | 1.32E+00 | 1.69E-02 | 0/178 (0%) | 3/178 (2%) |
