# Supplementary material for: Hippocampal Transcriptomic and Proteomic Alterations in the BTBR Mouse Model of Autism Spectrum Disorder
Source: Front Physiol. 2015 Nov 24;6:324. doi: 10.3389/fphys.2015.00324 (PMC4656818; doi:10.3389/fphys.2015.00324)
Supplement: Supplementary file 16 [file Table15.DOCX]

**Table S15. GeneIndexer BTBR hippocampus protein analysis.** The respective implicitly-associated proteins linked to each input interrogator term (autism, autistic disorder, autistic spectrum disorder, ADHD, ASD, obsessive) column are depicted. Each numerical value represents the Cosine Similarity score for the specific transcript-word correlation. Only transcripts demonstrating implicit association (Cosine Similarity score > 0.1) with at least two different interrogator terms are shown.

| **Protein** | **autism** | **autistic disorder** | **autistic spectrum disorder** | **ADHD** | **ASD** | **obsessive** |
| --- | --- | --- | --- | --- | --- | --- |
| Uqcrc1 | 0.322 | 0.347 | 0.387 | 0 | 0.188 | 0.14 |
| Lppr4 | 0.259 | 0.225 | 0.235 | 0 | 0.103 | 0 |
| Fam120c | 0.218 | 0.194 | 0.216 | 0 | 0.154 | 0 |
| Slc25a22 | 0.185 | 0.167 | 0.213 | 0 | 0 | 0.13 |
| Bsn | 0.205 | 0.16 | 0.173 | 0 | 0.108 | 0 |
| Wfs1 | 0.118 | 0.125 | 0.178 | 0 | 0 | 0.168 |
| Ogfod1 | 0.197 | 0.133 | 0.137 | 0 | 0.103 | 0 |
| Cend1 | 0.117 | 0.106 | 0.123 | 0 | 0.126 | 0 |
| Opcml | 0.105 | 0 | 0.138 | 0.113 | 0.107 | 0 |
| Cacng8 | 0.119 | 0.106 | 0.117 | 0 | 0 | 0.111 |
| Atp2b2 | 0.129 | 0.112 | 0.142 | 0 | 0 | 0 |
| Trappc8 | 0.122 | 0.105 | 0.146 | 0 | 0 | 0 |
| Rnf214 | 0.13 | 0 | 0.107 | 0 | 0 | 0.106 |
| Fkbp8 | 0 | 0.137 | 0.141 | 0 | 0 | 0 |
| Dek | 0 | 0.105 | 0.124 | 0 | 0 | 0 |
| Dync1i1 | 0.106 | 0 | 0.114 | 0 | 0 | 0 |
| Cox4i1 | 0 | 0 | 0 | 0 | 0 | 0.156 |
| Mut | 0 | 0 | 0.111 | 0 | 0 | 0 |
| Enah | 0 | 0 | 0.109 | 0 | 0 | 0 |
| Gipc3 | 0 | 0 | 0 | 0.109 | 0 | 0 |
| Atp5j2 | 0 | 0 | 0.103 | 0 | 0 | 0 |
| Hnrnpu | 0 | 0 | 0.102 | 0 | 0 | 0 |
| Tom1l2 | 0 | 0 | 0.102 | 0 | 0 | 0 |
| Pitpnm3 | 0 | 0 | 0 | 0 | 0 | 0.101 |
